# Supplementary material for: A fetal oncogene NUAK2 is an emerging therapeutic target in glioblastoma
Source: EMBO Mol Med. 2025 Aug 6;17(9):2409–37. doi: 10.1038/s44321-025-00287-3 (PMC12423323; doi:10.1038/s44321-025-00287-3)
Supplement: Supplementary file 1 — Appendix [file 44321_2025_287_MOESM1_ESM.pdf]

APPENDIX

Table of contents

Appendix Table S1. List of qPCR primers pg 1-2

Appendix Table S2. List of Antibodies pg 2-3

Appendix Table S3. Summary of statistical tests and p values pg 3-8

Appendix Table S1. List of qPCR Primers

Target	Sequence (5' to 3')
ADAMTS14-F	CACTACCACGACACTCC
ADAMTS14-R	TTCCTTCCCGAAAACAGT[illegible]
ADAMTS20-F	CAAGGCCGTCGTCAGCT
ADAMTS20-R	TGGCTTGTTGTGACCAT[illegible]
COL13A1-F	GGAGACGGCTATTTTGG
COL13A1-R	TCCTTGAGTGGAGCTTC[illegible]
COL14A1-F	CTTGCACAGTATAGTGG[illegible]
COL14A1-R	AGTCCTTGATCCTGCTTC
FAP-F	CAAAGGCTGGAGCTAAG[illegible]
FAP-R	ACTGCAAACATACTCGTT
FBLN1-F	AGAGCTGCGAGTACAGC[illegible]
FBLN1-R	CGACATCCAAATCTCCG[illegible]
FBLN5-F	CTACTCGAACCCCTACT
FBLN5-R	TCGTGGGATAGTTTGGA[illegible]
HAS2-F	TCCTGGATCTCATTCCTC
HAS2-R	TGCACTGAACACACCCA
h*GAPDH*-F	TCAAGGCTGAGAACGGG[illegible]
h*GAPDH*-R	CGCCCCACTTGATTTTG
h*NUAK1*-F	GGGAGCTGTACGATTAC[illegible]

h*NUAK1*-R	ACACCGTTCTTGTGACA
h*NUAK2*-F	CTTGCTCACCTCCTGCT [illegible]
h*NUAK2*-R	CTTCACCGCCTGCTTCT
m*GAPDH*-F	CATGGCCTTCCGTGTTC
m*GAPDH*-R	CTGGTCCTCAGTGTAGC [illegible]
MMP14-F	CGAGGTGCCCTATGCCT
MMP14-R	CTCGGCAGAGTCAAAGT [illegible]
MMP7-F	GAGTGAGCTACAGTGGG
MMP7-R	CTATGACGCGGGAGTTT
m*NUAK1*-F	TCCAACCTGTACCAGAA [illegible]
m*NUAK1*-R	GGGCATCGTTCCATAAA
m*NUAK2*-F	GCATTTCTTCCGACAGAT [illegible]
m*NUAK2*-R	ACAGAACGTCTGGAGGA
TNFRSF11B-F	GTGTGCGAATGCAAGGA
TNFRSF11B-R	CCACTCCAAATCCAGGA [illegible]

Appendix Table S2. List of Antibodies

Name	Company	Cat. #	Application
anti-Alpha tubulin	GeneTex	GTX628802	WB (1:1000)
Anti-Beta Actin	Sigma	A5316	WB (1:1000)
anti-E-Cadherin	CST	3195	WB (1:500)
Anti-CD44	Abcam	Ab189524	IHC (1:2000)
Anti-CDK2	CST	2546	WB (1:1000)
Anti-CDK6	CST	3136	WB (1:2000)
Anti-CDK4	CST	12790	WB (1:1000)
Anti-Cyclin D1	CST	2978	WB (1:1000)
Anti-Cyclin D3	CST	2936	WB (1:2000)
Anti-EdU	Thermo	C10639	IHC (1:500)
Anti-EFTUD2	Novus	NBP1-	WB (1:500)

anti-GAPDH	Millipore	MAB374	WB (1:1000)
Anti-HSP90	stressMarq	SMC-137	WB (1:1000)
anti-Ki67	CST	12202S	IHC (1:500), ICC (1:500)
anti-N-Cadherin	CST	13116	WB (1:500)
anti-NUAK1	CST	4458S	WB (1:500)
anti-NUAK2	abcam	ab224079	WB (1:1000), ICC (1:100), IHC (1:50)
Anti-P18	CST	2896	WB (1:1000)
Anti-P21	CST	2947	WB (1:300)
Anti-P27	CST	3686	WB (1:1000)
Anti-PCNA	Sigma	MAB424	IHC (1:200)
Anti-MYPT1	ProteinTe	22117-1-	WB (1:2500)
Anti-YAP1	CST	14074	WB (1:500)
Anti-TAZ	CST	4883	WB (1:500)
Anti-LATS1	CST	C66B5	WB (1:500)
Anti-Stem121	Takara	Y40410	IHC (1:200)
anti-Slug	CST	9585	WB (1:500)
anti-Snail	CST	3879	WB (1:500)
anti-Vimentin	CST	5741	WB (1:500)
anti-ZEB1	CST	3396	WB (1:500)
anti-ZO-1	CST	8193	WB (1:500)
anti-β-Catenin	CST	8480	WB (1:500)

Appendix Table S3. Summary of statistical tests and p values

Figure #	Statistical method	Multiple comparison	Groups	P value	summary
**Figure 1**					
1B	One-way ANOVA	N/A	Normal vs Tumor	0.01	*
1C	One-way ANOVA	Tukey's multiple comparisons test	Oligodendroglioma vs GBM	6.18E-06	****
			Oligoastrocytoma vs GBM	6.18E-06	****
			Astrocytoma vs GBM	4.4E-10	****
			Anaplastic Oligodendroglioma vs GBM	4.4E-10	****

			Anaplastic Oligoastrocytoma vs GBM	2.5E-07	****
			Anaplastic Astrocytoma vs GBM	5.4E-07	****
1D	One-way ANOVA	Tukey's multiple comparisons test	Oligodendroglioma vs GBM	1e-14	****
			Oligoastrocytoma vs GBM	1e-14	****
			Astrocytoma vs GBM	1e-14	****
1E	Log-rank (Mantel-Cox) test		NUAK2 High vs Low GBM	1e-14	****
1F	Log-rank (Mantel-Cox) test		NUAK2 High vs Low GBM/LGG	1.1E-09	****
			TCGA NUAK2 High vs Low GBM	0.0265	*
			TCGA NUAK2 High vs Low Astrocytoma	0.0002	***
			TCGA NUAK2 High vs Low Oligodendroglioma	0.0022	**
**Figure 2**					
2D	One-way ANOVA	Dunnett's multiple comparison test	WT vs. CR1	7.7E-05	****
			WT vs. CR2	7.7E-05	****
			WT vs. CR3	7.5E-05	****
2E	One-way ANOVA	Dunnett's multiple comparison test	WT vs. CR1	4.6E-07	****
			WT vs. CR2	1.1E-07	****
			WT vs. CR3	0.0159	*
2F	One-way ANOVA	Dunnett's multiple comparison test	WT vs. CR1	0.7038	ns
			WT vs. CR2	3.2E-06	****
			WT vs. CR3	2.7E-13	****
2G	Two-way RM ANOVA	Uncorrected Fisher's LSD	Day5_WT vs. CR1	0.3654	ns
			Day5_WT vs. CR2	0.0033	**
			Day5_WT vs. CR3	0.002	**
2H	One-way ANOVA	Dunnett's multiple comparison test	WT vs. CR1, 2, 3	1.7E-09	****
**Figure 3**					
3C	Two-way RM ANOVA	Uncorrected Fisher's LSD	U87_Day5_WT vs N2OE	0.0068	**
			LN229_Day5_WT vs N2OE	0.0001	***
3D	Unpaired t test (two-tailed)	N/A	U87_WT vs N2OE	8.4E-05	****
		N/A	LN229_WT vs N2OE	0.0002	***
3G	Unpaired t test (two-tailed)	Welch's	GSC11 GFP vs N2OE	9.1E-05	****
	Unpaired t test (two-tailed)	Welch's	GSC23 GFP vs N2OE	5.8E-05	****

3H	Unpaired t test (two-tailed)	Welch's	GSC11 GFP vs N2OE	0.0165	*
	Unpaired t test (two-tailed)	Welch's	GSC23 GFP vs N2OE	0.0003	***
3J	Unpaired t test (two-tailed)	Welch's	GSC11 GFP vs N2OE	4.4E-05	****
	Unpaired t test (two-tailed)	Welch's	GSC23 GFP vs N2OE	9.8E-07	****
**Figure 4**					
4B	RM Two-way ANOVA with the Geisser-Greenhouse correction	Uncorrected Fisher's LSD, with inividual variances computed for each comparison	WT_7dpi vs 28dpi	0.036	*
			28dpi_WT vs. N2CR	0.0044	**
4C	Log-rank (Mantel-Cox) test			0.0007	***
4D	Unpaired t test (two-tailed)	N/A	WT vs NUAK2 CR	0.0062	**
**Figure 5**					
5C	Log-rank (Mantel-Cox) test		3XCR vs. N2CR	0.001	**
5D	Log-rank (Mantel-Cox) test		3XCR vs. N2OE	0.0004	***
5F	One-way ANOVA	Dunnett's multiple comparison test	3XCR vs. N2CR	1e-14	****
			3XCR vs. N2OE	0.0015	**
5G	One-way ANOVA	Dunnett's multiple comparison test	3XCR vs. N2CR	2.4E-06	****
			3XCR vs. N2OE	0.0282	*
5H	One-way ANOVA	Dunnett's multiple comparison test	3XCR vs. N2CR	1.00E-14	****
			3XCR vs. N2OE	2.3E-04	
5I	One-way ANOVA	Dunnett's multiple comparison test	3XCR vs. N2CR	1.10E-07	****
			3XCR vs. N2OE		
**Figure 6**					
6F	Unpaired t test (two-tailed)	N/A	MMP7	0.02	*
		N/A	FBLN1	0.002	***
		N/A	TNFRSF11B	0.003	***
		N/A	MMP14	0.003	***
		N/A	FBLN5	1.9E-05	****
		N/A	FAP	0.002	***
		N/A	COL14A1	0.008	***

		N/A	ADAMTS14	0.02	*
		N/A	HAS2	0.1718	ns
		N/A	COL13A1	0.017	*
6G	One-way ANOVA	Dunnett's multiple comparison test	WT vs. CR1	4.1E-14	****
			WT vs. CR2	1E-15	****
			WT vs. CR3	1E-15	****
6H	Unpaired t test (two-tailed)	N/A	U87_WT vs N2OE	1.2E-05	****
			LN229_WT vs N2OE	7.9E-05	****
**Figure 7**					
7A	Two-way ANOVA	Dunnett's multiple comparison test	Day4_0uM vs. 0.5uM	0.0093	**
			Day4_0uM vs. 1uM	0.0034	**
			Day4_0uM vs. 2.5uM	5.2E-14	****
			Day4_0uM vs. 5uM	2.8E-14	****
			Day4_0uM vs. 10uM	2.8E-14	****
7B	One-way ANOVA	Dunnett's multiple comparison test	0uM vs. 1uM	0.083	ns
			0uM vs. 5uM	3.8E-08	****
			0uM vs. 10uM	1.8E-09	****
7C	RM Two-way ANOVA with the Geisser-Greenhouse correction	Uncorrected Fisher's LSD, with inividual variances computed for each comparison	72h_0uM vs. 5uM	0.0008	***
7D	Unpaired t test (two-tailed)		HTH 0uM vs 5uM	6.7E-06	****
7F	One-way ANOVA	Dunnett's multiple comparison test	Day6 0 vs 1	0.0006	***
			Day6 0 vs 2.5	0.0201	*
			Day6 0 vs 5	0.0001	***
			Day6 0 vs 10	1.5E-06	****
7G	One-way ANOVA	Tukey's multiple comparisons test	Day6 0 vs 1	3E-05	****
			Day6 0 vs 2.5	1.2E-06	****
			Day6 0 vs 5	2.6E-10	****
			Day6 0 vs 10	2.6E-10	****
**Extended Figures**					
**EV1**					
EV1A	RM Two-way ANOVA with the Geisser-Greenhouse correction	Dunnett's multiple comparisons test	3XCR vs. N2CR	0.0001	***
			3XCR vs. N2OE	0.0001	***
EV1B	RM Two-way ANOVA	Dunnett's multiple comparisons test	3XCR vs. N2CR	3.54E-10	****

	with the Geisser-Greenhouse correction		3XCR vs. N2OE	0.016	*
**EV3**					
EV3E	One-way ANOVA	N/A	GBM_Normal vs Tumor	0.01	*
			LGG_Normal vs Tumor	0.01	*
EV3F	One-way ANOVA	Tukey's multiple comparisons test	Astrocytoma vs. GBM	0.0193	*
EV3G	One-way ANOVA	Tukey's multiple comparisons test	Oligodendro vs. GBM	6.09E-07	****
			Oligoastrocytoma vs. GBM	2.87E-07	****
			Astrocytoma vs. GBM	0.0007	***
EV3H	Log-rank (Mantel-Cox) test		CGGA_NUAK2 High vs Low	0.682	ns
EV3I	Log-rank (Mantel-Cox) test		TCGA_NUAK2 High vs Low	0.6262	ns
EV3J	Spearman's correlation		TCGA GBM	0.6337	ns
**EV4**					
EV4C	one-way ANOVA	test for trend	U251 WT	1.0E-07	****
			U251 CR1	0.5818	ns
			U251 CR2	0.9177	ns
			U251 CR 3	0.0772	ns
EV4D	Two-way ANOVA	Dunnett's multiple comparison test	U87_Day4_0uM vs. 0.5uM	0.1863	ns
			U87_Day4_0uM vs. 1uM	5.1E-06	****
			U87_Day4_0uM vs. 2.5uM	0.074	ns
			U87_Day4_0uM vs. 5uM	2.8E-14	****
			U87_Day4_0uM vs. 10uM	2.8E-14	****
			LN229_Day4_0uM vs. 0.5uM	0.9811	ns
			LN229_Day4_0uM vs. 1uM	2.8E-14	****
			LN229_Day4_0uM vs. 2.5uM	2.8E-14	****
			LN229_Day4_0uM vs. 5uM	2.8E-14	****
			LN229_Day4_0uM vs. 10uM	2.8E-14	****
			LN319_Day4_0uM vs. 0.5uM	0.9292	ns
			LN319_Day4_0uM vs. 1uM	0.0008	***
			LN319_Day4_0uM vs. 2.5uM	8.4E-12	****
			LN319_Day4_0uM vs. 5uM	2.8E-14	****
			LN319_Day4_0uM vs. 10uM	2.8E-14	****
EV4E	One-way ANOVA	Dunnett's multiple comparison test	U87_Day4_0uM vs. 1uM	0.0669	ns
			U87_Day4_0uM vs. 5uM	0.0022	**
			U87_Day4_0uM vs. 10uM	2.1E-05	****
			LN229_Day4_0uM vs. 1uM	0.7949	ns
			LN229_Day4_0uM vs. 5uM	4.4E-06	****
			LN229_Day4_0uM vs. 10uM	1.1E-06	****

			LN319_Day4_0uM vs. 1uM	0.9998	ns
			LN319_Day4_0uM vs. 5uM	0.0072	**
			LN319_Day4_0uM vs. 10uM	4E-05	****
EV4F	RM Two-way ANOVA with the Geisser-Greenhouse correction	Dunnett's multiple comparisons test	U87_72h_0uM vs. 1uM	0.2693	ns
			U87_72h_0uM vs. 5uM	0.0026	**
			LN229_72h_0uM vs. 5uM	0.0012	**
			LN319_72h_0uM vs. 1uM	0.4229	ns
			LN319_72h_0uM vs. 5uM	0.0021	**
EV4G			U87_72h_0uM vs. 5uM	1E-15	****
			LN229_72h_0uM vs. 5uM	1E-15	****
			LN319_72h_0uM vs. 5uM	0.0006	***
**EV5**					
EV5B	One-way ANOVA	Dunnett's multiple comparison test	U87_Day4_0uM vs. 1uM	1.3E-06	****
			U87_Day4_0uM vs. 2.5uM	0.9999	ns
			U87_Day4_0uM vs. 5uM	4.6E-11	****
			U87_Day4_0uM vs. 10uM	1E-15	****
			LN229_Day4_0uM vs. 1uM	5.4E-09	****
			LN229_Day4_0uM vs. 2.5uM	3.3E-14	****
			LN229_Day4_0uM vs. 5uM	1E-15	****
			LN229_Day4_0uM vs. 10uM	1E-15	****
			LN319_Day4_0uM vs. 1uM	0.0022	**
			LN319_Day4_0uM vs. 2.5uM	0.0042	**
			LN319_Day4_0uM vs. 5uM	3.6E-05	****
			LN319_Day4_0uM vs. 10uM	2E-15	****
EV5C	One-way ANOVA	Dunnett's multiple comparison test	U87_Day4_0uM vs. 1uM	0.498	ns
			U87_Day4_0uM vs. 2.5uM	0.1575	ns
			U87_Day4_0uM vs. 5uM	0.0003	***
			U87_Day4_0uM vs. 10uM	3.7E-07	****
			LN229_Day4_0uM vs. 1uM	7E-05	****
			LN229_Day4_0uM vs. 2.5uM	3.95E-07	****
			LN229_Day4_0uM vs. 5uM	3.6E-14	****
			LN229_Day4_0uM vs. 10uM	3.3E-14	****
			LN319_Day4_0uM vs. 1uM	0.0039	**
			LN319_Day4_0uM vs. 2.5uM	0.0056	**
			LN319_Day4_0uM vs. 5uM	5.1E-09	****
			LN319_Day4_0uM vs. 10uM	1.8E-09	****
