# Supplementary figures and images for: A fetal oncogene NUAK2 is an emerging therapeutic target in glioblastoma
Source: EMBO Mol Med. 2025 Aug 6;17(9):2409–37. doi: 10.1038/s44321-025-00287-3 (PMC12423323; doi:10.1038/s44321-025-00287-3)

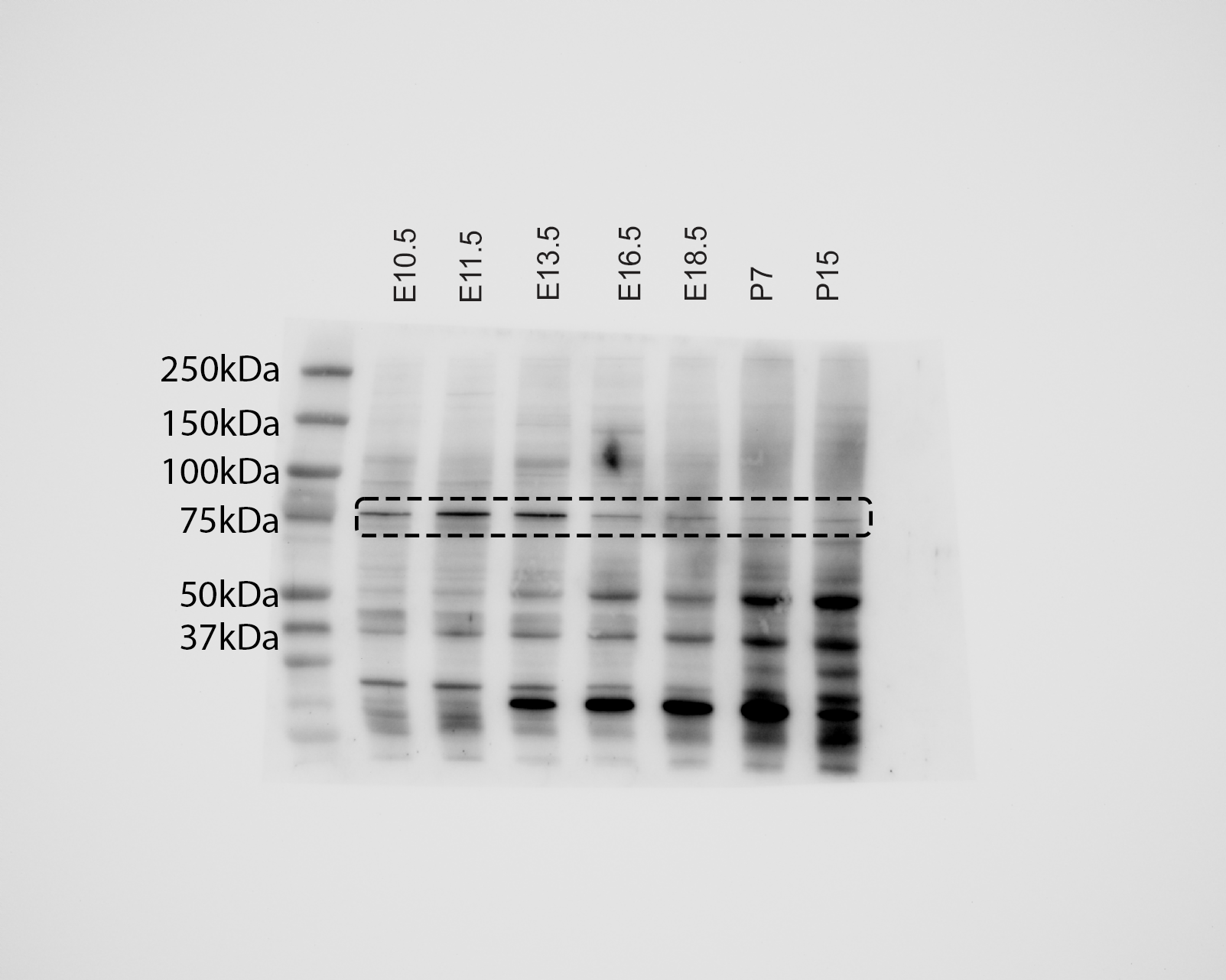

Supplement: Supplementary file 6 — Source data Fig. 1 [file 44321_2025_287_MOESM6_ESM.zip › Figure 1 /1H/Mouse Nuak2 Blots.tif]

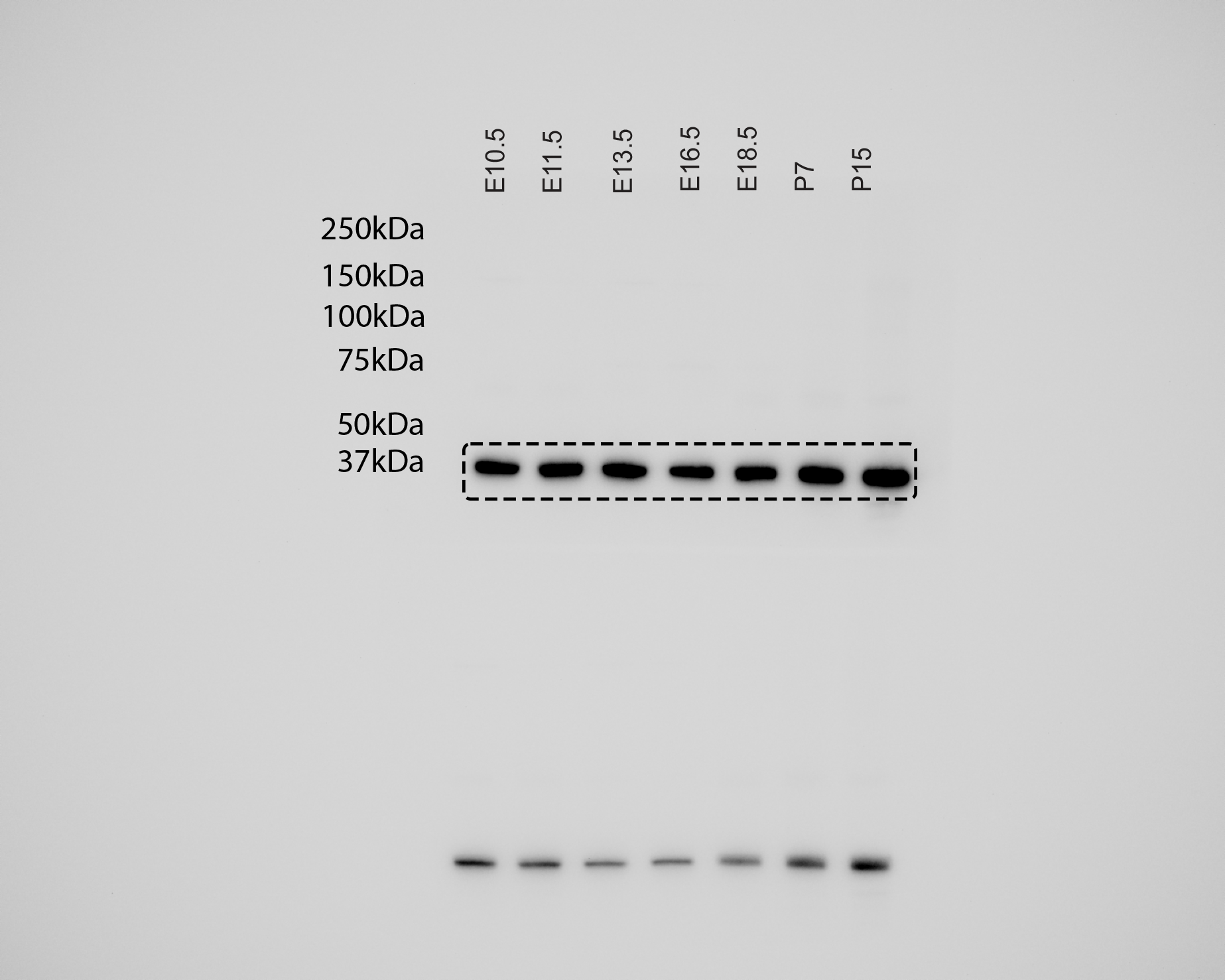

Supplement: Supplementary file 6 — Source data Fig. 1 [file 44321_2025_287_MOESM6_ESM.zip › Figure 1 /1H/the top blot is the gapdh for nuak2.tif]

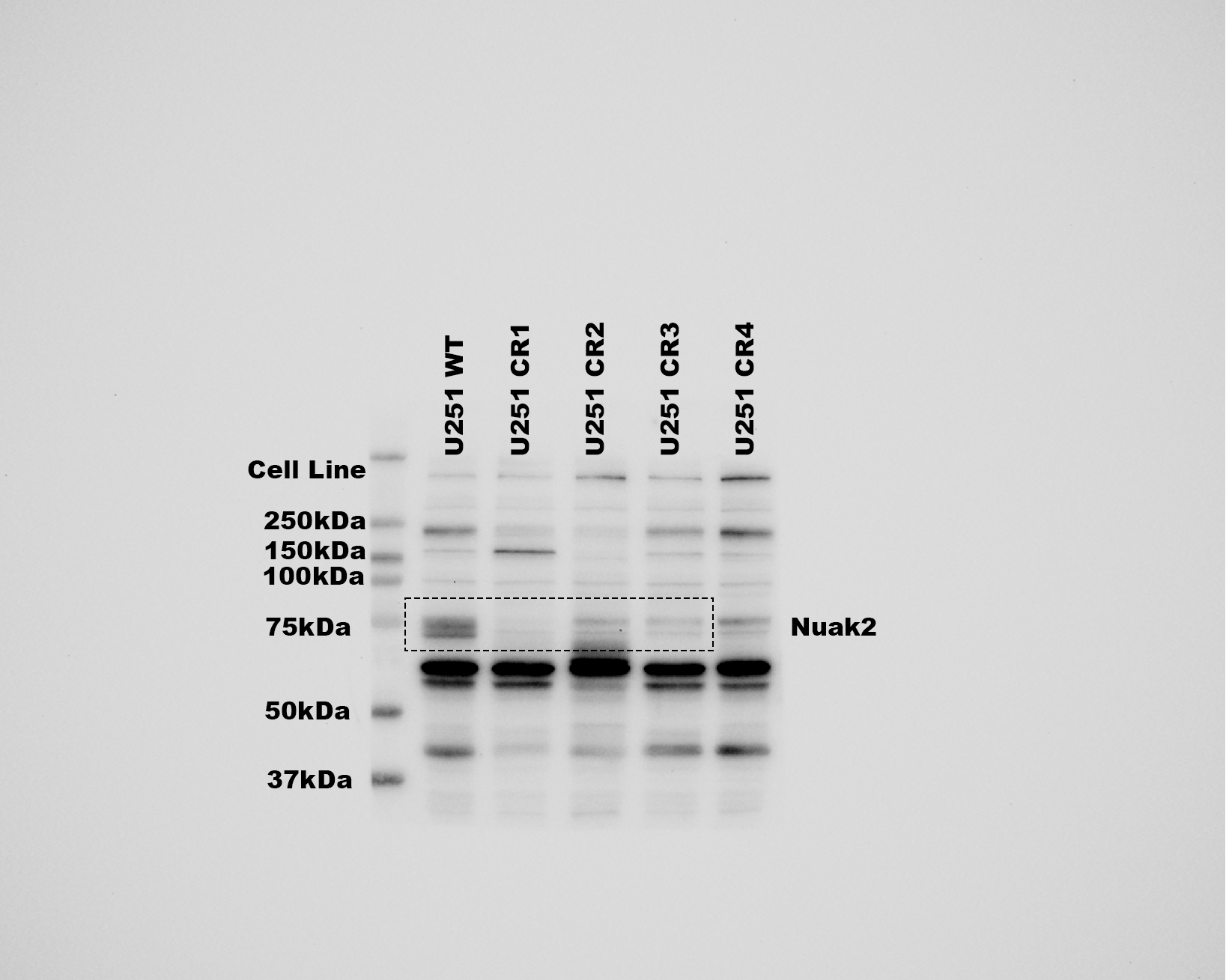

Supplement: Supplementary file 7 — Source data Fig. 2 [file 44321_2025_287_MOESM7_ESM.zip › Figure 2 /2C/Western Nuak2.tif]

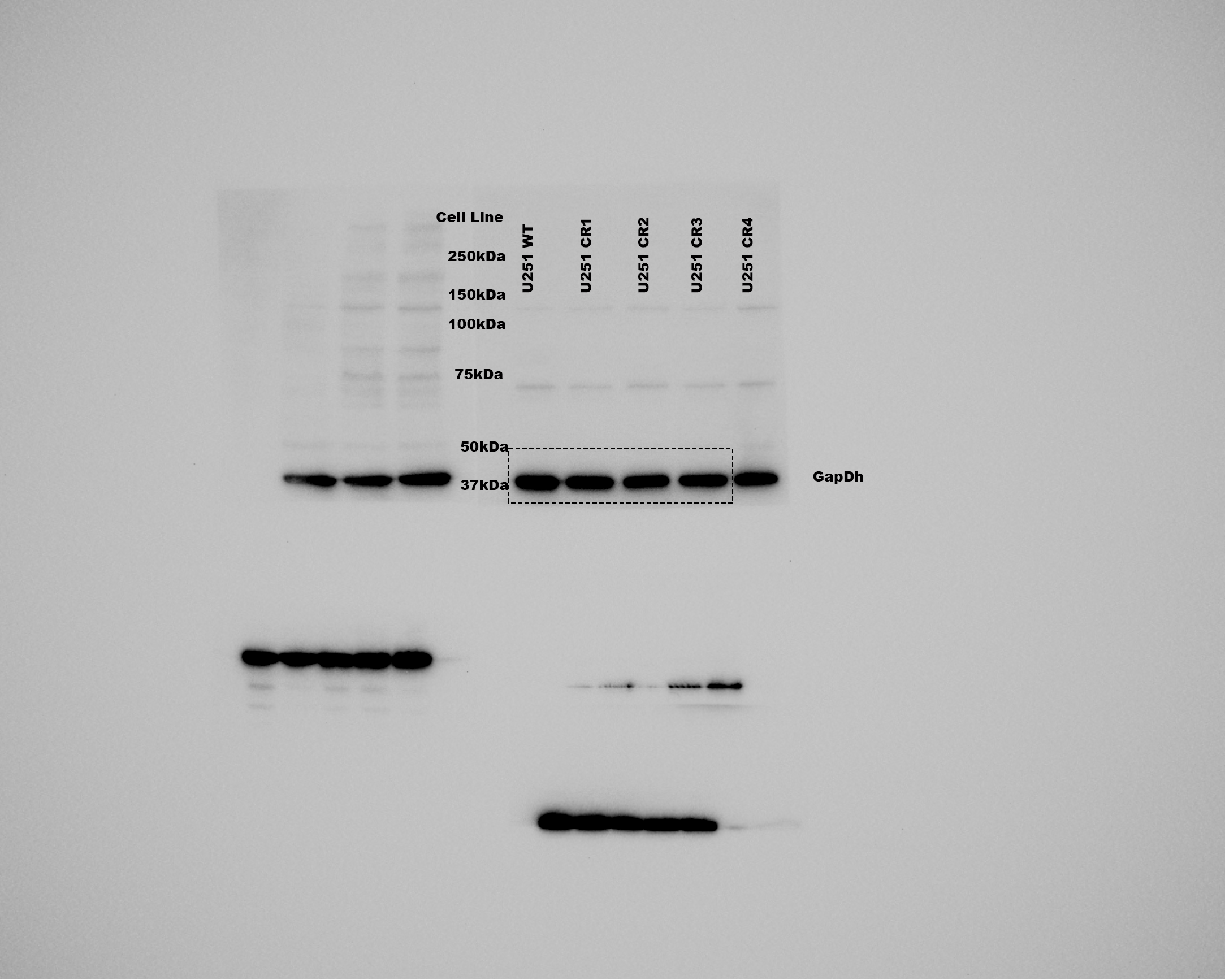

Supplement: Supplementary file 7 — Source data Fig. 2 [file 44321_2025_287_MOESM7_ESM.zip › Figure 2 /2C/Western GapdH.tif]

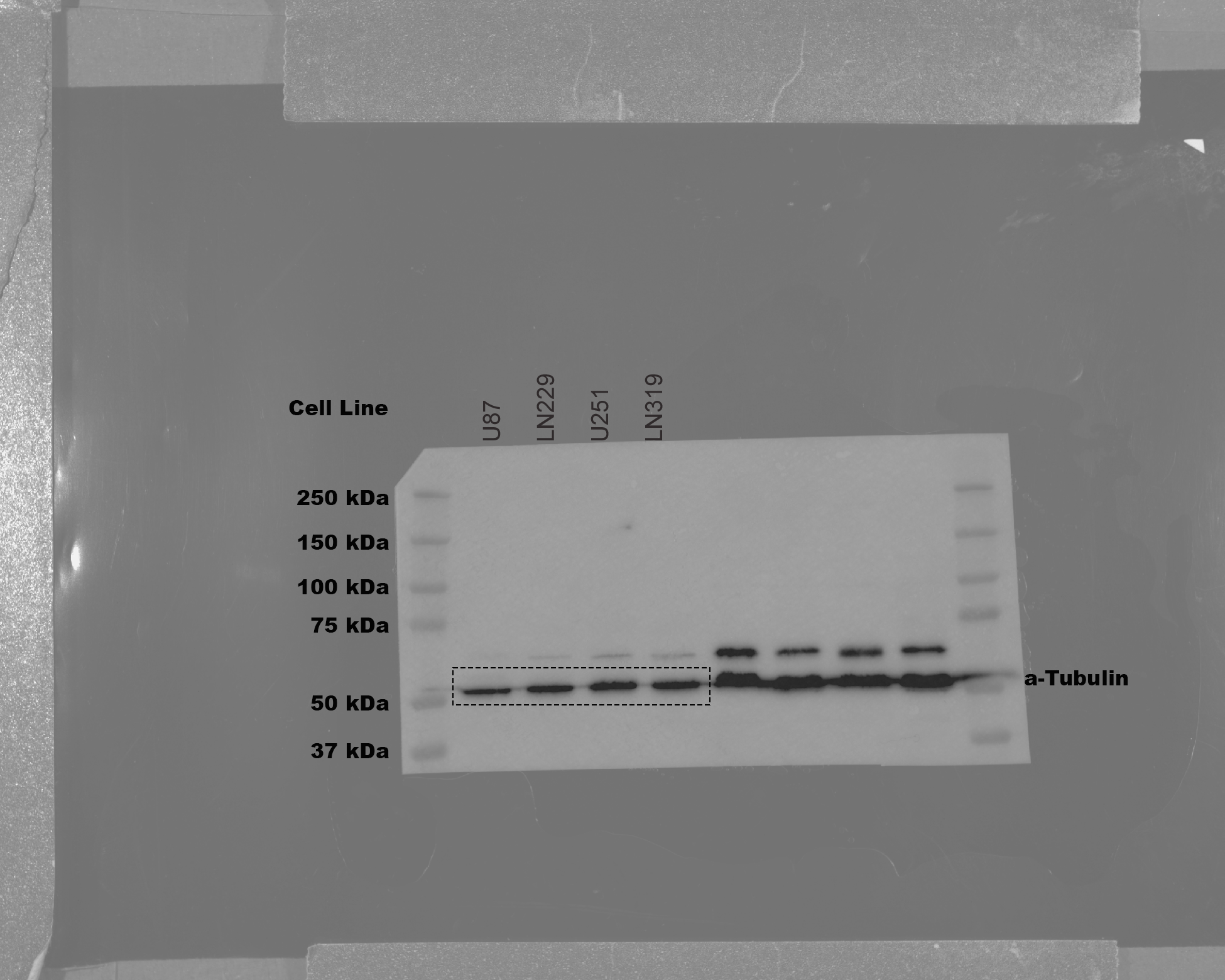

Supplement: Supplementary file 7 — Source data Fig. 2 [file 44321_2025_287_MOESM7_ESM.zip › Figure 2 /2A/Western Alpha-Tubulin.tif]

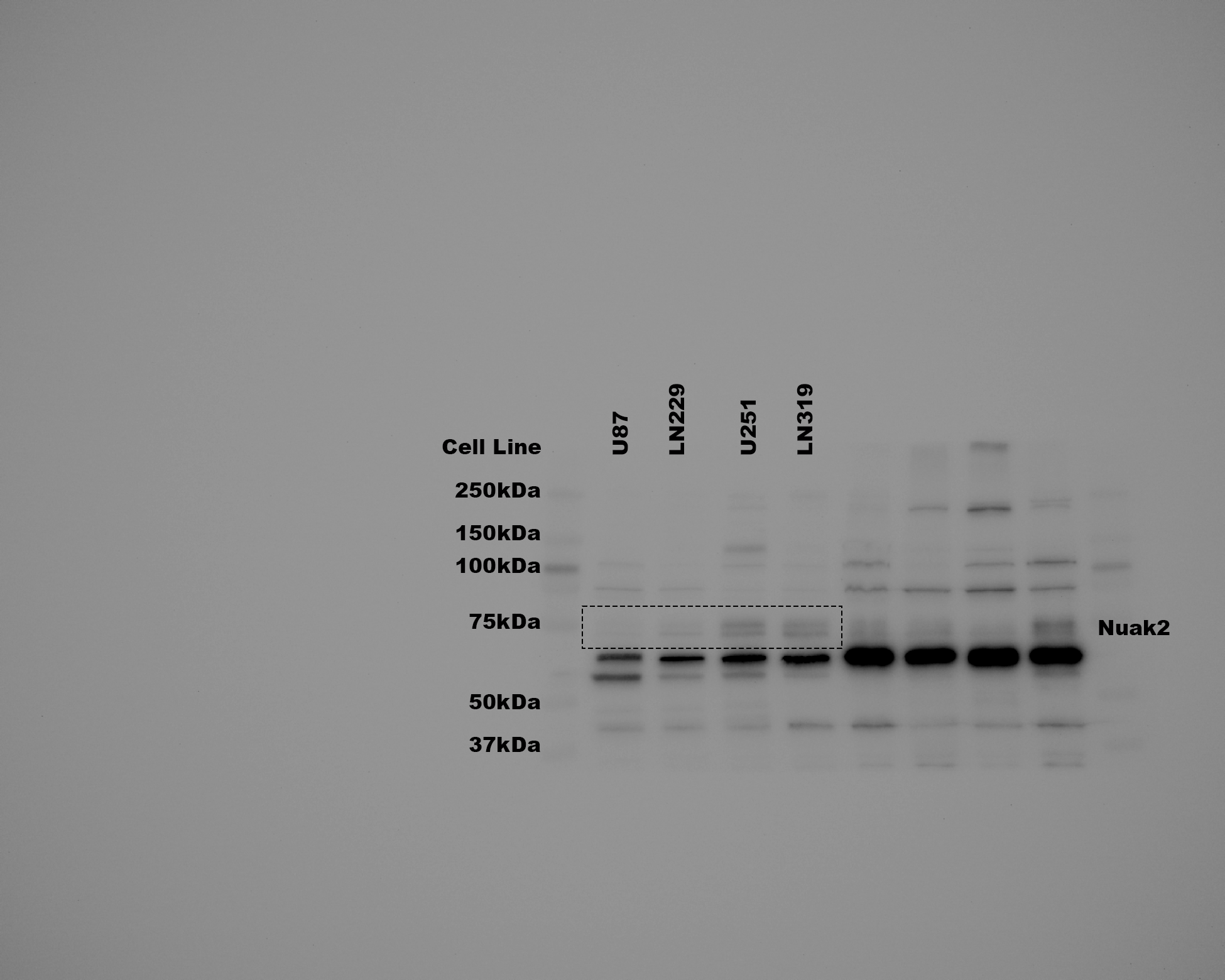

Supplement: Supplementary file 7 — Source data Fig. 2 [file 44321_2025_287_MOESM7_ESM.zip › Figure 2 /2A/Western Nuak2.tif]

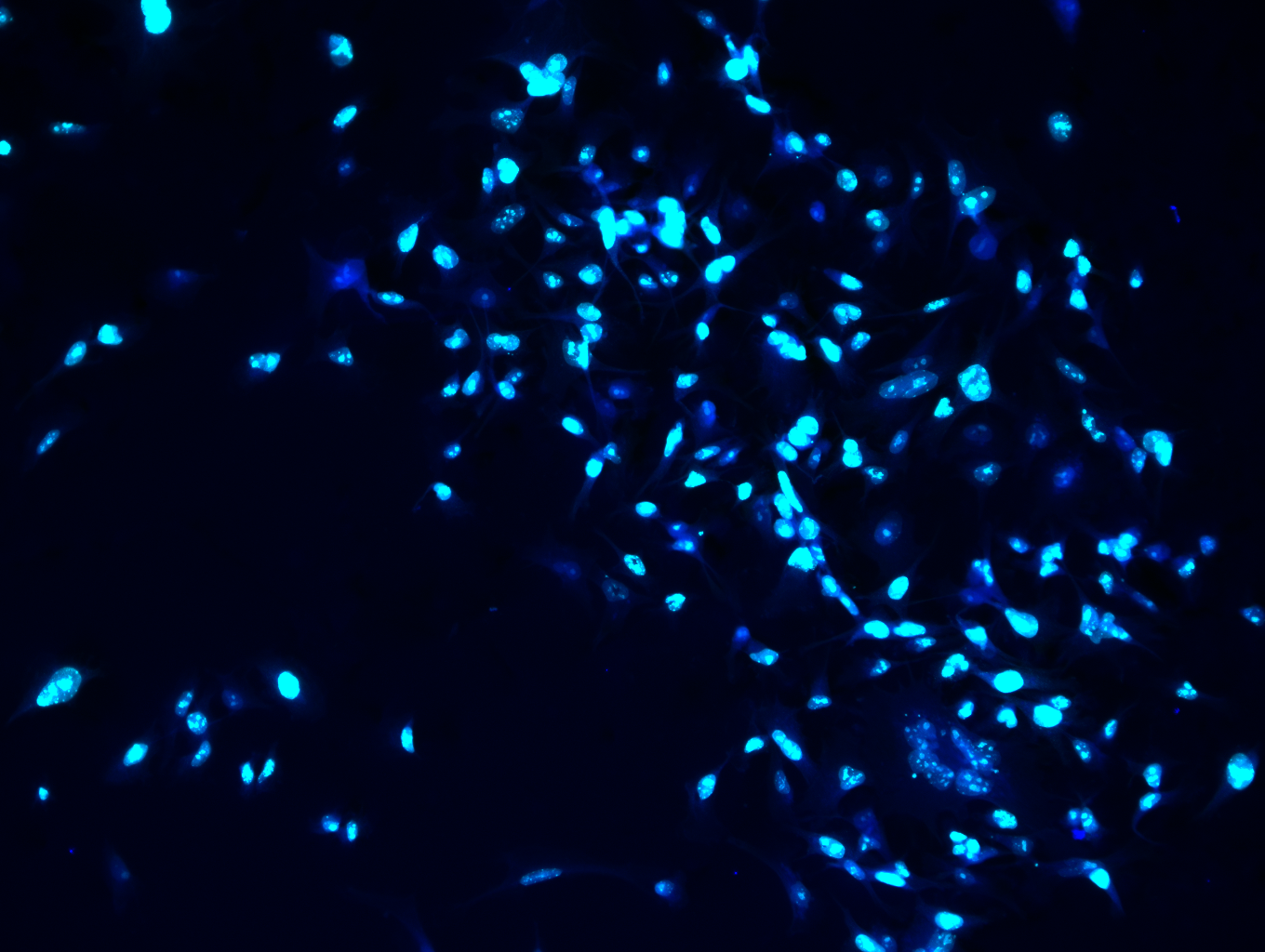

Supplement: Supplementary file 7 — Source data Fig. 2 [file 44321_2025_287_MOESM7_ESM.zip › Figure 2 /2E/ICC U251 WT MERGE.tif]

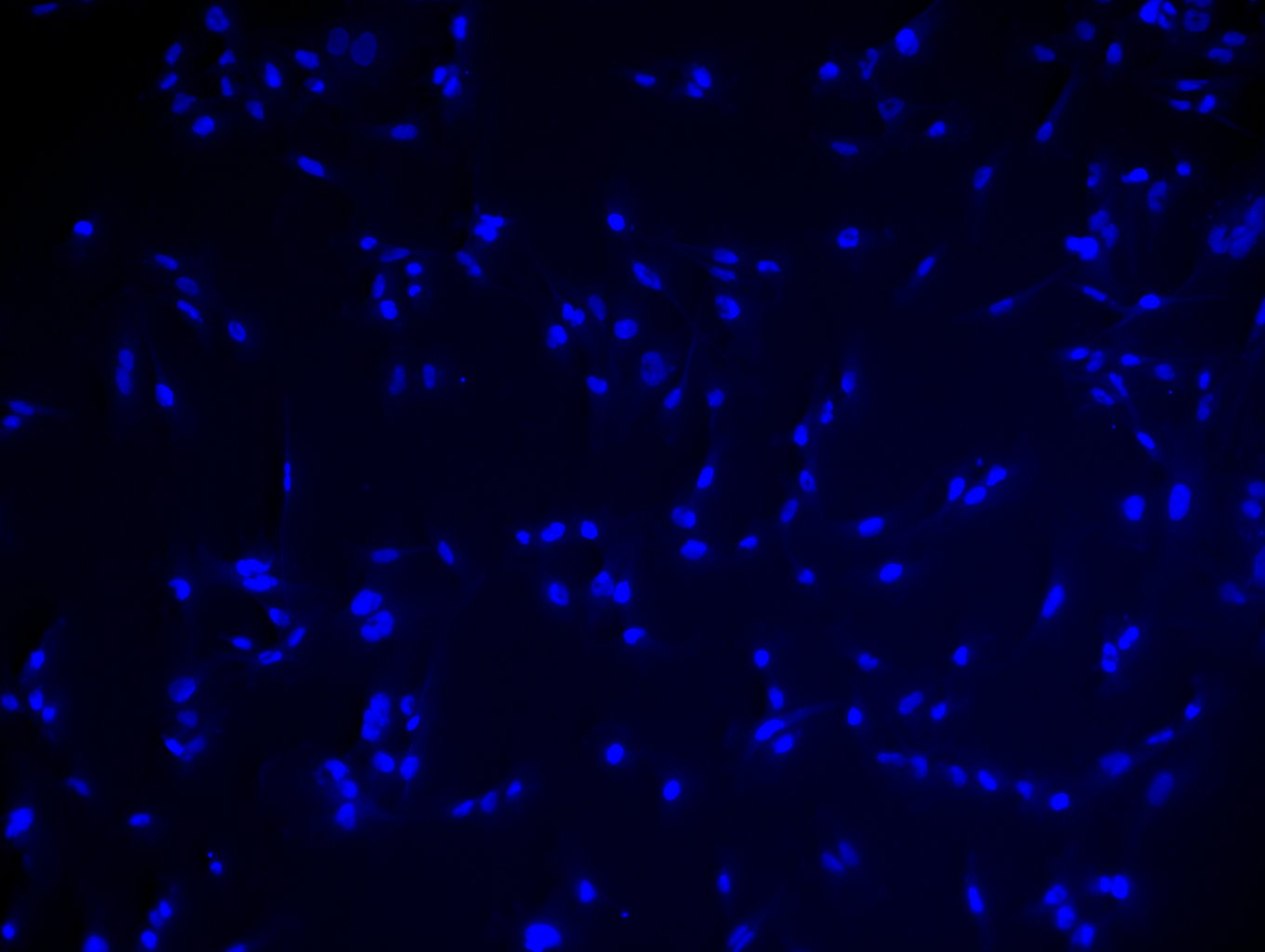

Supplement: Supplementary file 7 — Source data Fig. 2 [file 44321_2025_287_MOESM7_ESM.zip › Figure 2 /2E/ICC Image U251 CR1 DAPI.tif]

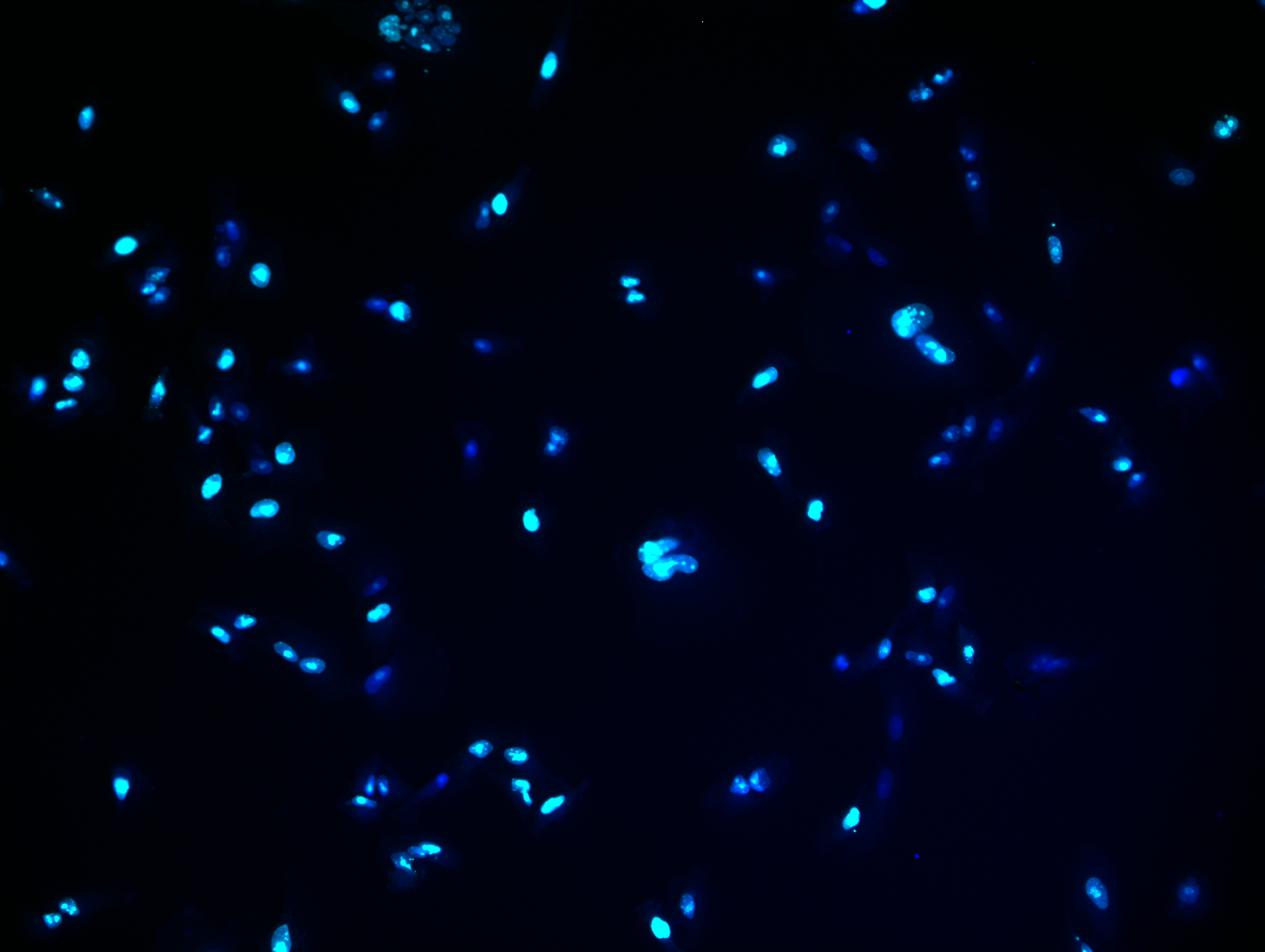

Supplement: Supplementary file 7 — Source data Fig. 2 [file 44321_2025_287_MOESM7_ESM.zip › Figure 2 /2E/ICC Image U251 CR2 MERGE.tif]

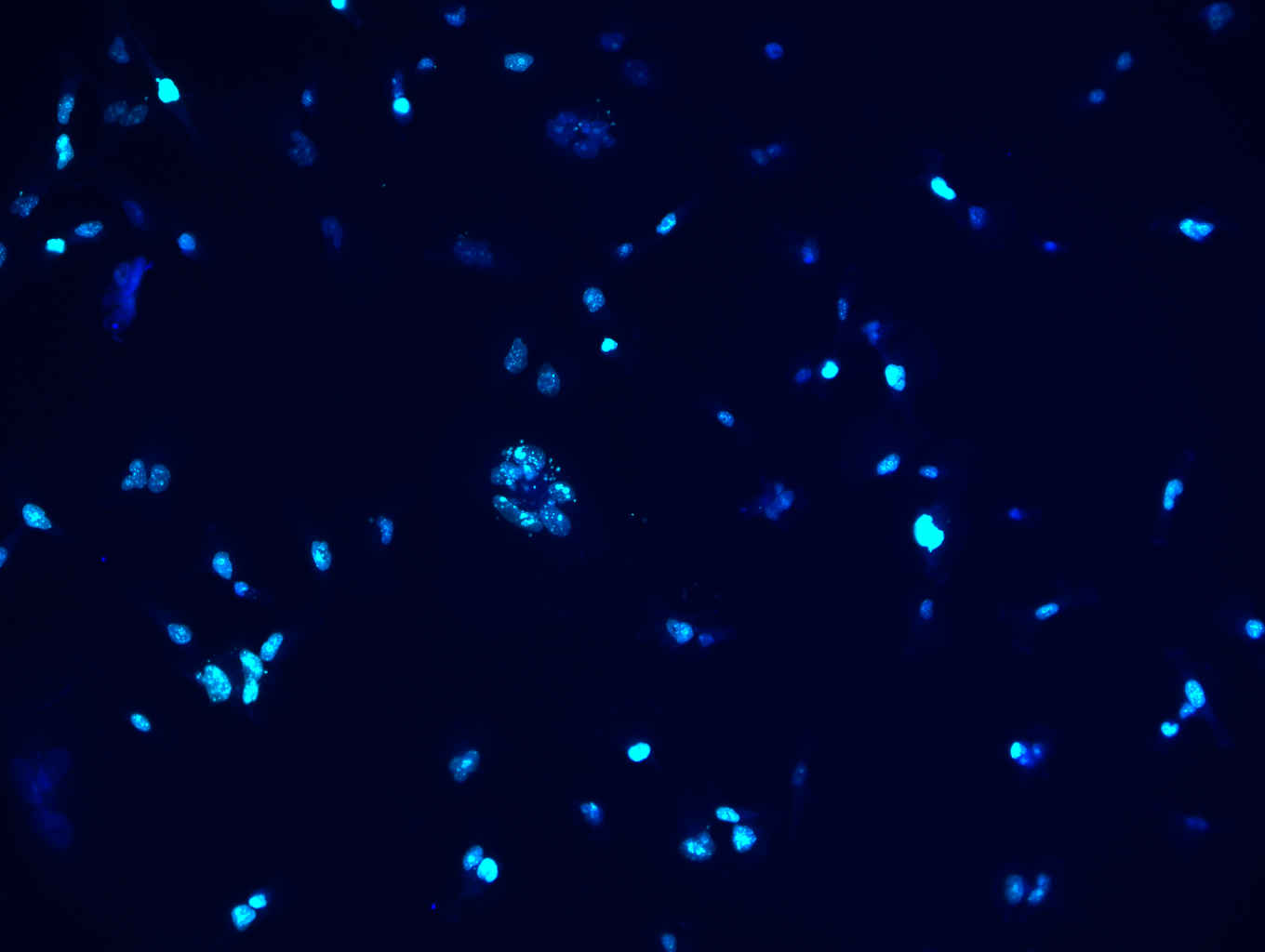

Supplement: Supplementary file 7 — Source data Fig. 2 [file 44321_2025_287_MOESM7_ESM.zip › Figure 2 /2E/ICC Image U251 CR3 MERGE.tif]

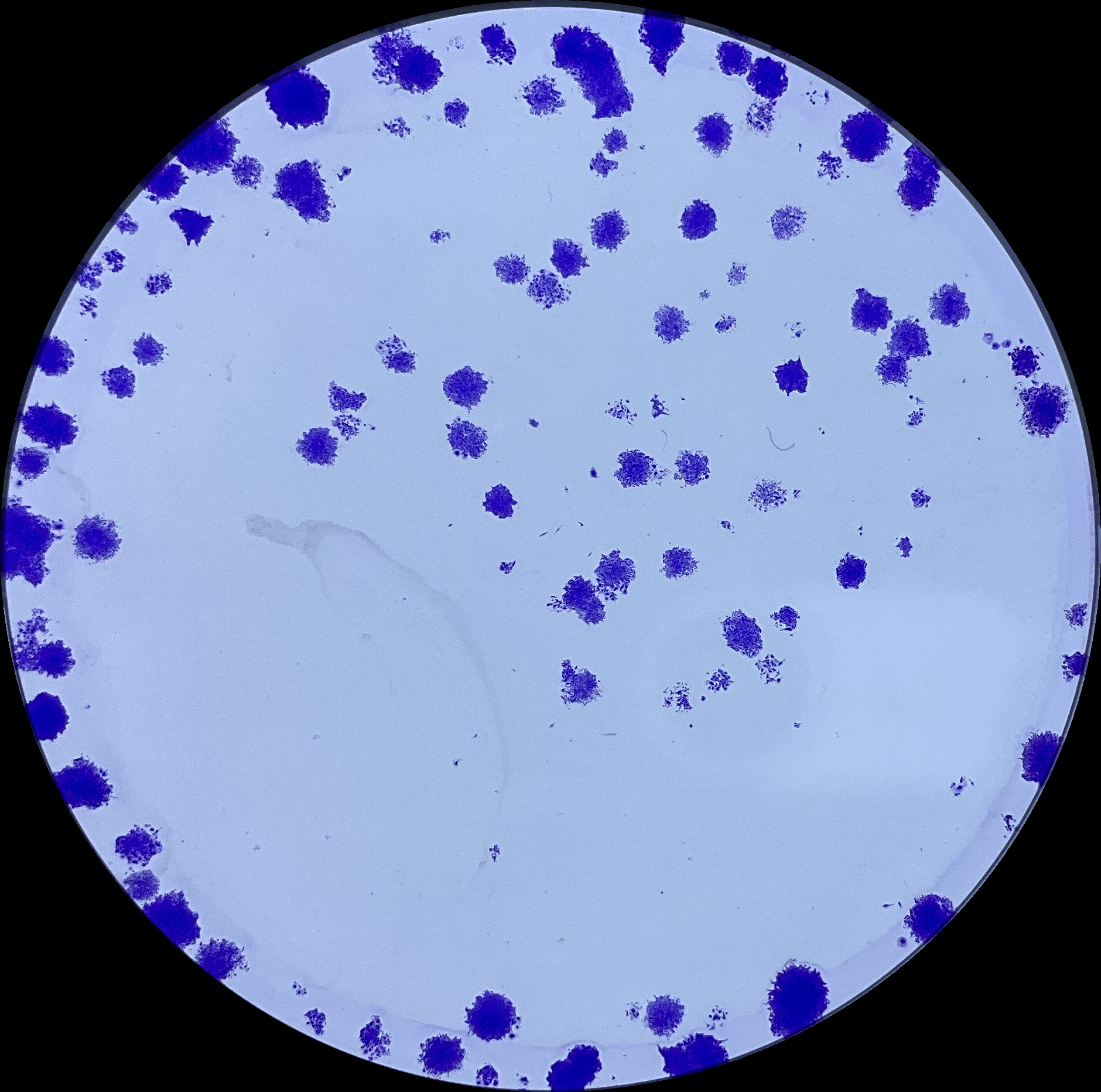

Supplement: Supplementary file 7 — Source data Fig. 2 [file 44321_2025_287_MOESM7_ESM.zip › Figure 2 /2H/Colony Formation Image U251 CR1.tif]

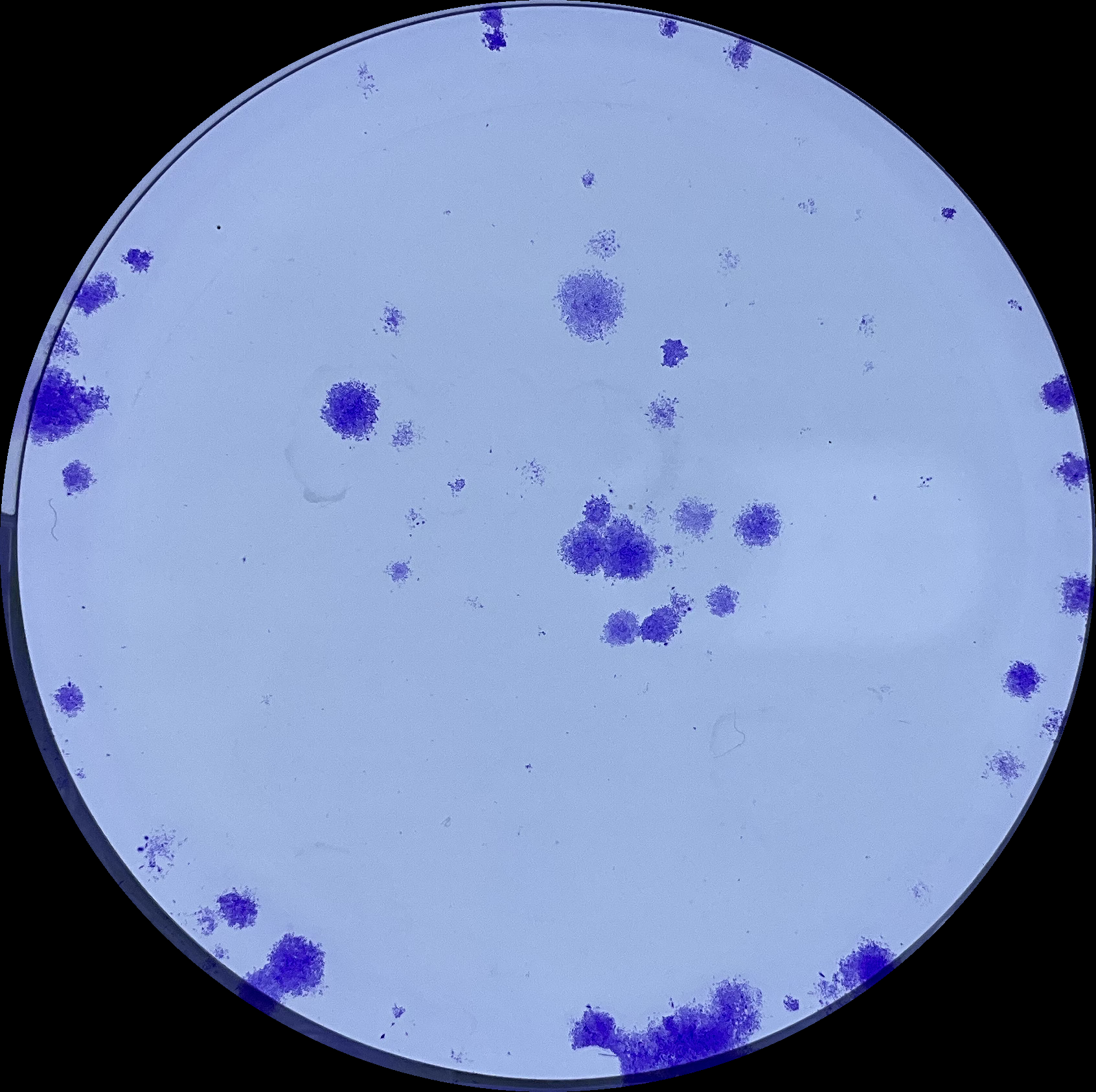

Supplement: Supplementary file 7 — Source data Fig. 2 [file 44321_2025_287_MOESM7_ESM.zip › Figure 2 /2H/Colony Formation Image U251 CR2.tif]

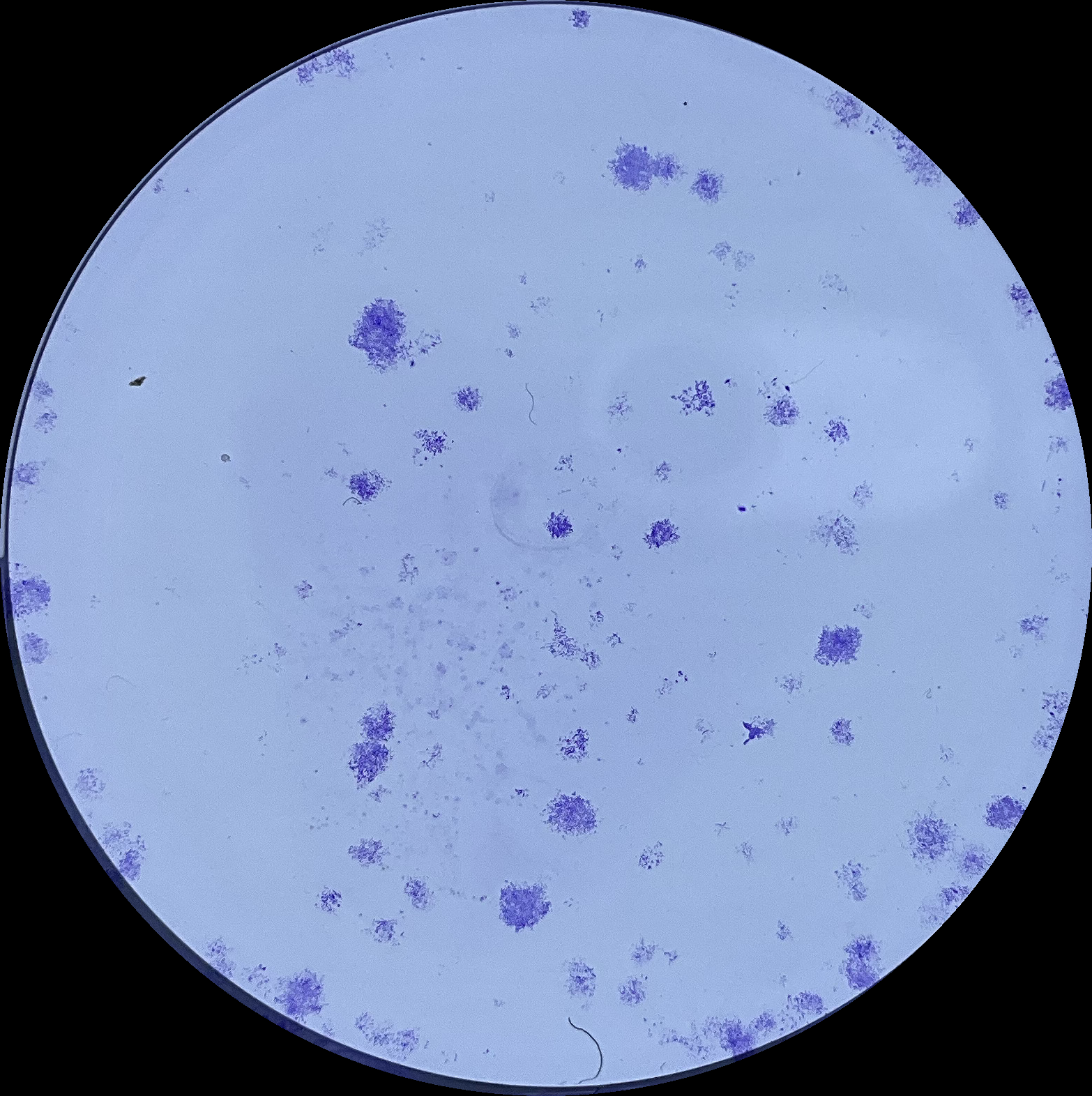

Supplement: Supplementary file 7 — Source data Fig. 2 [file 44321_2025_287_MOESM7_ESM.zip › Figure 2 /2H/Colony Formation Image U251 CR3.tif]

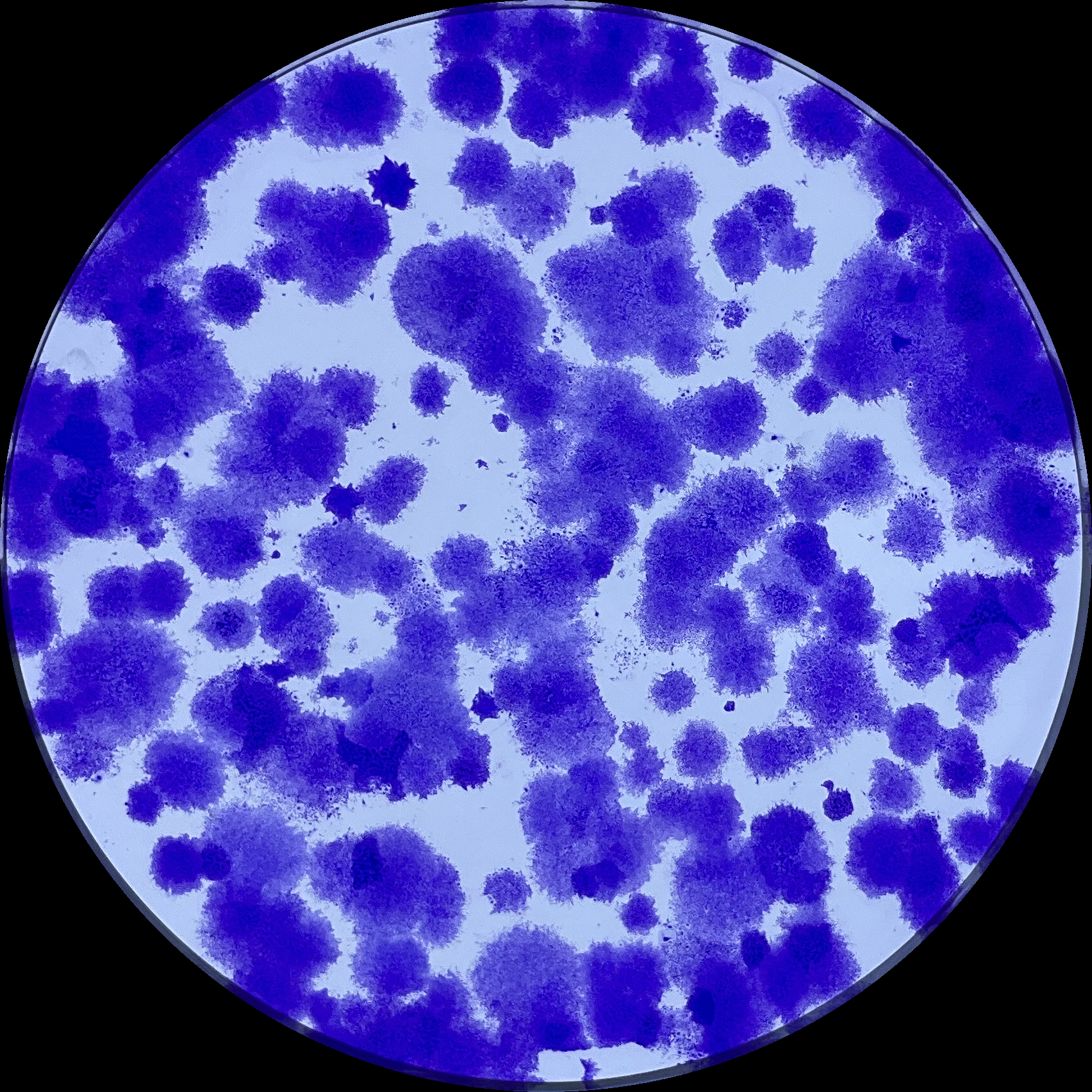

Supplement: Supplementary file 7 — Source data Fig. 2 [file 44321_2025_287_MOESM7_ESM.zip › Figure 2 /2H/Colony Formation Image U251 WT.tif]

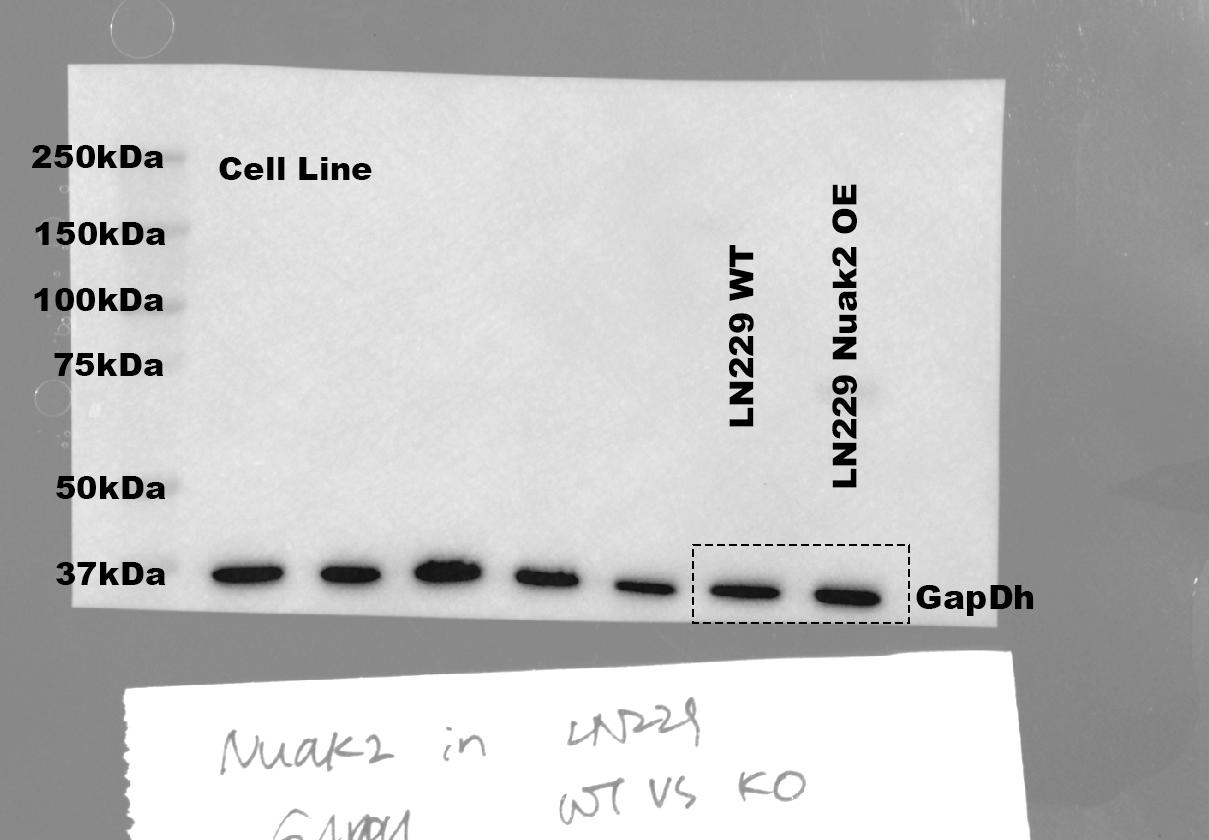

Supplement: Supplementary file 8 — Source data Fig. 3 [file 44321_2025_287_MOESM8_ESM.zip › Figure 3 /3A/Western GapDh and LN229.tif]

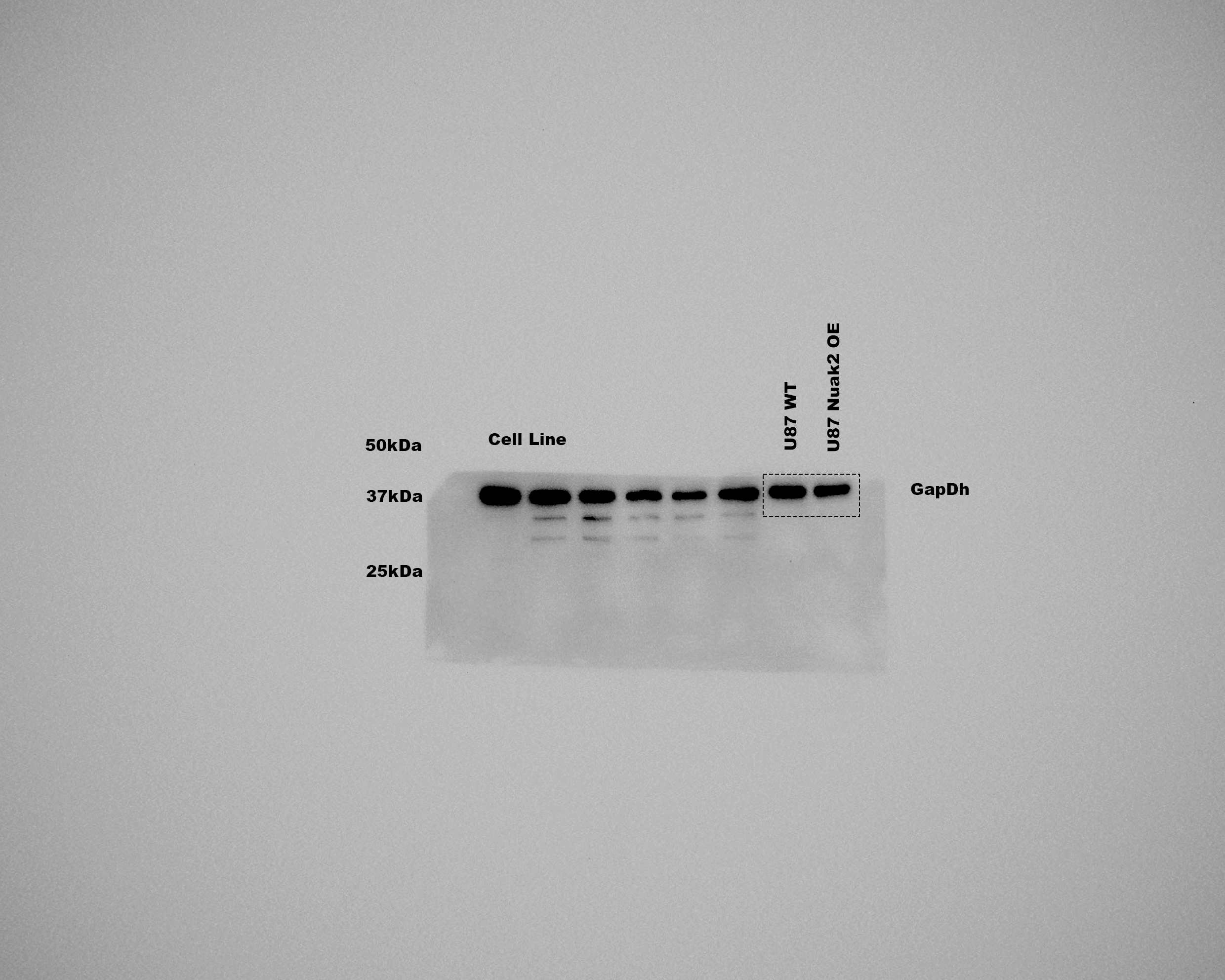

Supplement: Supplementary file 8 — Source data Fig. 3 [file 44321_2025_287_MOESM8_ESM.zip › Figure 3 /3A/Western GapDh and U87.tif]

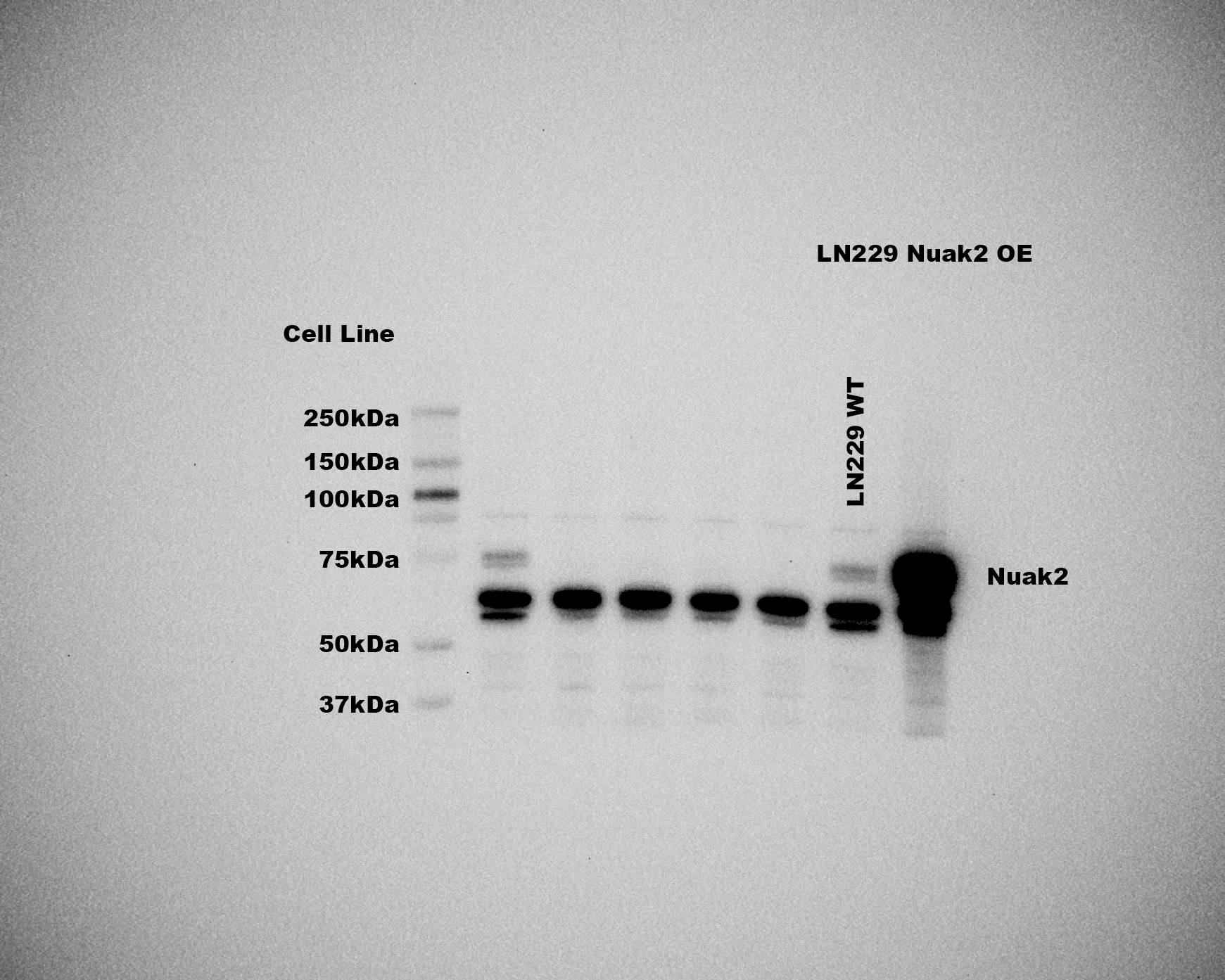

Supplement: Supplementary file 8 — Source data Fig. 3 [file 44321_2025_287_MOESM8_ESM.zip › Figure 3 /3A/Western Nuak2 and LN229.tif]

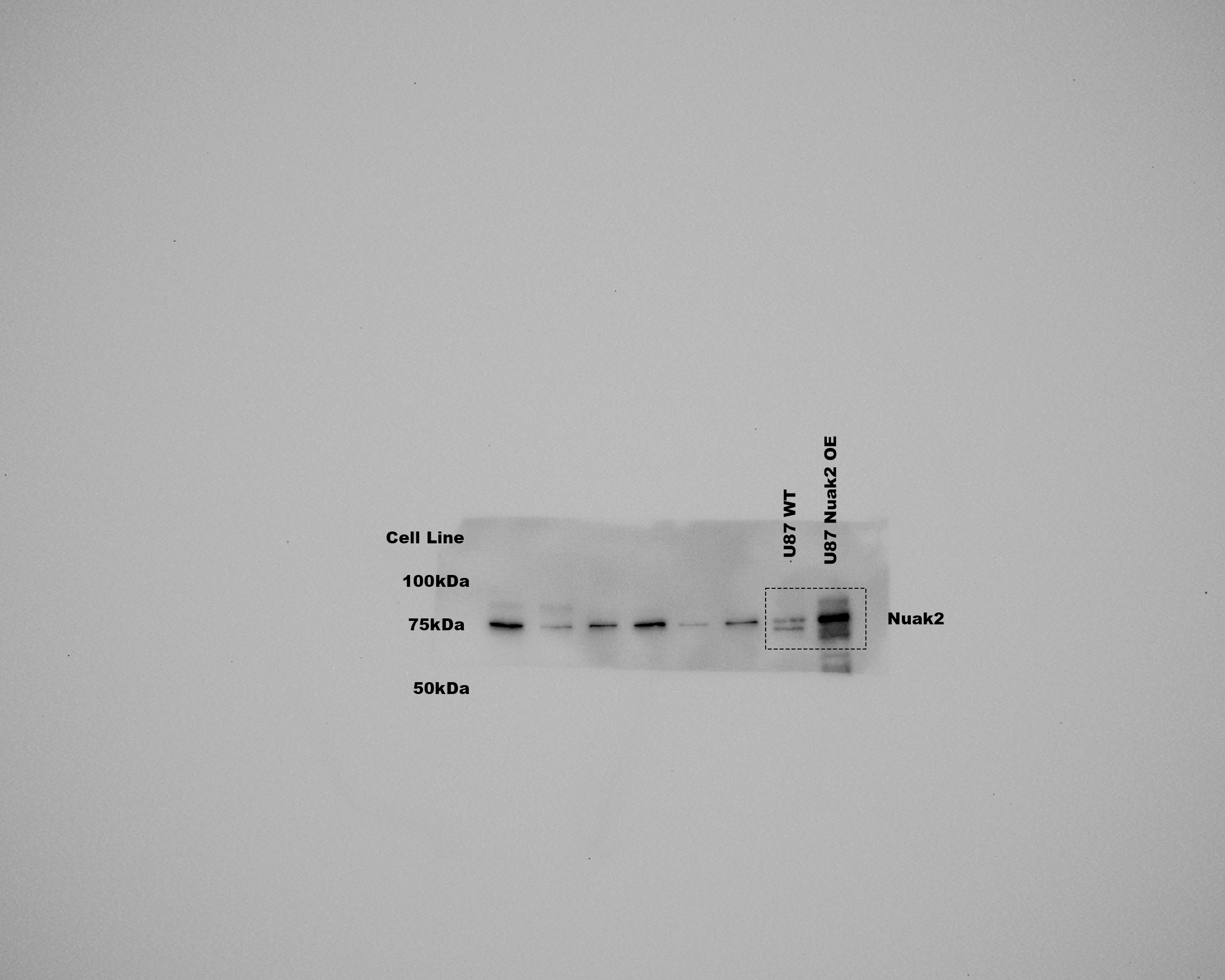

Supplement: Supplementary file 8 — Source data Fig. 3 [file 44321_2025_287_MOESM8_ESM.zip › Figure 3 /3A/Western Nuak2 and U87.tif]

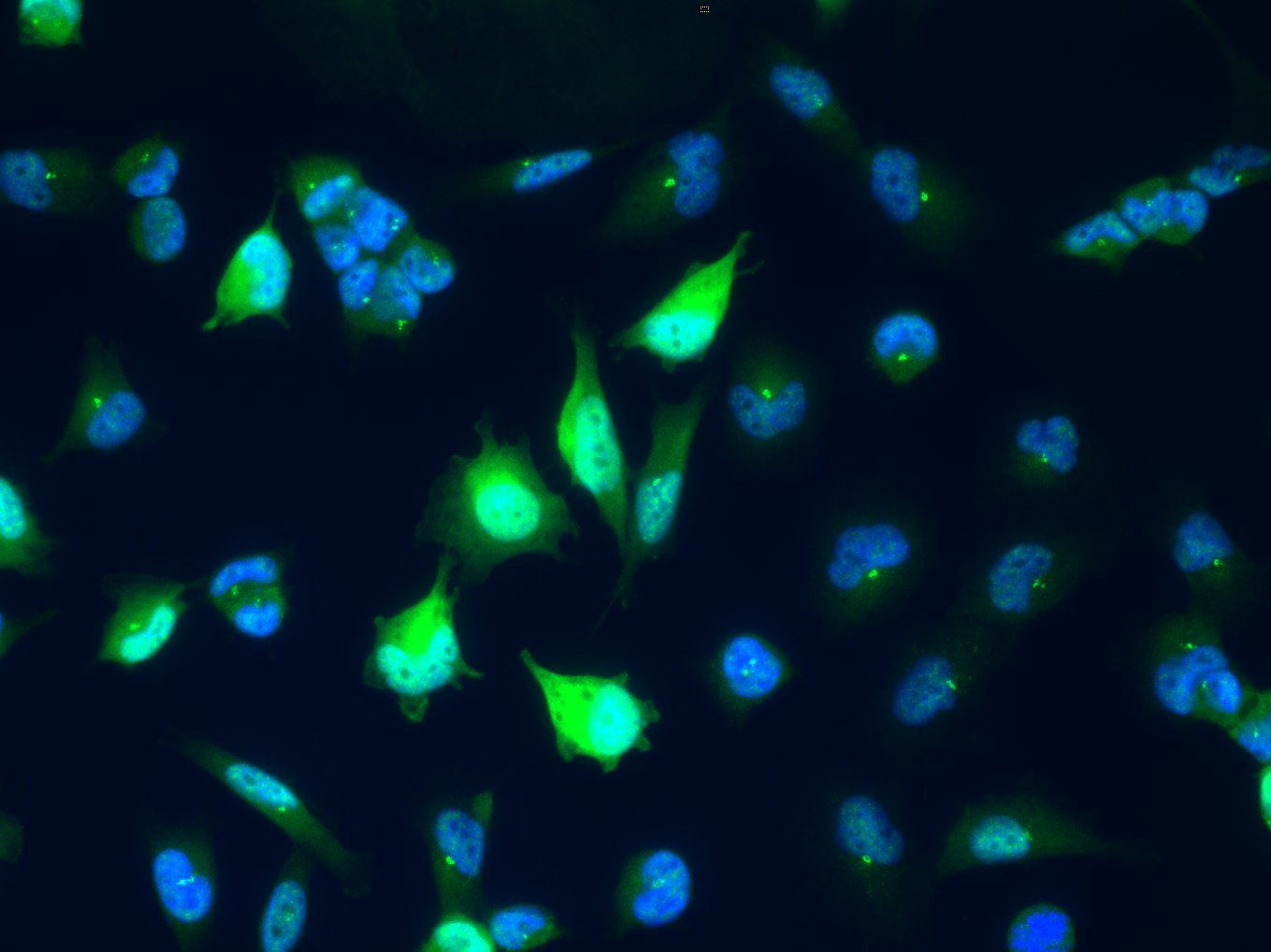

Supplement: Supplementary file 8 — Source data Fig. 3 [file 44321_2025_287_MOESM8_ESM.zip › Figure 3 /3B/ICC Image LN229 NUAK2 OE Merge.tif]

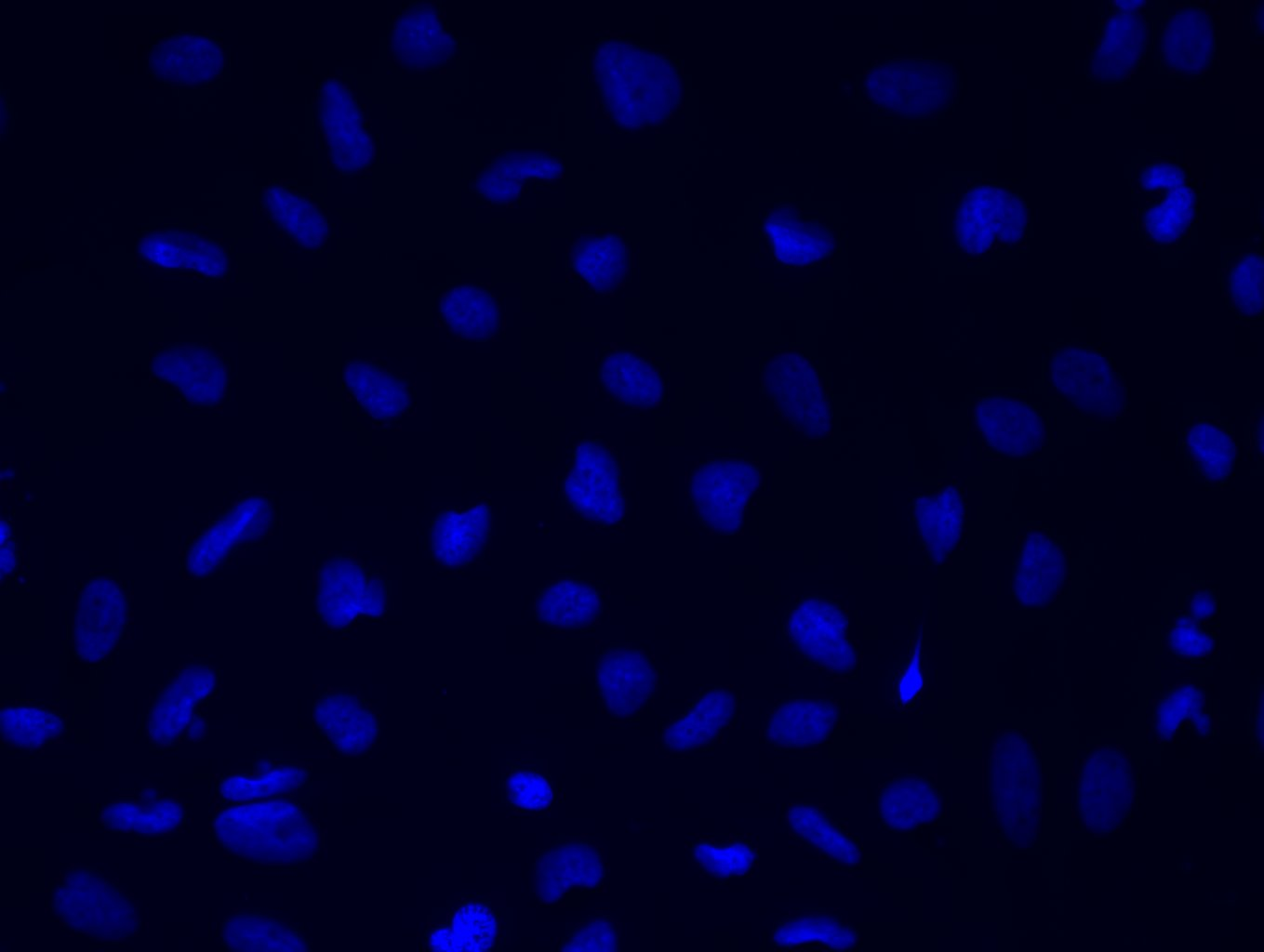

Supplement: Supplementary file 8 — Source data Fig. 3 [file 44321_2025_287_MOESM8_ESM.zip › Figure 3 /3B/ICC Image LN229 WT DAPI.tif]

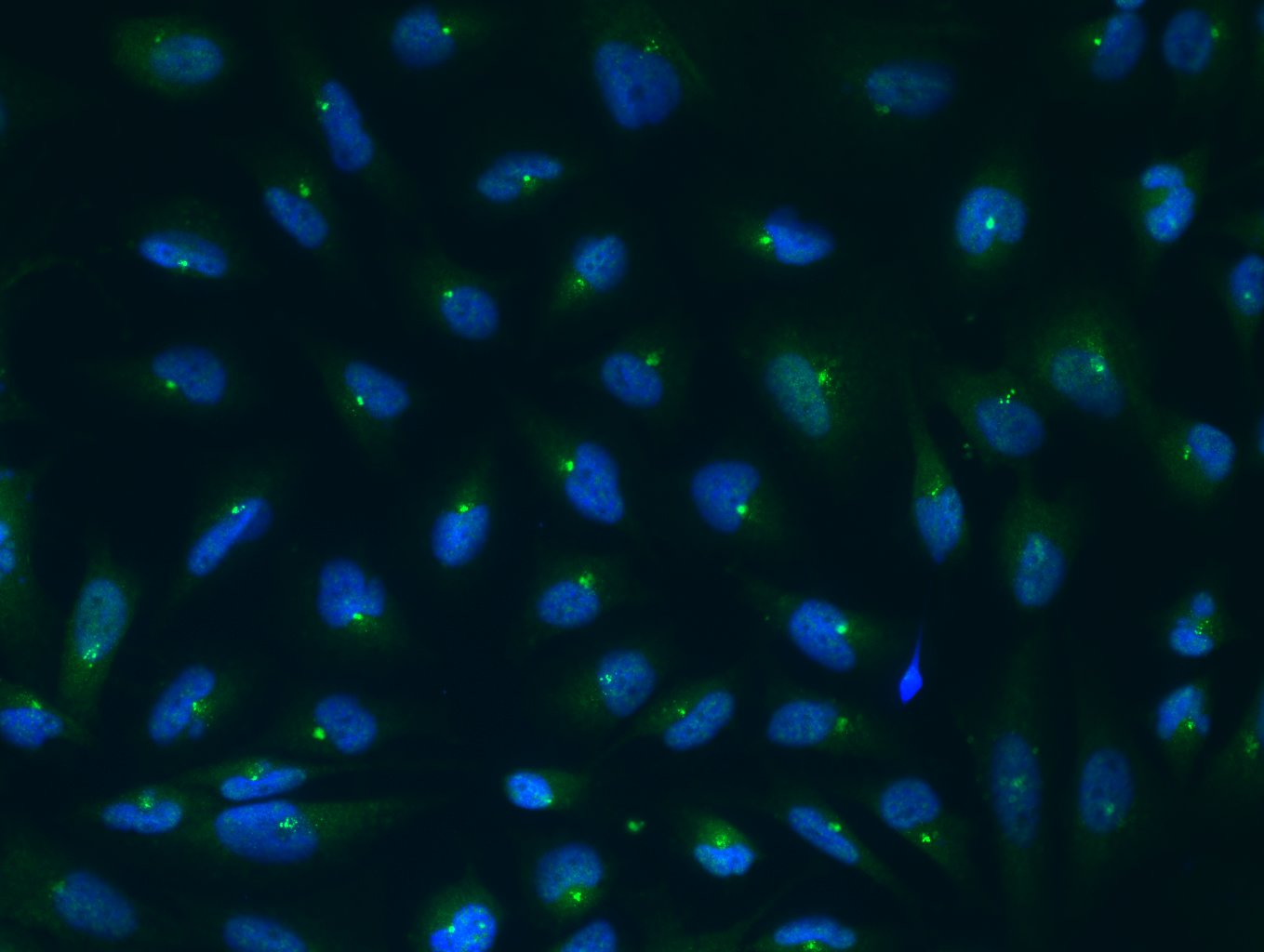

Supplement: Supplementary file 8 — Source data Fig. 3 [file 44321_2025_287_MOESM8_ESM.zip › Figure 3 /3B/ICC Image LN229 WT Merge.tif]

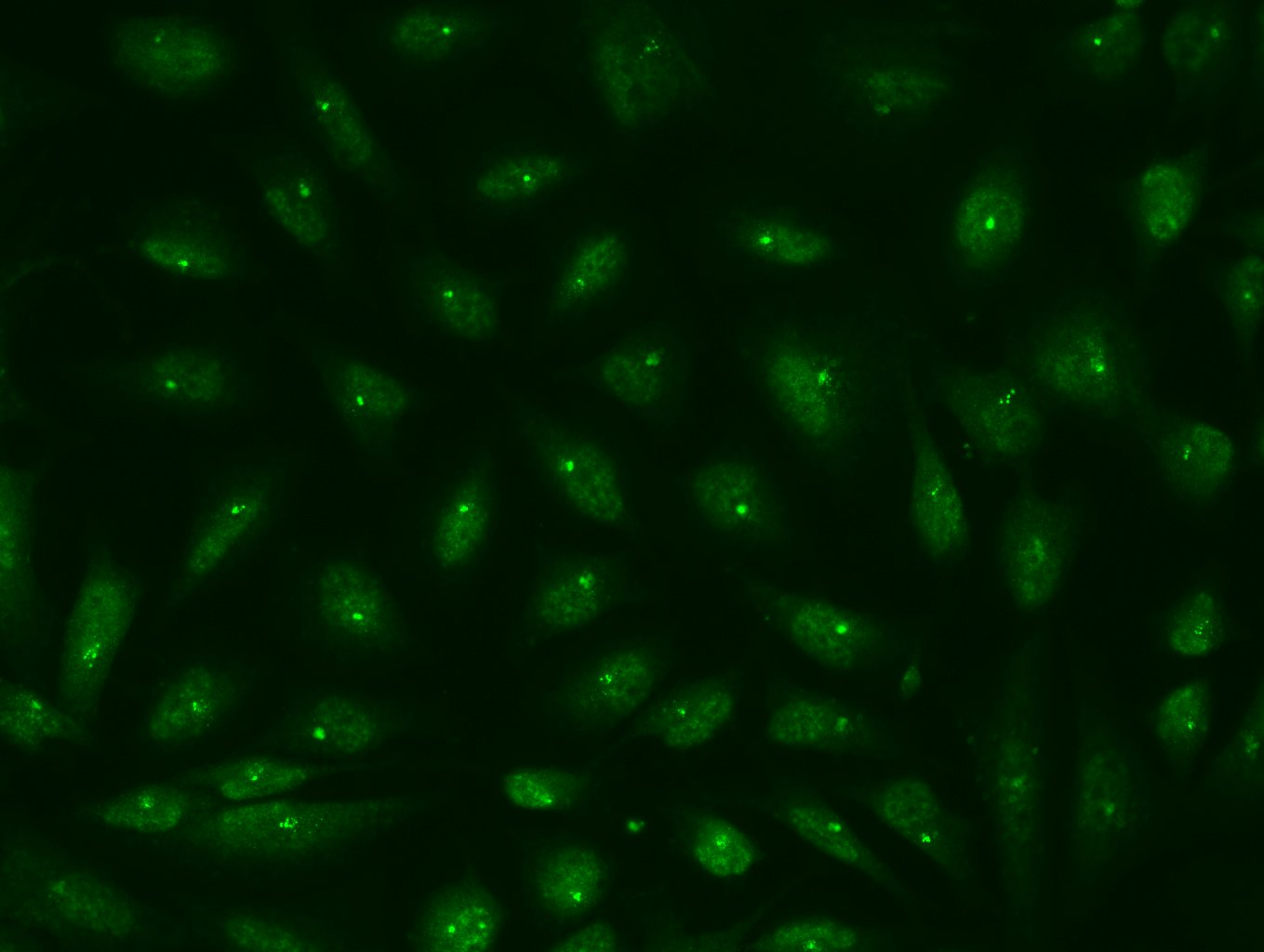

Supplement: Supplementary file 8 — Source data Fig. 3 [file 44321_2025_287_MOESM8_ESM.zip › Figure 3 /3B/ICC Image LN229 WT NUAK2 488.tif]

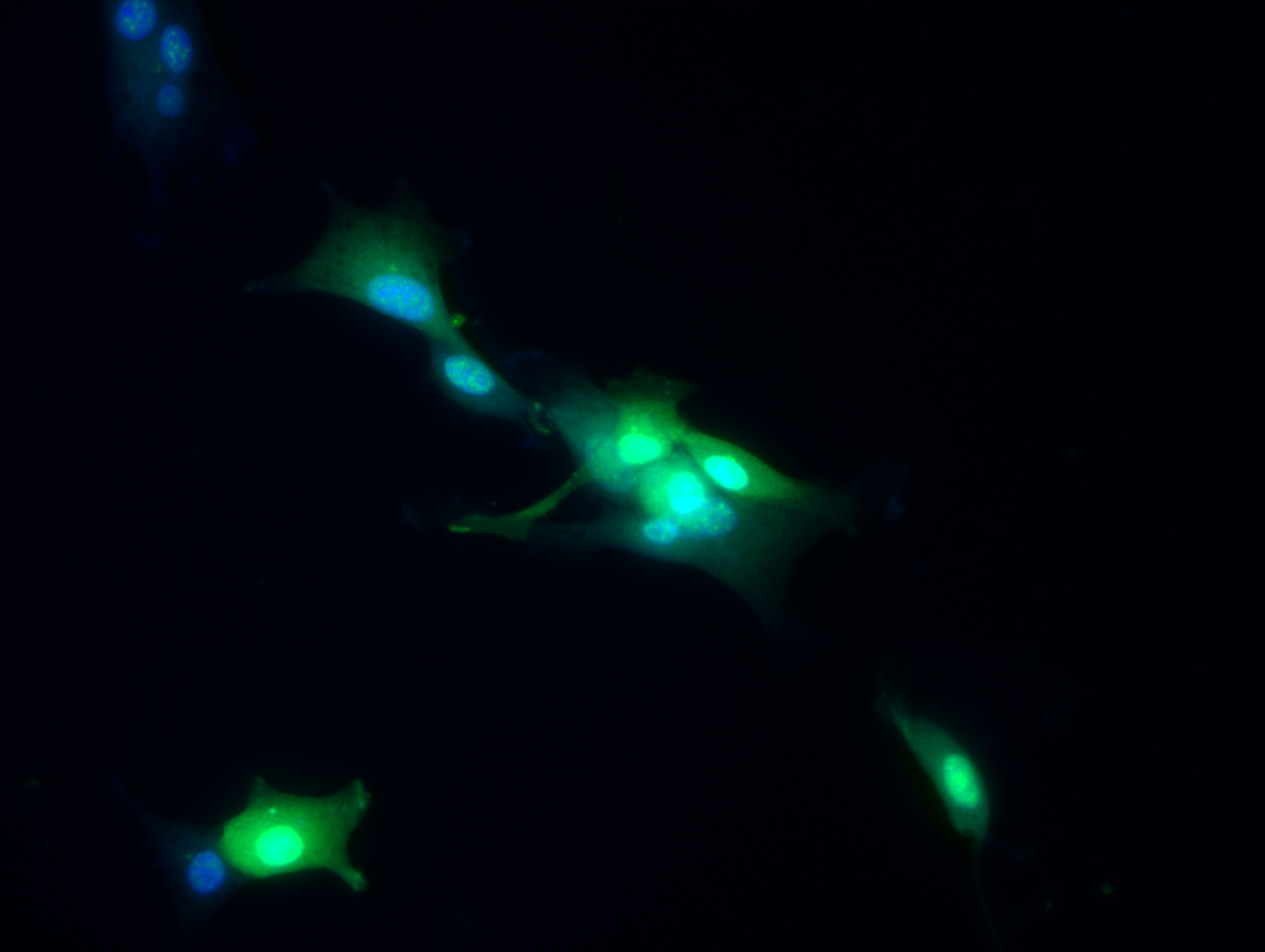

Supplement: Supplementary file 8 — Source data Fig. 3 [file 44321_2025_287_MOESM8_ESM.zip › Figure 3 /3B/ICC Image U87 NUAK2 OE Merge.tif]

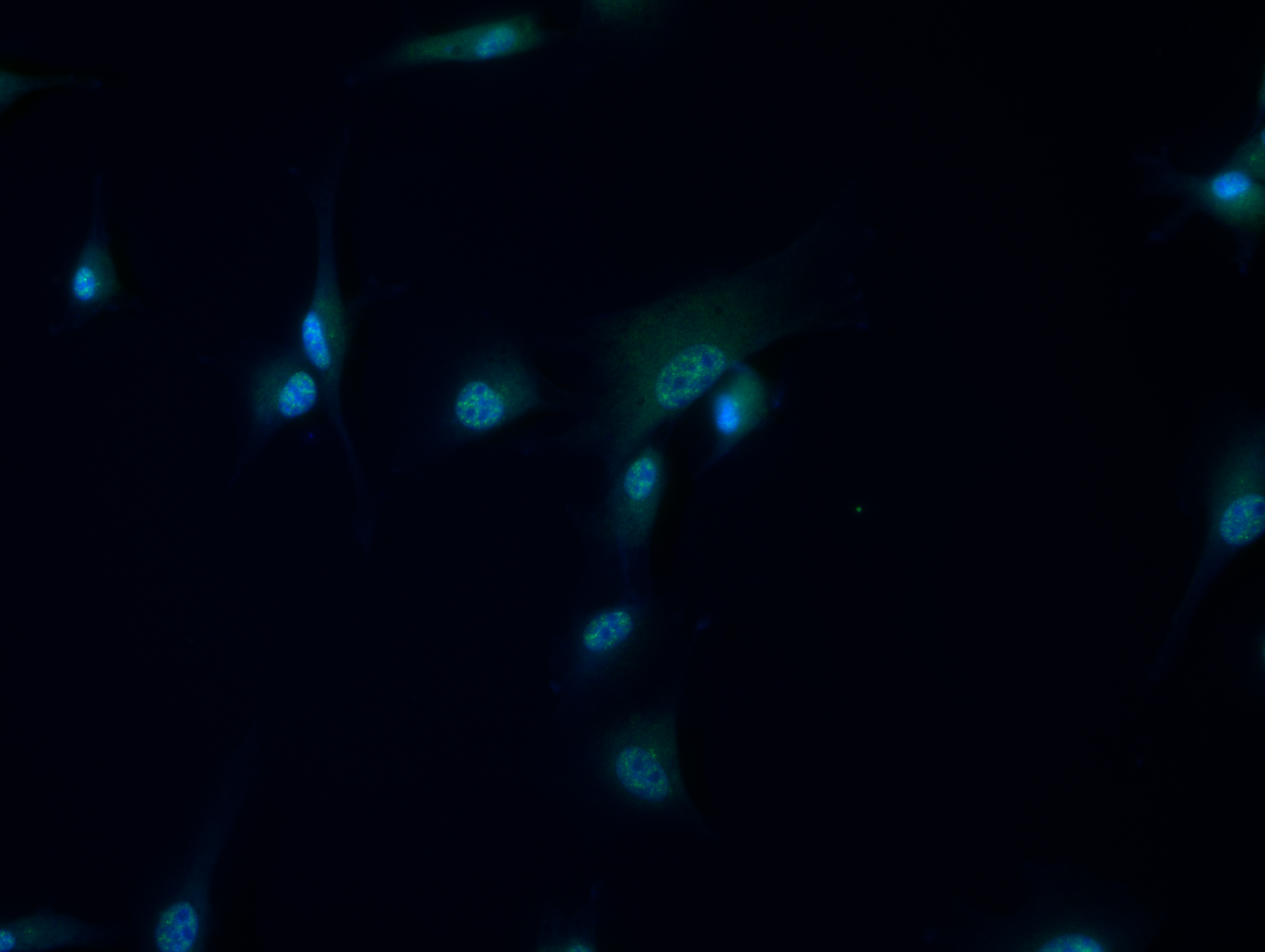

Supplement: Supplementary file 8 — Source data Fig. 3 [file 44321_2025_287_MOESM8_ESM.zip › Figure 3 /3B/ICC Image U87 WT Merge.tif]

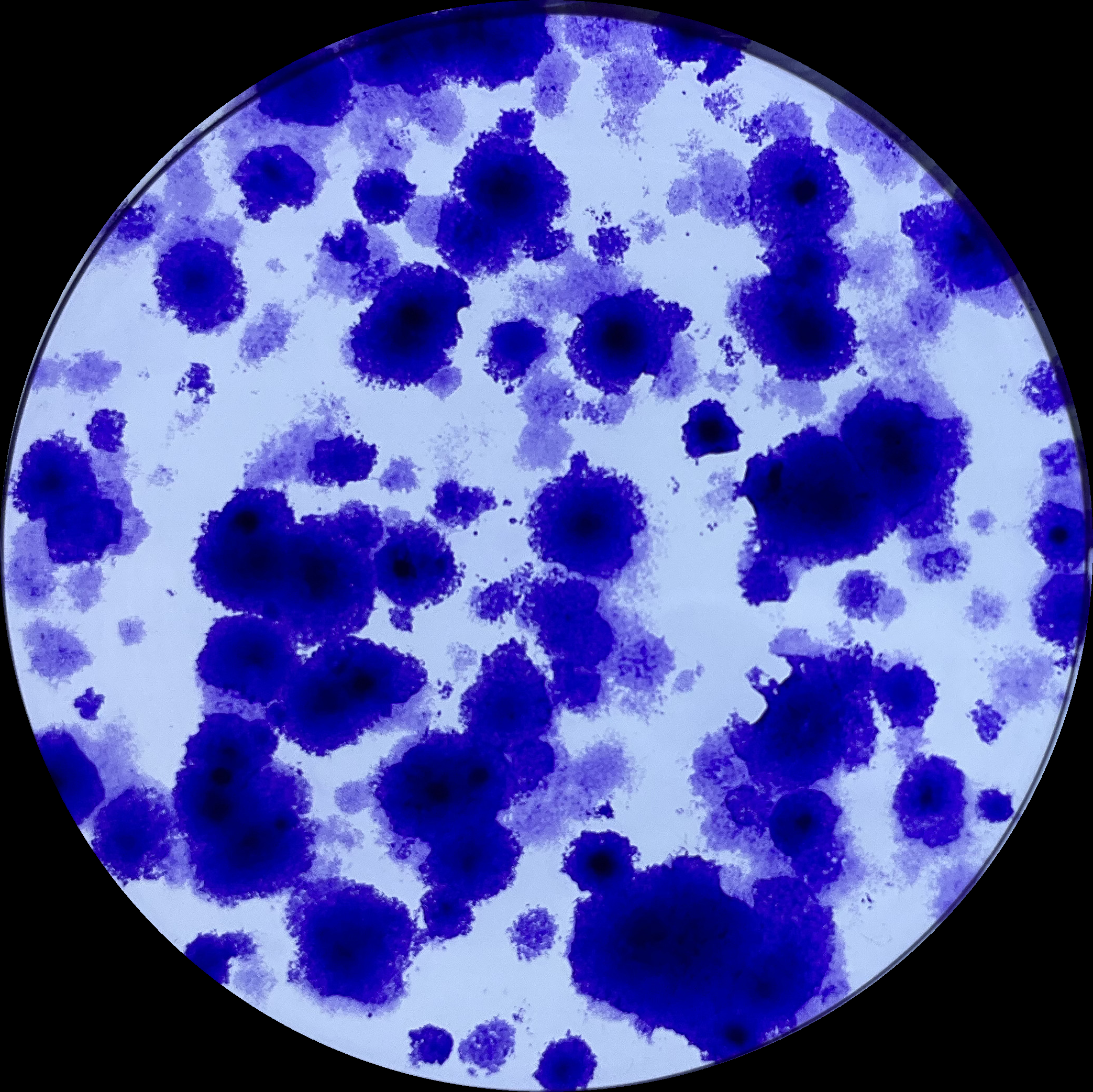

Supplement: Supplementary file 8 — Source data Fig. 3 [file 44321_2025_287_MOESM8_ESM.zip › Figure 3 /3D/Colony Formation Image LN229 NUAK2 OE.tif]

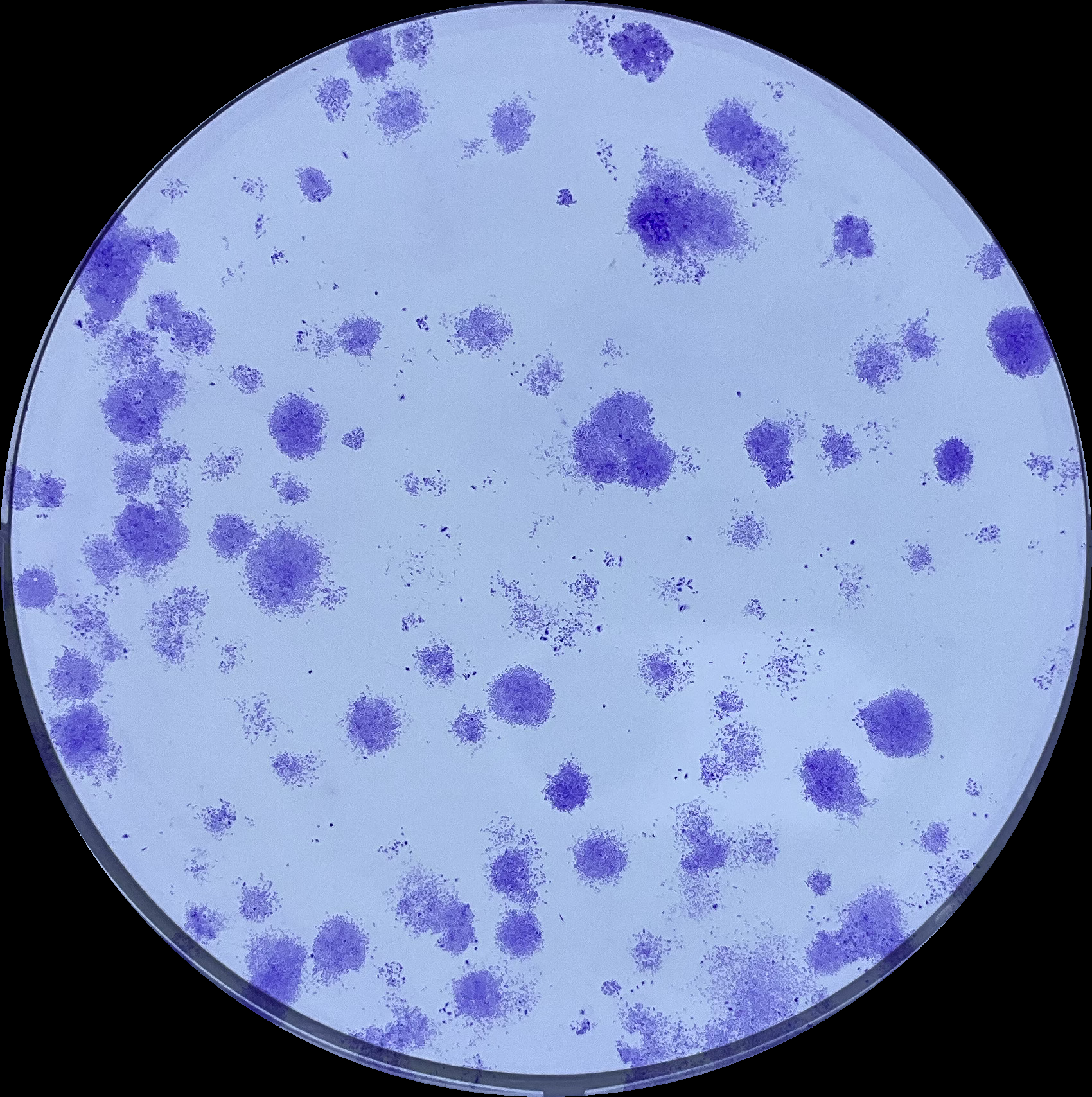

Supplement: Supplementary file 8 — Source data Fig. 3 [file 44321_2025_287_MOESM8_ESM.zip › Figure 3 /3D/Colony Formation Image LN229 WT.tif]

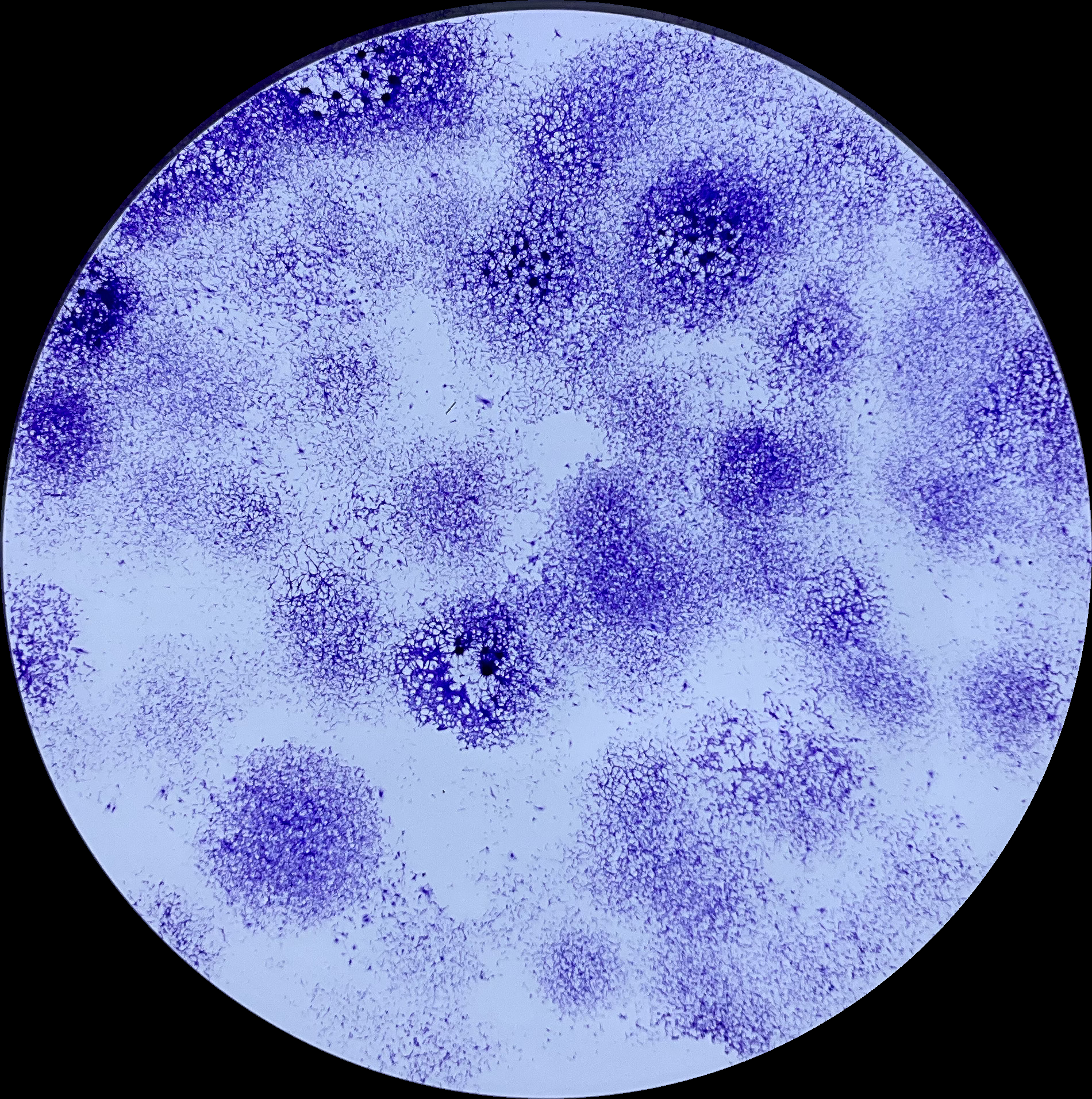

Supplement: Supplementary file 8 — Source data Fig. 3 [file 44321_2025_287_MOESM8_ESM.zip › Figure 3 /3D/Colony Formation Image U87 NUAK2 OE.tif]

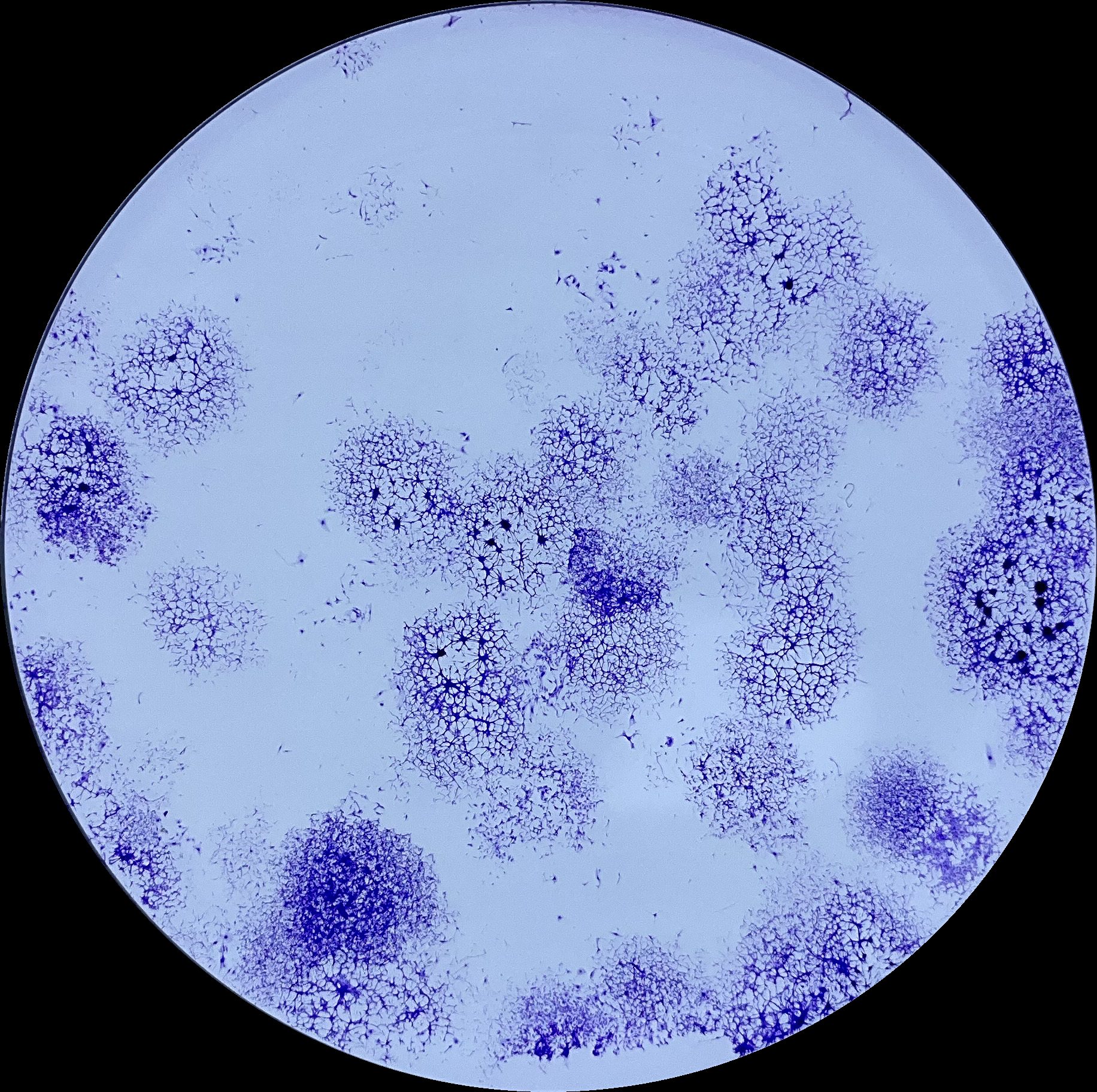

Supplement: Supplementary file 8 — Source data Fig. 3 [file 44321_2025_287_MOESM8_ESM.zip › Figure 3 /3D/Colony Formation Image U87 WT.tif]

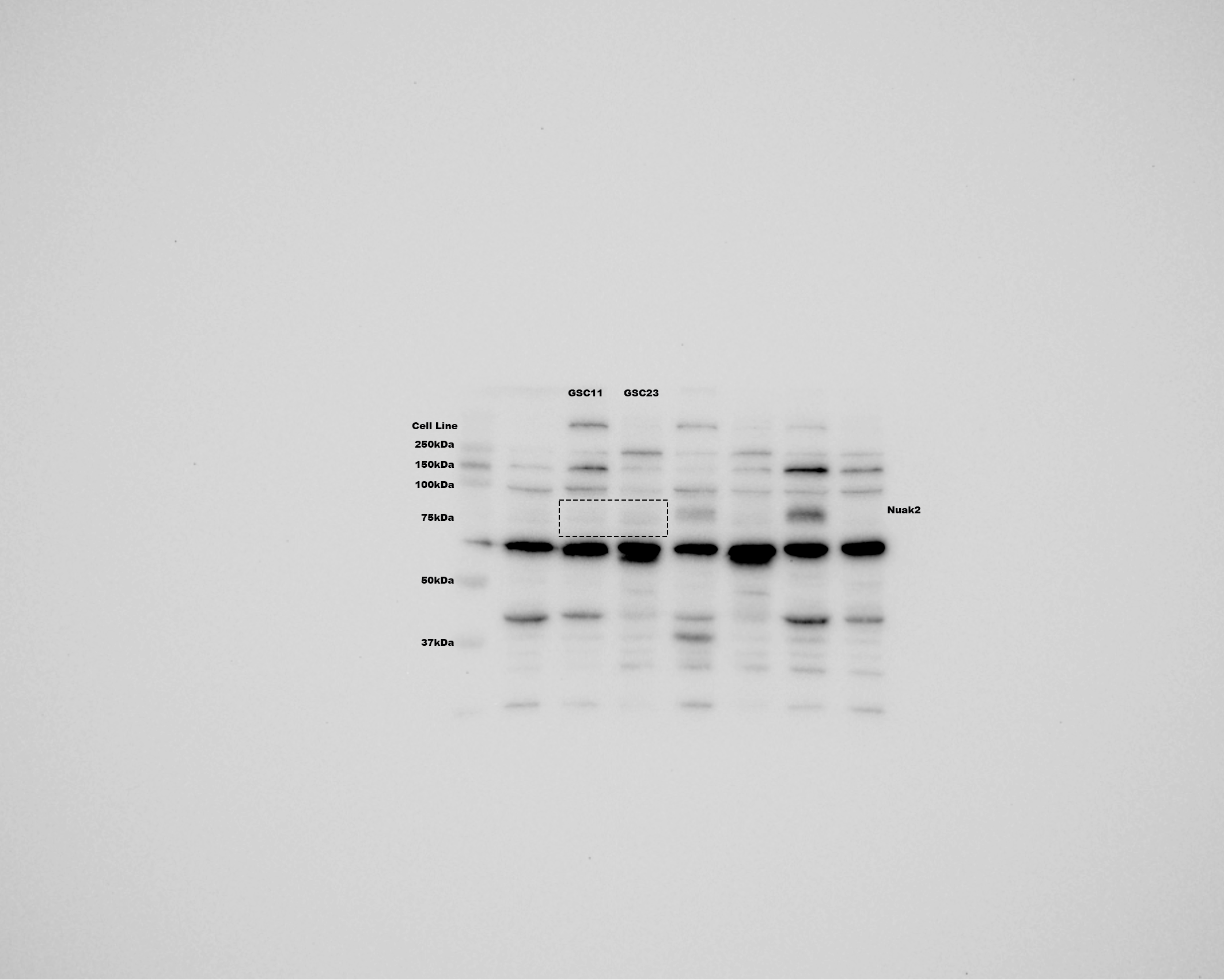

Supplement: Supplementary file 8 — Source data Fig. 3 [file 44321_2025_287_MOESM8_ESM.zip › Figure 3 /3E/Western Nuak2 GSC Annotated.tif]

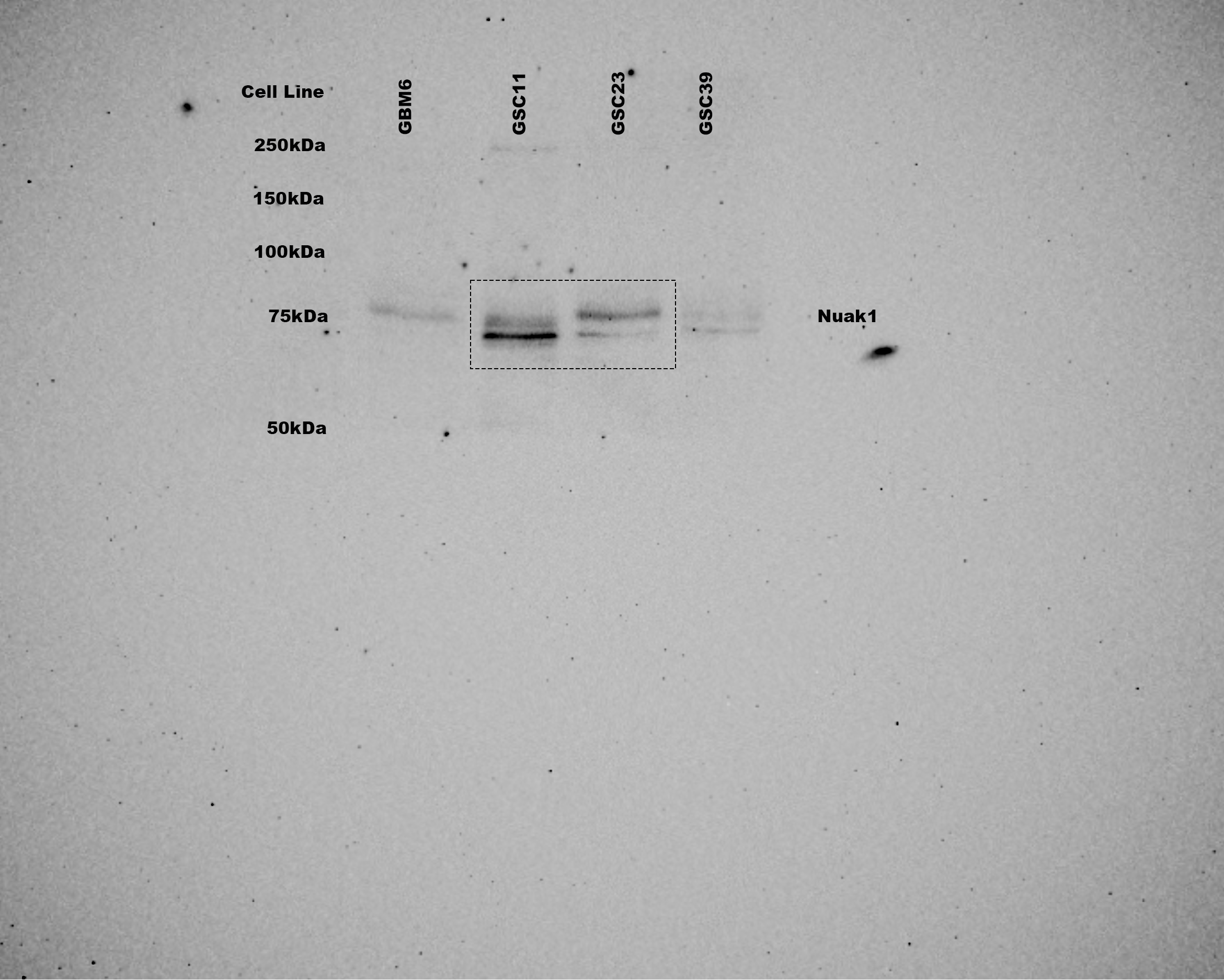

Supplement: Supplementary file 8 — Source data Fig. 3 [file 44321_2025_287_MOESM8_ESM.zip › Figure 3 /3E/Western Nuak1 GSC11 23 annotated.tif]

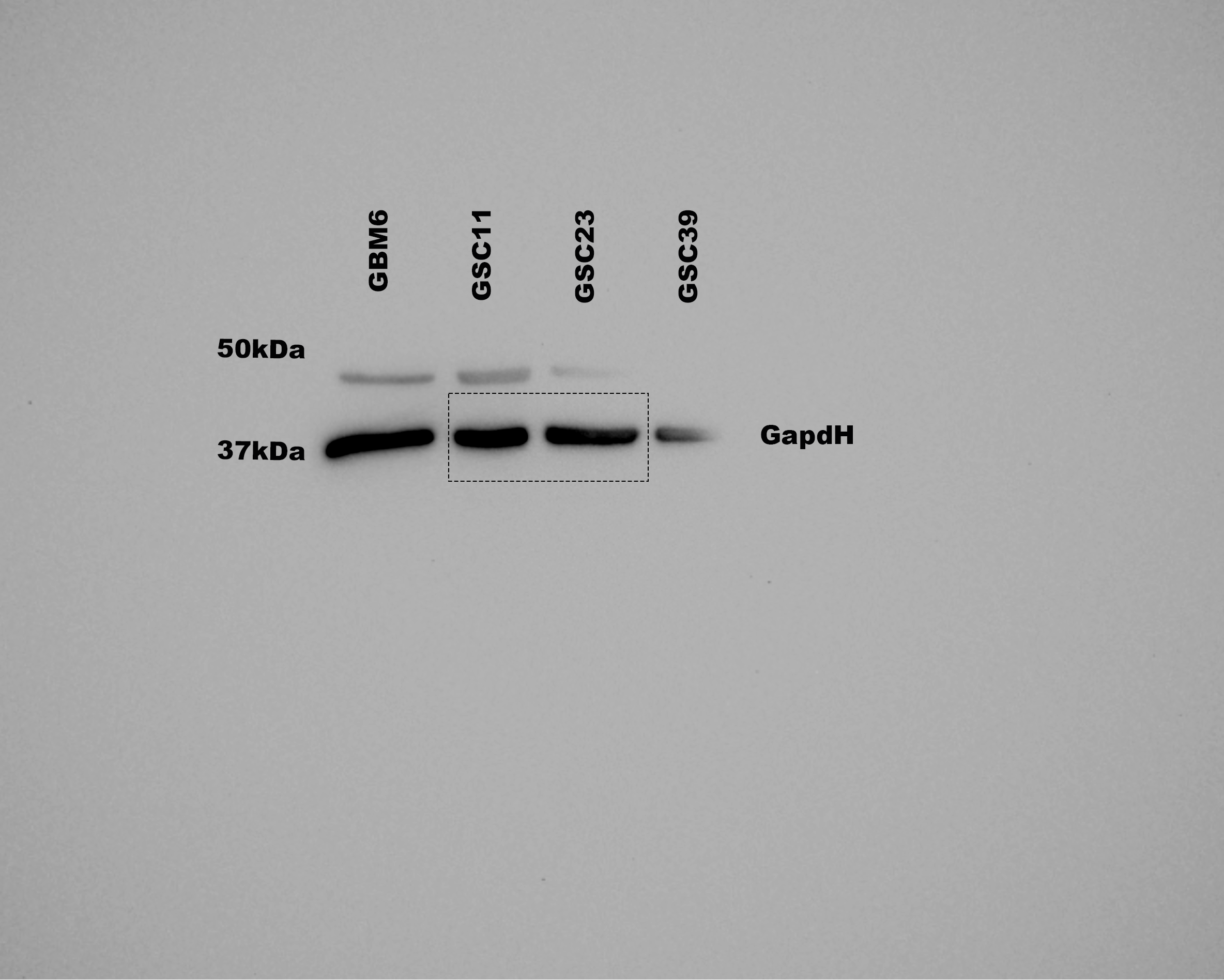

Supplement: Supplementary file 8 — Source data Fig. 3 [file 44321_2025_287_MOESM8_ESM.zip › Figure 3 /3E/Western GapDh for Nuak1 GSC11 23 annotated.tif]

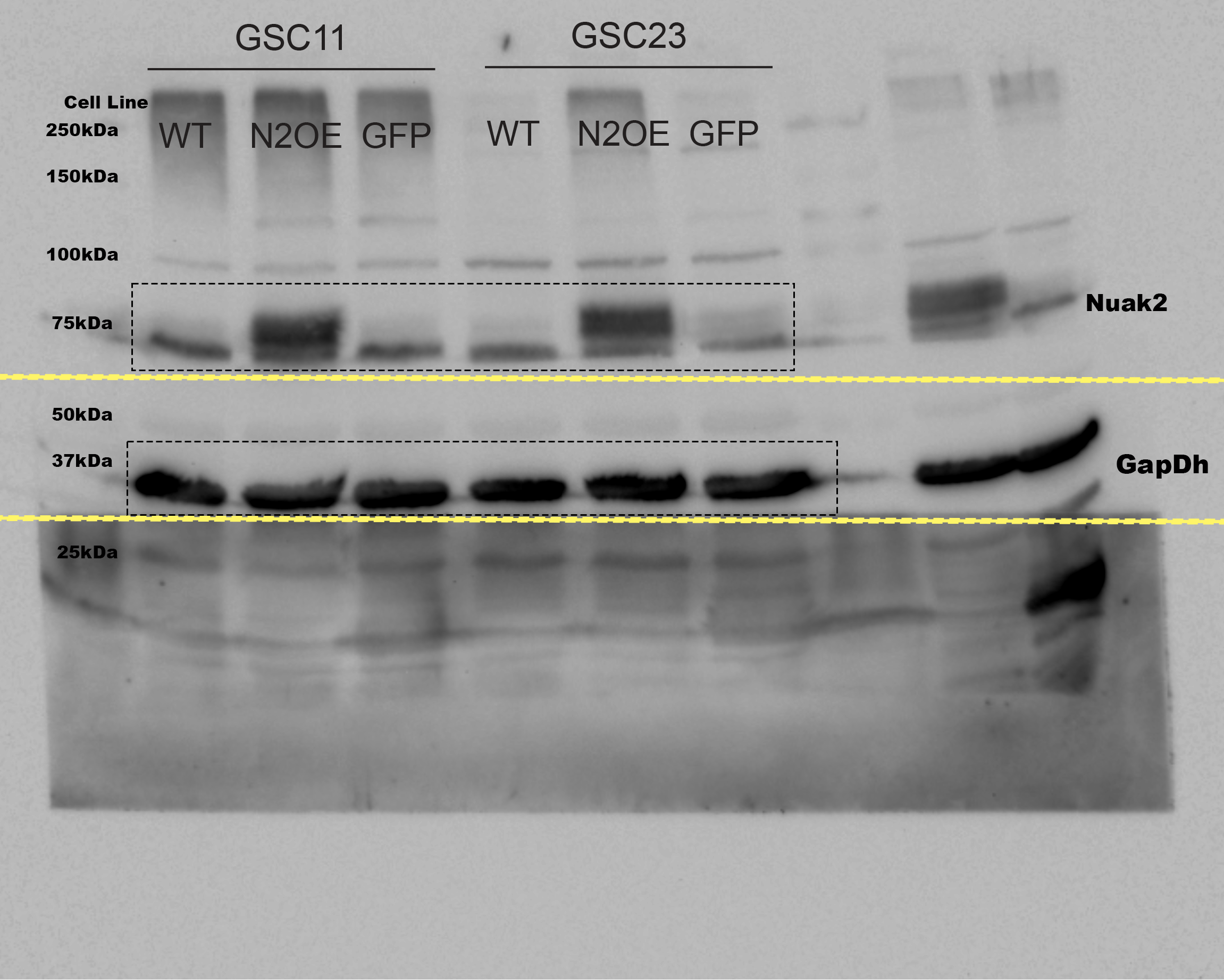

Supplement: Supplementary file 8 — Source data Fig. 3 [file 44321_2025_287_MOESM8_ESM.zip › Figure 3 /3F/Western Nuak2 and GapDh.tif]

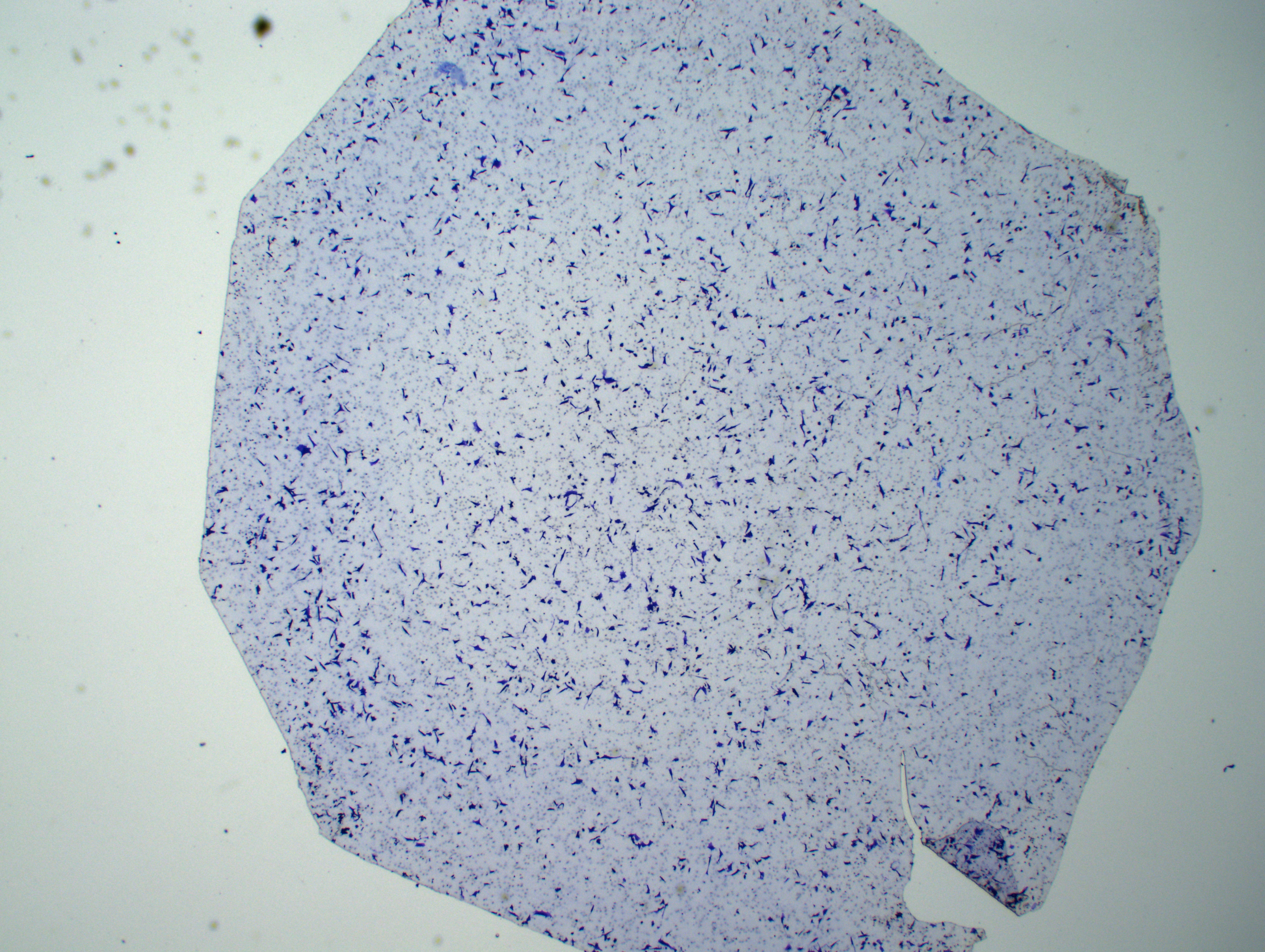

Supplement: Supplementary file 8 — Source data Fig. 3 [file 44321_2025_287_MOESM8_ESM.zip › Figure 3 /3I/Transwell GSC11 GFP.tif]

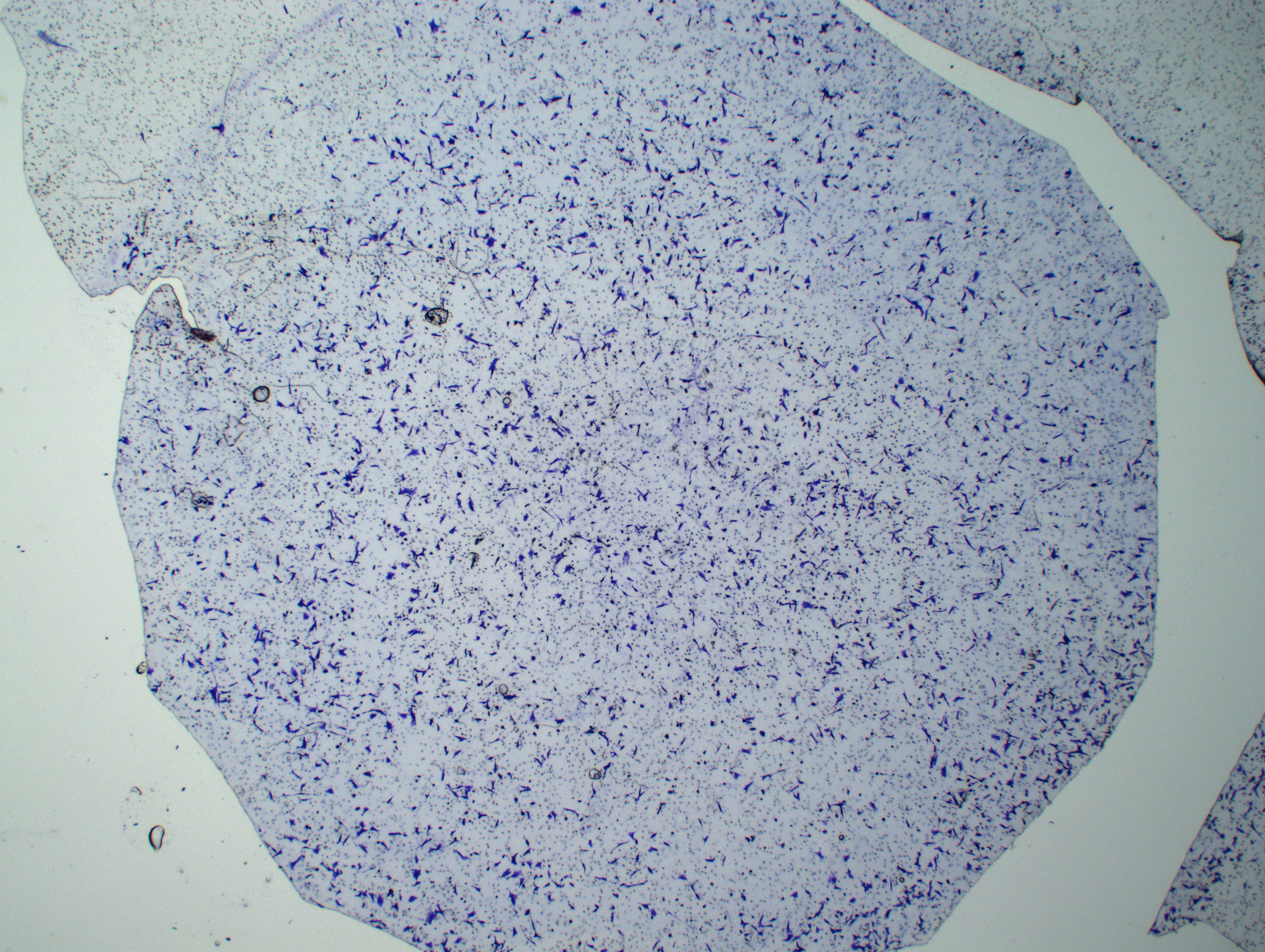

Supplement: Supplementary file 8 — Source data Fig. 3 [file 44321_2025_287_MOESM8_ESM.zip › Figure 3 /3I/Transwell GSC11 NUAK2 OE.tif]

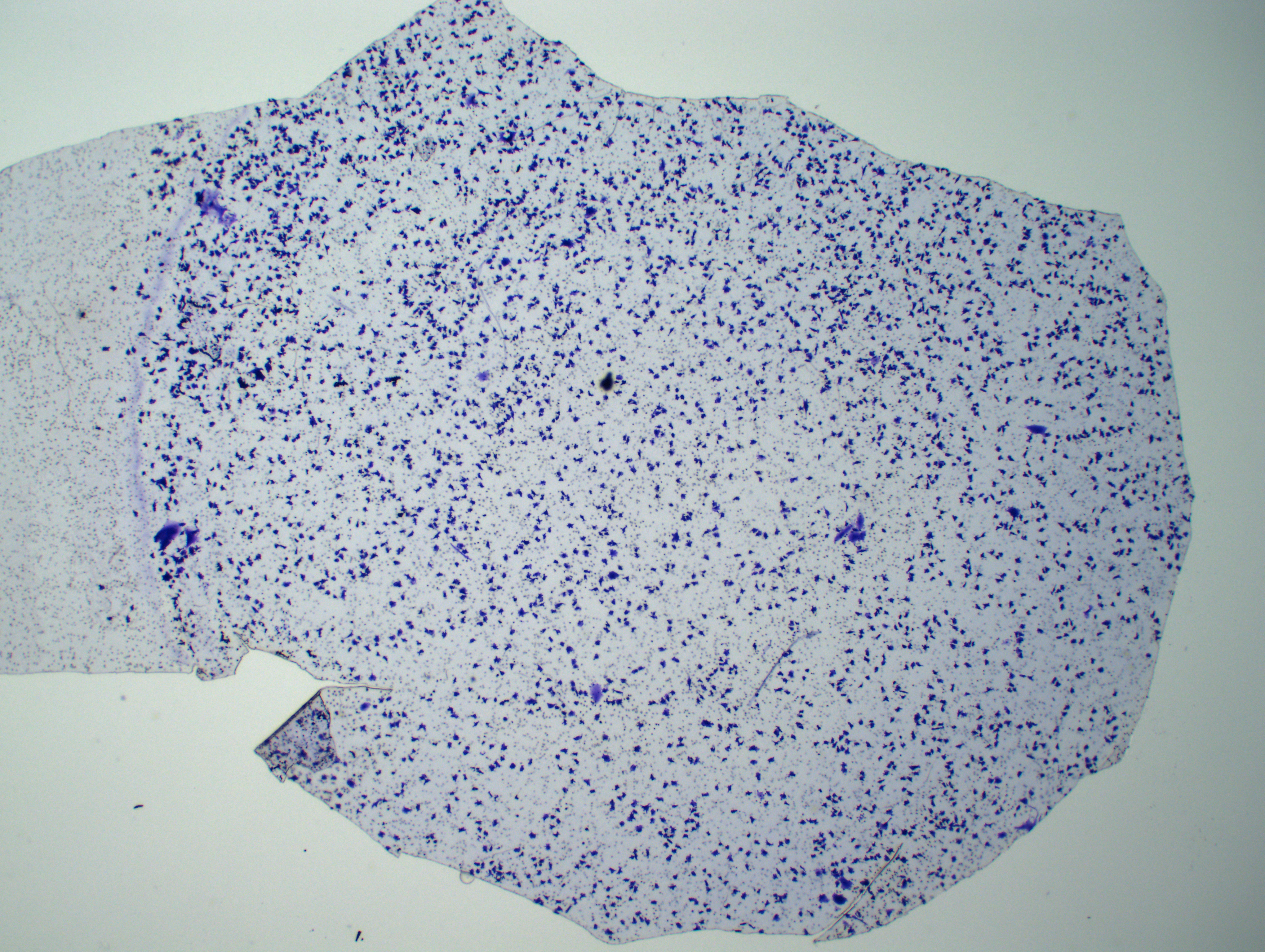

Supplement: Supplementary file 8 — Source data Fig. 3 [file 44321_2025_287_MOESM8_ESM.zip › Figure 3 /3I/Transwell GSC23 GFP.tif]

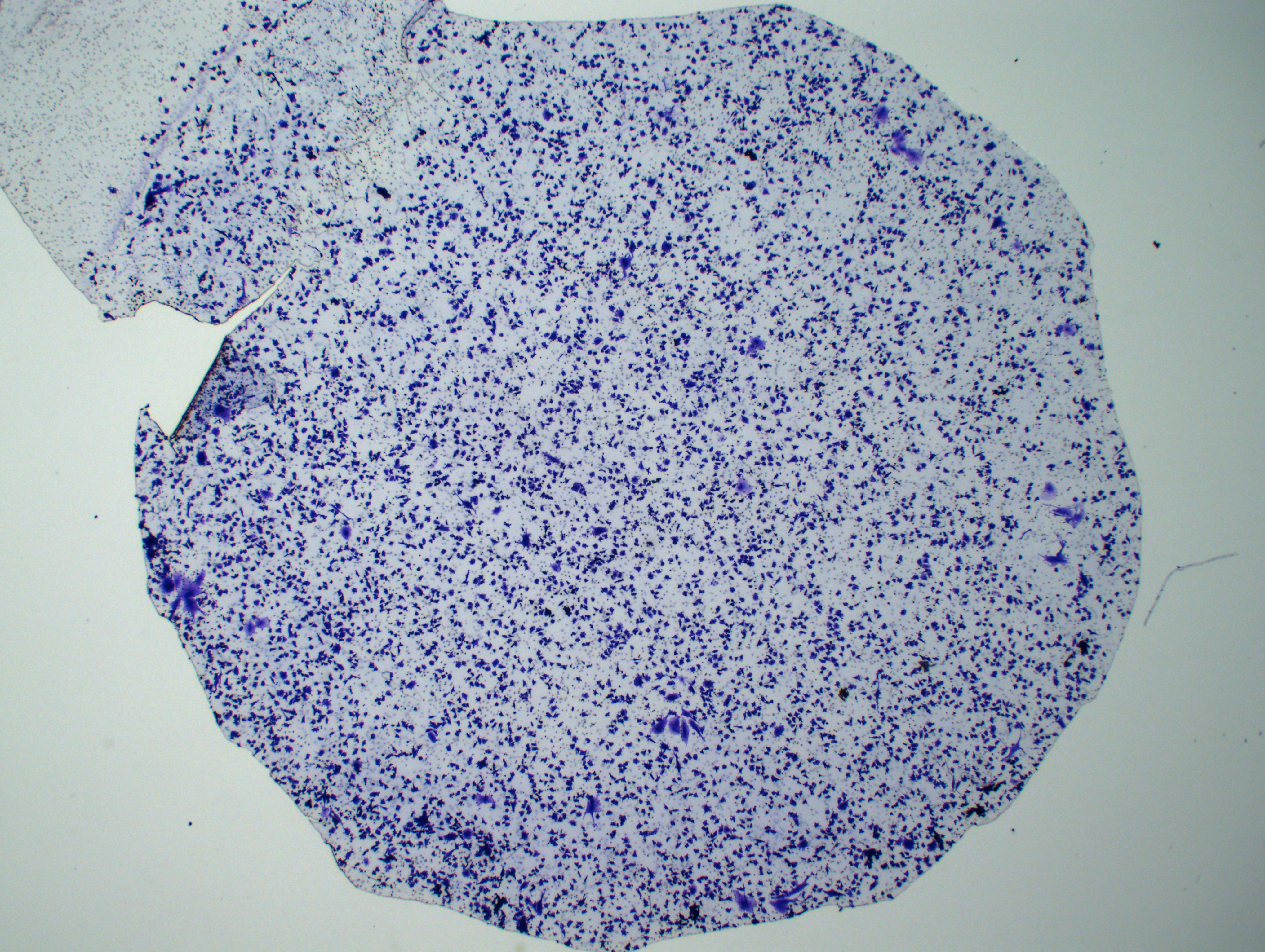

Supplement: Supplementary file 8 — Source data Fig. 3 [file 44321_2025_287_MOESM8_ESM.zip › Figure 3 /3I/Transwell GSC23 NUAK2 OE.tif]

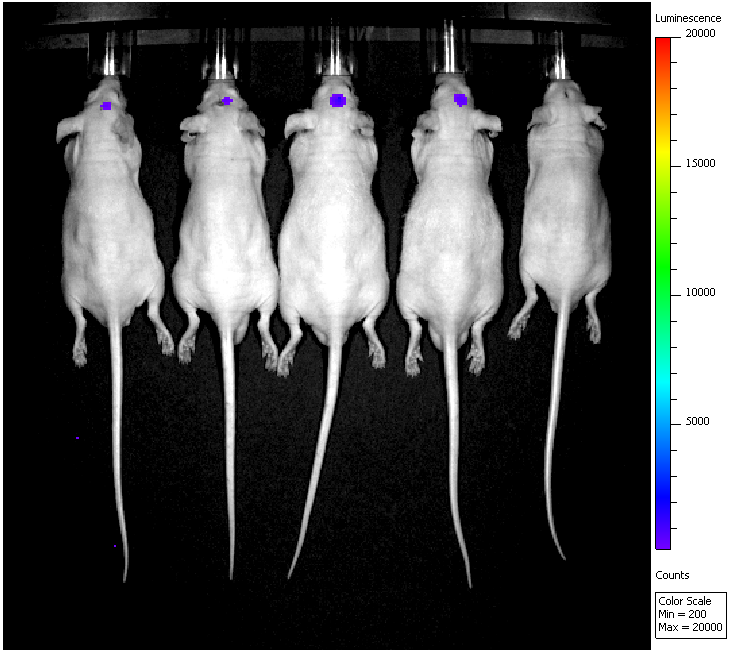

Supplement: Supplementary file 9 — Source data Fig. 4 [file 44321_2025_287_MOESM9_ESM.zip › Figure 4 /4A/IVIS Image KO_group3_7dpi.png]

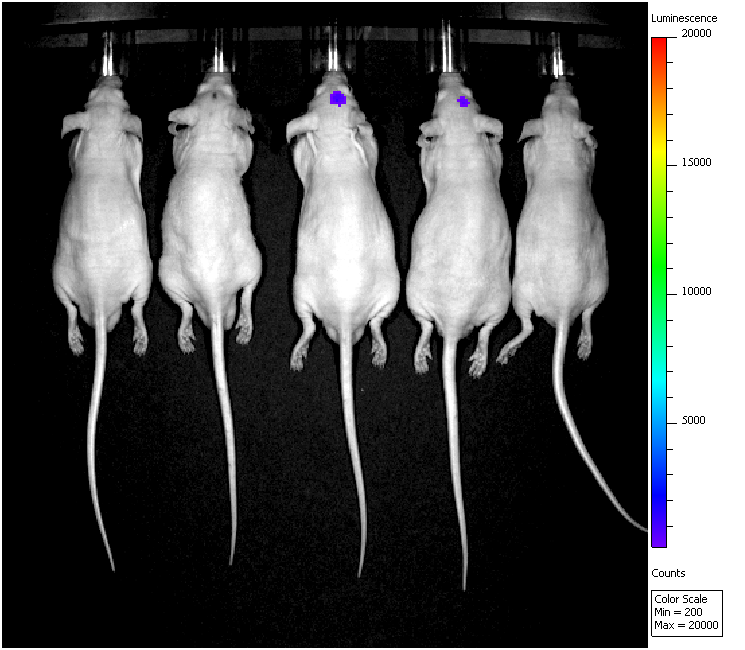

Supplement: Supplementary file 9 — Source data Fig. 4 [file 44321_2025_287_MOESM9_ESM.zip › Figure 4 /4A/IVIS Image KO_group3_14dpi.png]

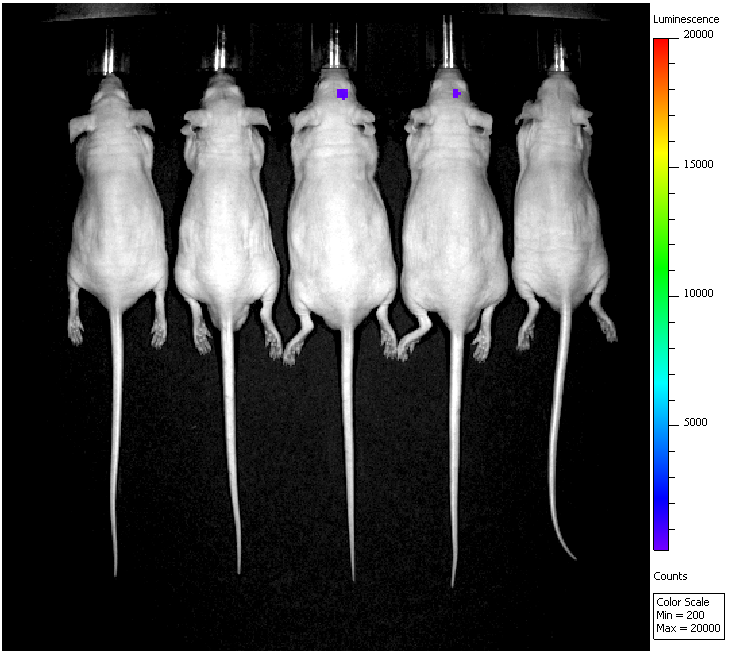

Supplement: Supplementary file 9 — Source data Fig. 4 [file 44321_2025_287_MOESM9_ESM.zip › Figure 4 /4A/IVIS Image KO_group3_21dpi.png]

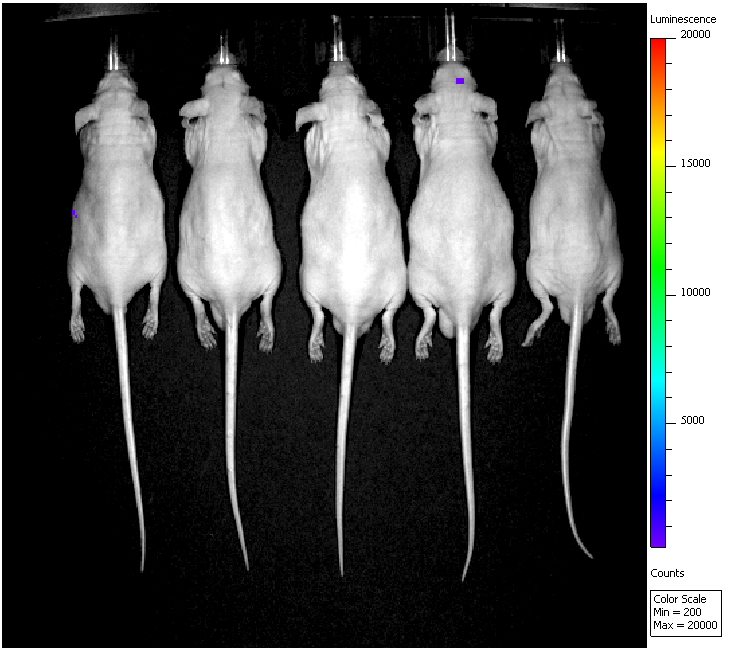

Supplement: Supplementary file 9 — Source data Fig. 4 [file 44321_2025_287_MOESM9_ESM.zip › Figure 4 /4A/IVIS Image KO_group3_28dpi.png]

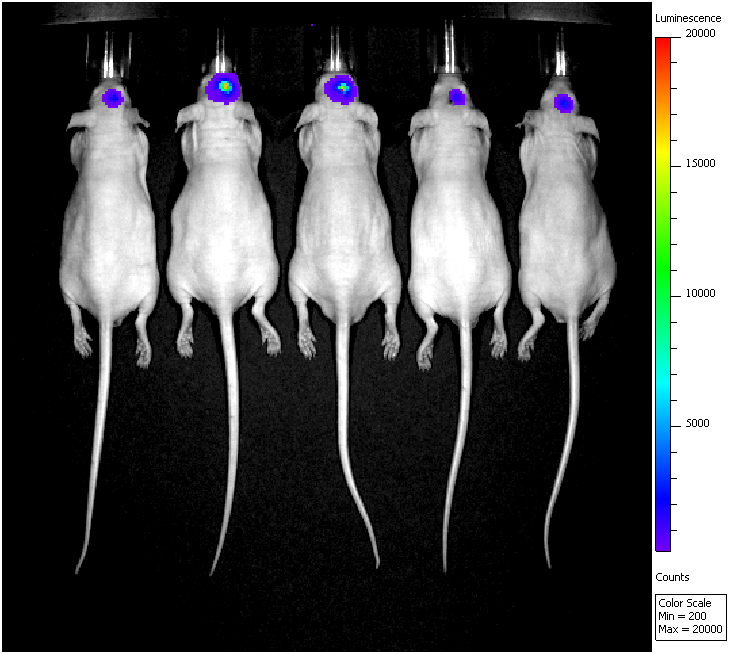

Supplement: Supplementary file 9 — Source data Fig. 4 [file 44321_2025_287_MOESM9_ESM.zip › Figure 4 /4A/IVIS Image WT_group3_7dpi.png]

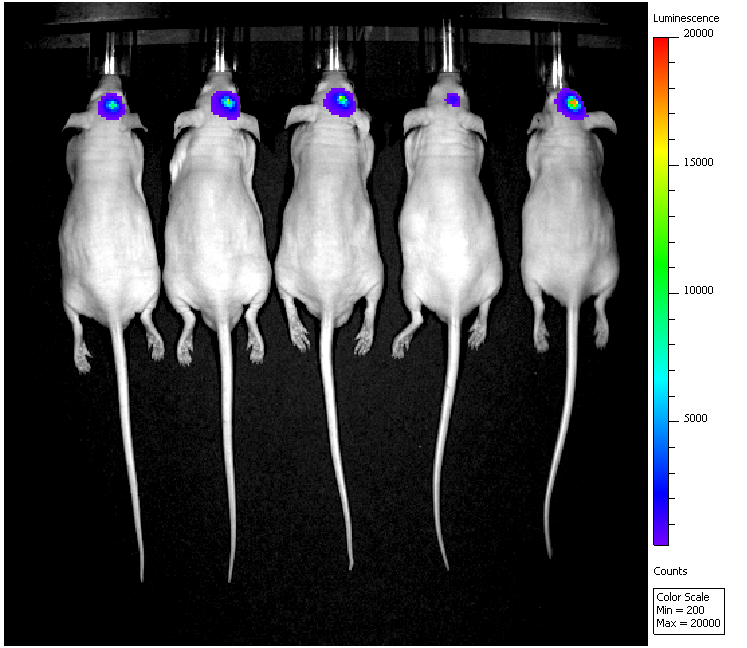

Supplement: Supplementary file 9 — Source data Fig. 4 [file 44321_2025_287_MOESM9_ESM.zip › Figure 4 /4A/IVIS Image WT_group3_14dpi.png]

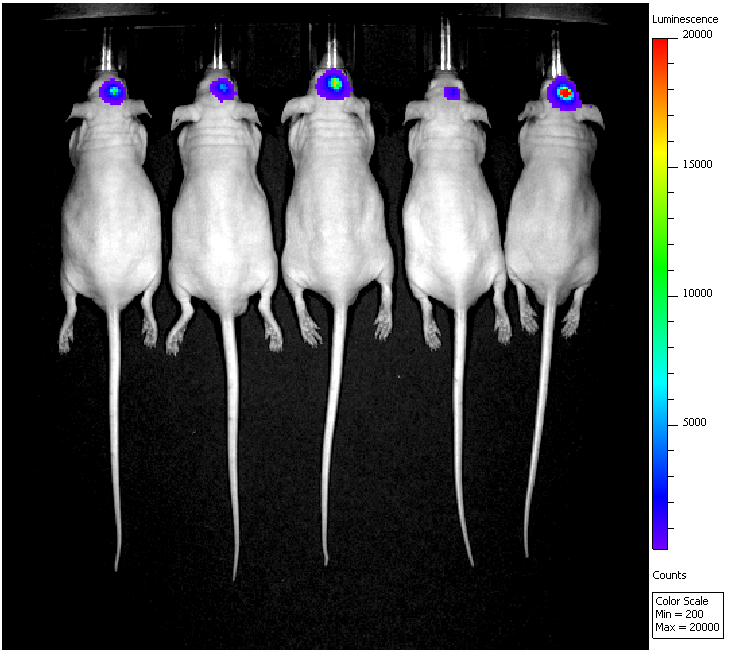

Supplement: Supplementary file 9 — Source data Fig. 4 [file 44321_2025_287_MOESM9_ESM.zip › Figure 4 /4A/IVIS Image WT_group3_21dpi.png]

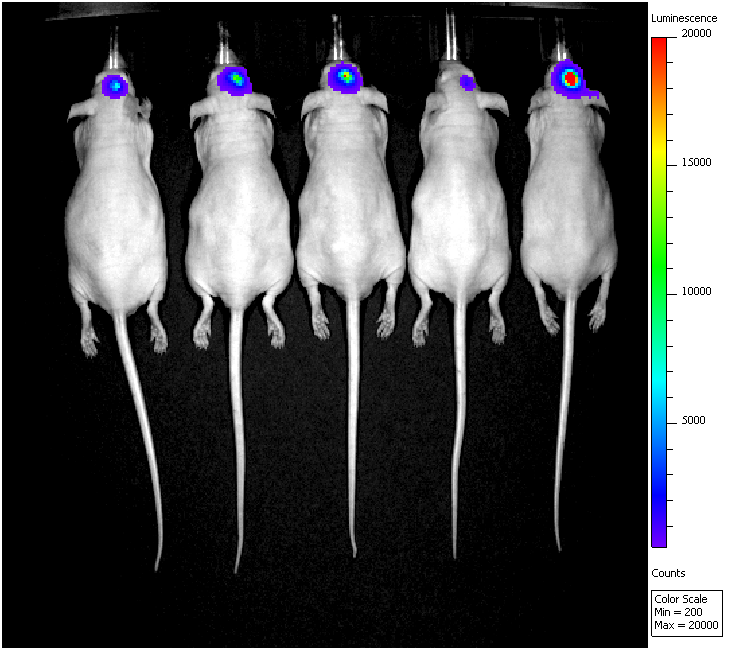

Supplement: Supplementary file 9 — Source data Fig. 4 [file 44321_2025_287_MOESM9_ESM.zip › Figure 4 /4A/IVIS Image WT_group3_28dpi.png]

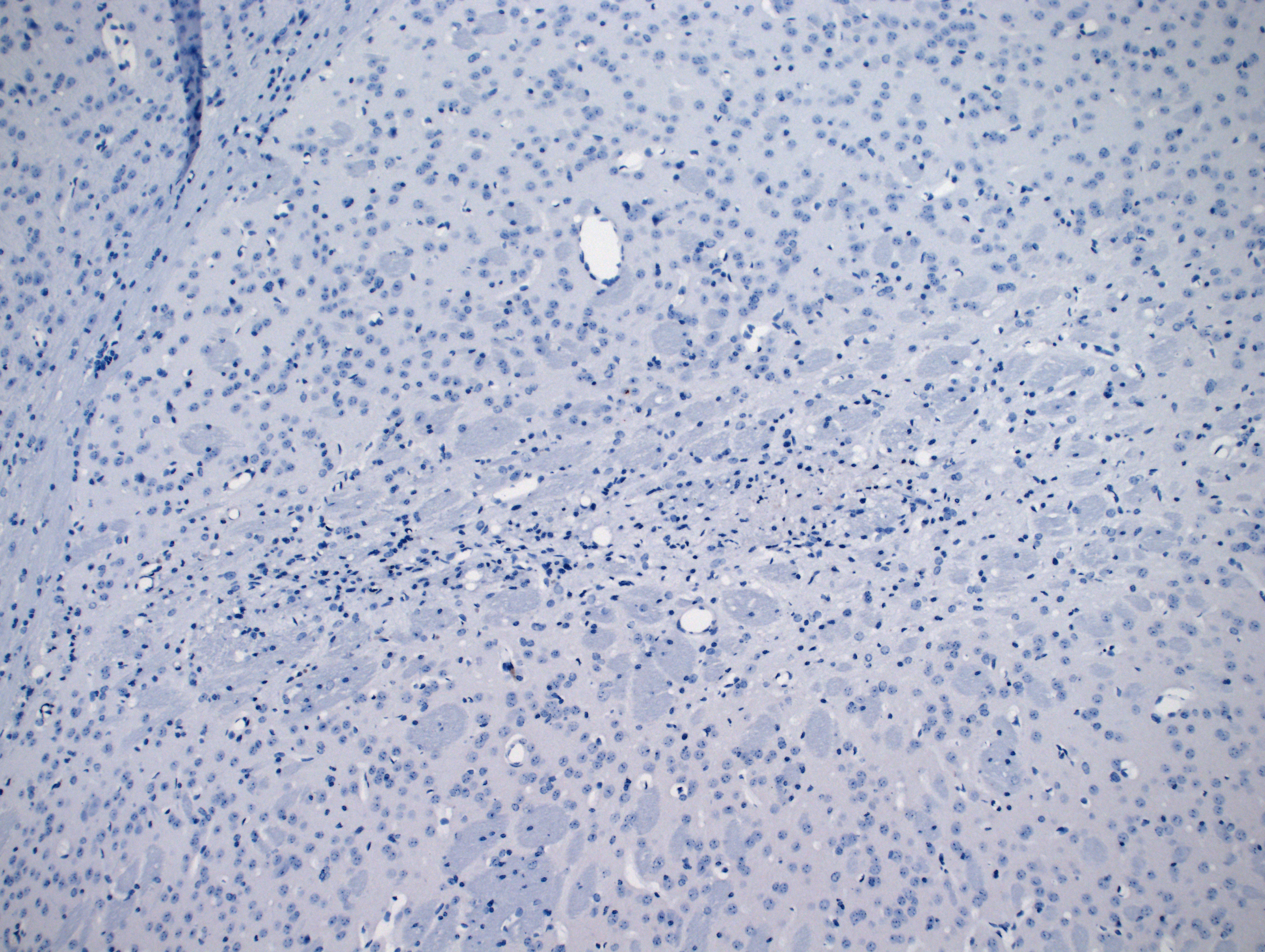

Supplement: Supplementary file 9 — Source data Fig. 4 [file 44321_2025_287_MOESM9_ESM.zip › Figure 4 /4E/Image Ki67 CR.png]

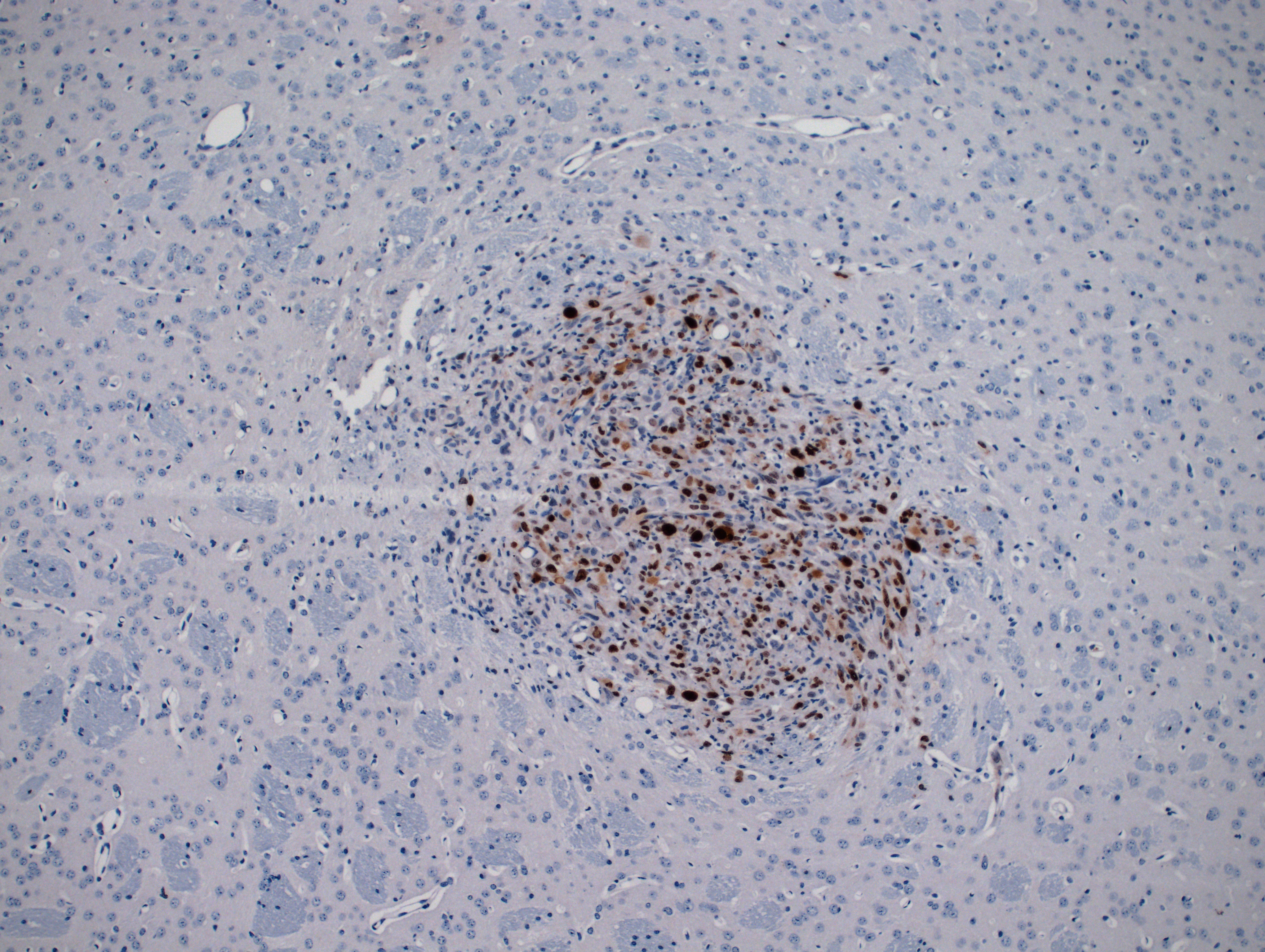

Supplement: Supplementary file 9 — Source data Fig. 4 [file 44321_2025_287_MOESM9_ESM.zip › Figure 4 /4E/Image Ki67 WT.png]

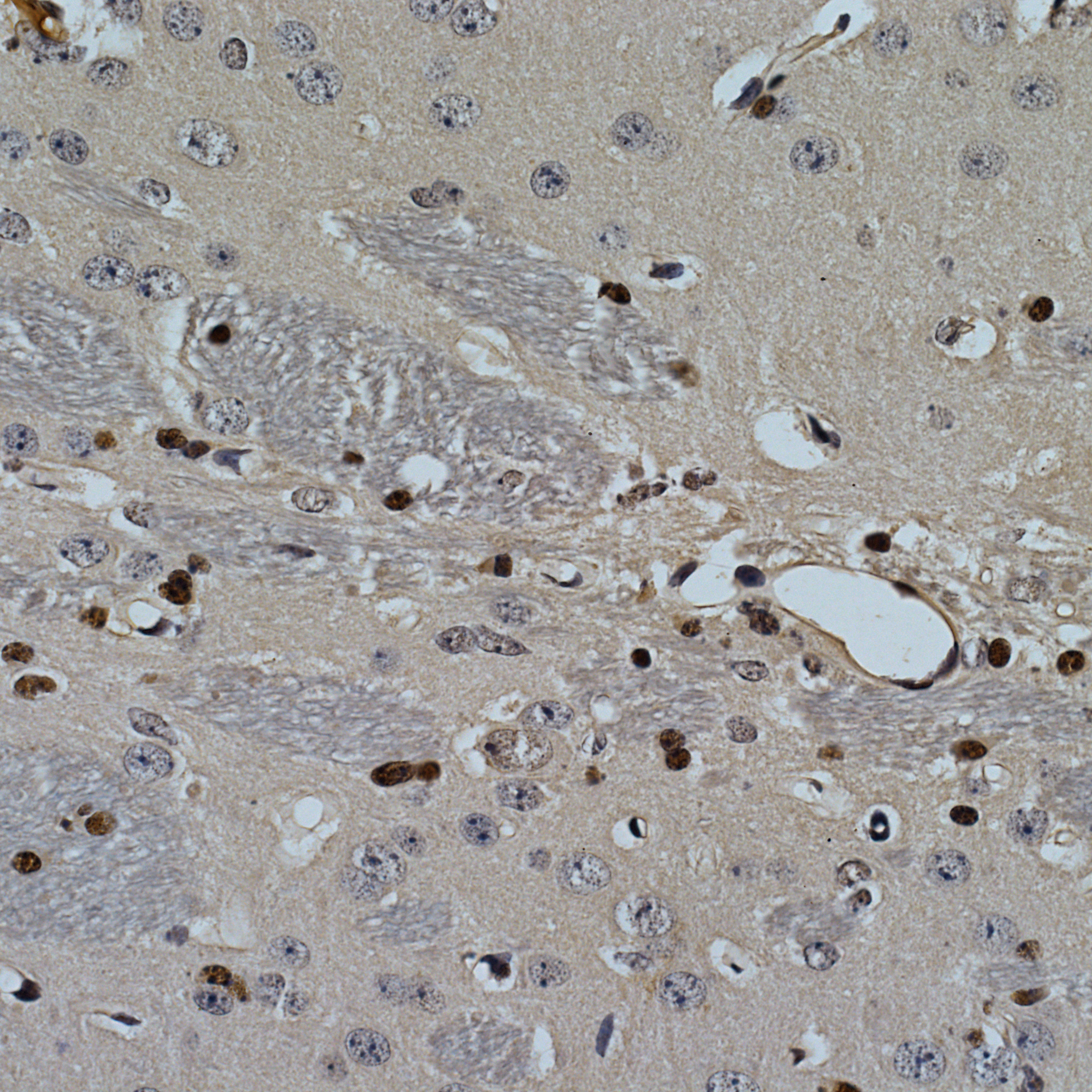

Supplement: Supplementary file 9 — Source data Fig. 4 [file 44321_2025_287_MOESM9_ESM.zip › Figure 4 /4E/Image PCNA CR.tif]

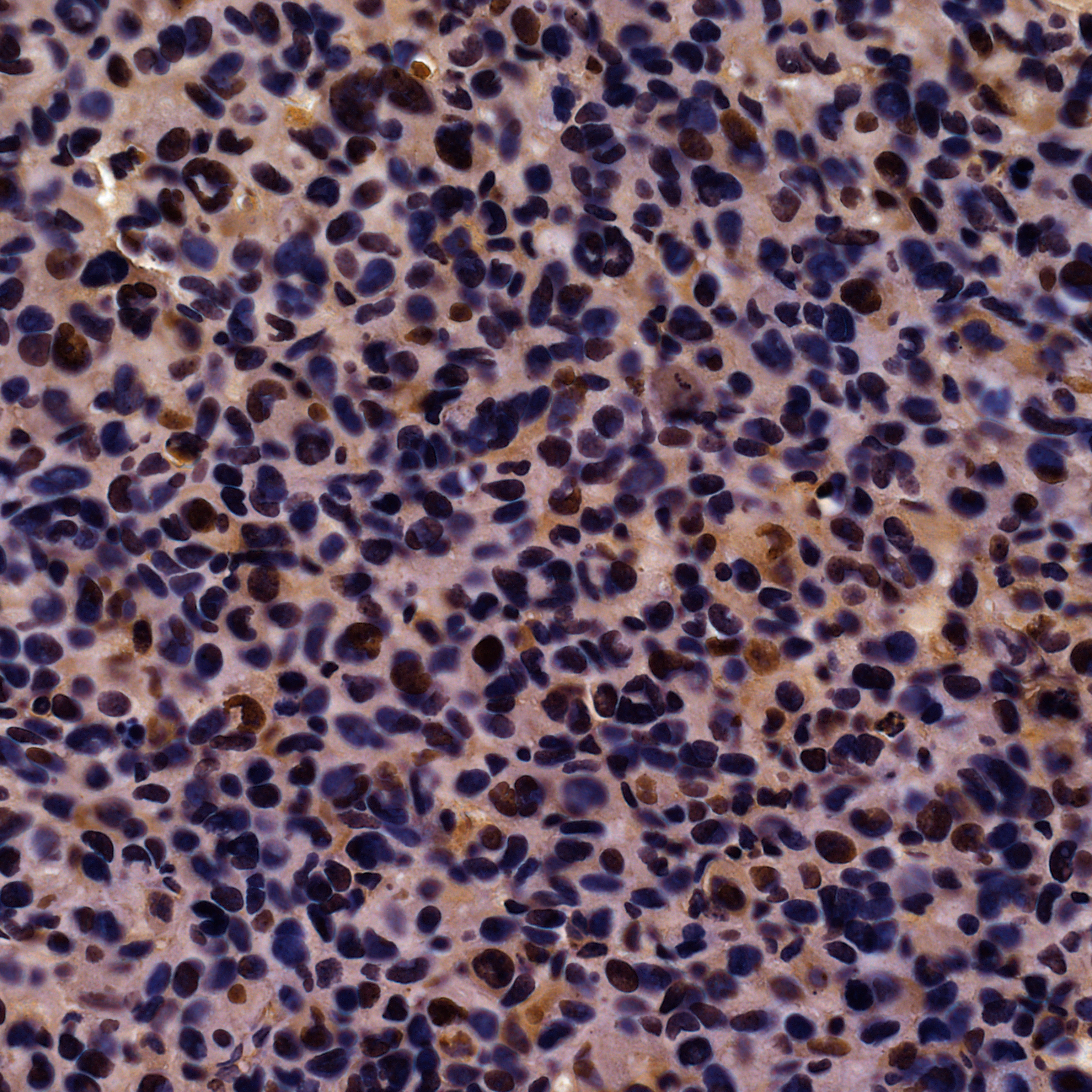

Supplement: Supplementary file 9 — Source data Fig. 4 [file 44321_2025_287_MOESM9_ESM.zip › Figure 4 /4E/Image PCNA WT.tif]

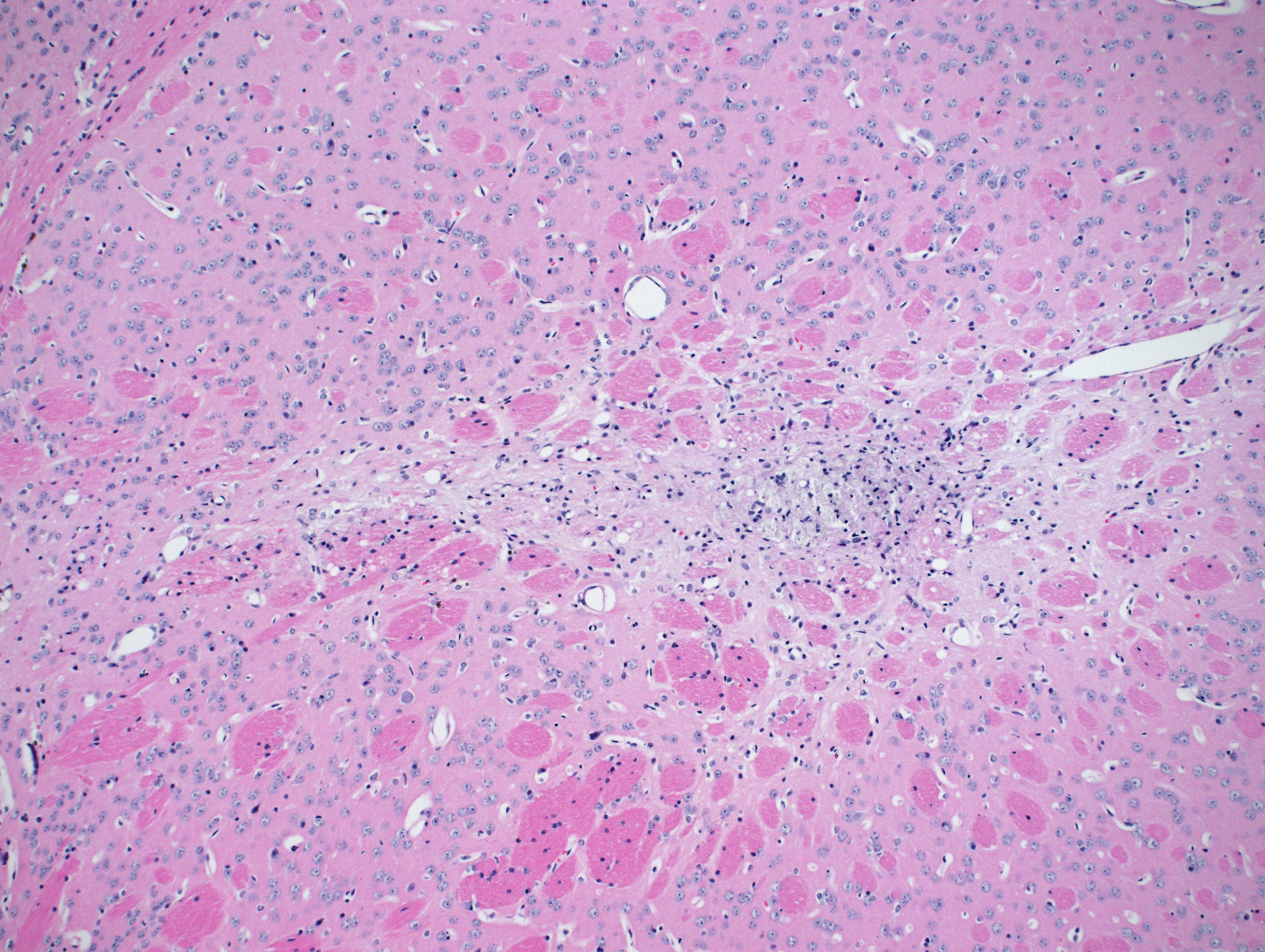

Supplement: Supplementary file 9 — Source data Fig. 4 [file 44321_2025_287_MOESM9_ESM.zip › Figure 4 /4D/Image H and E KO.jpg]

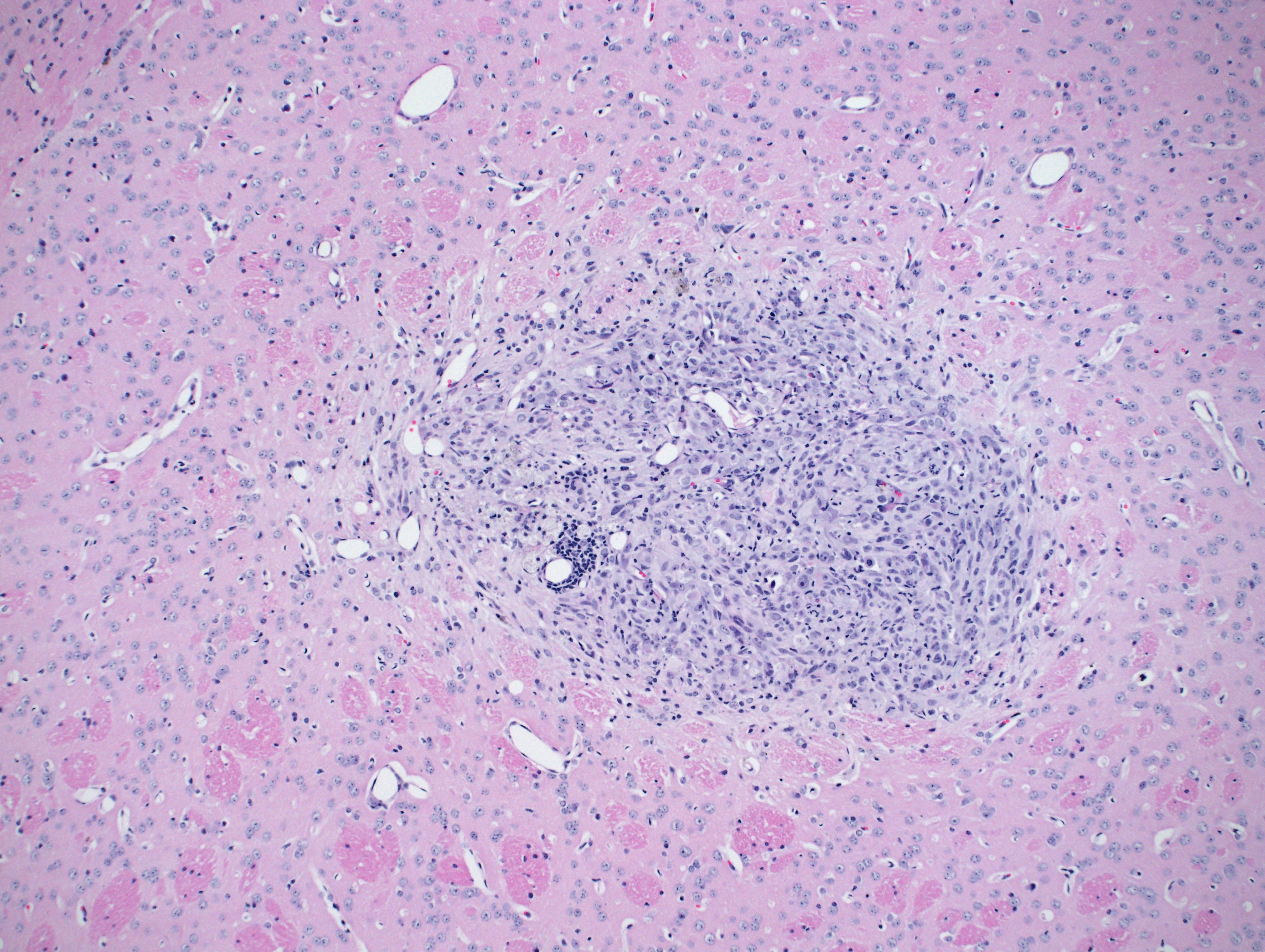

Supplement: Supplementary file 9 — Source data Fig. 4 [file 44321_2025_287_MOESM9_ESM.zip › Figure 4 /4D/Image H and E WT.jpg]

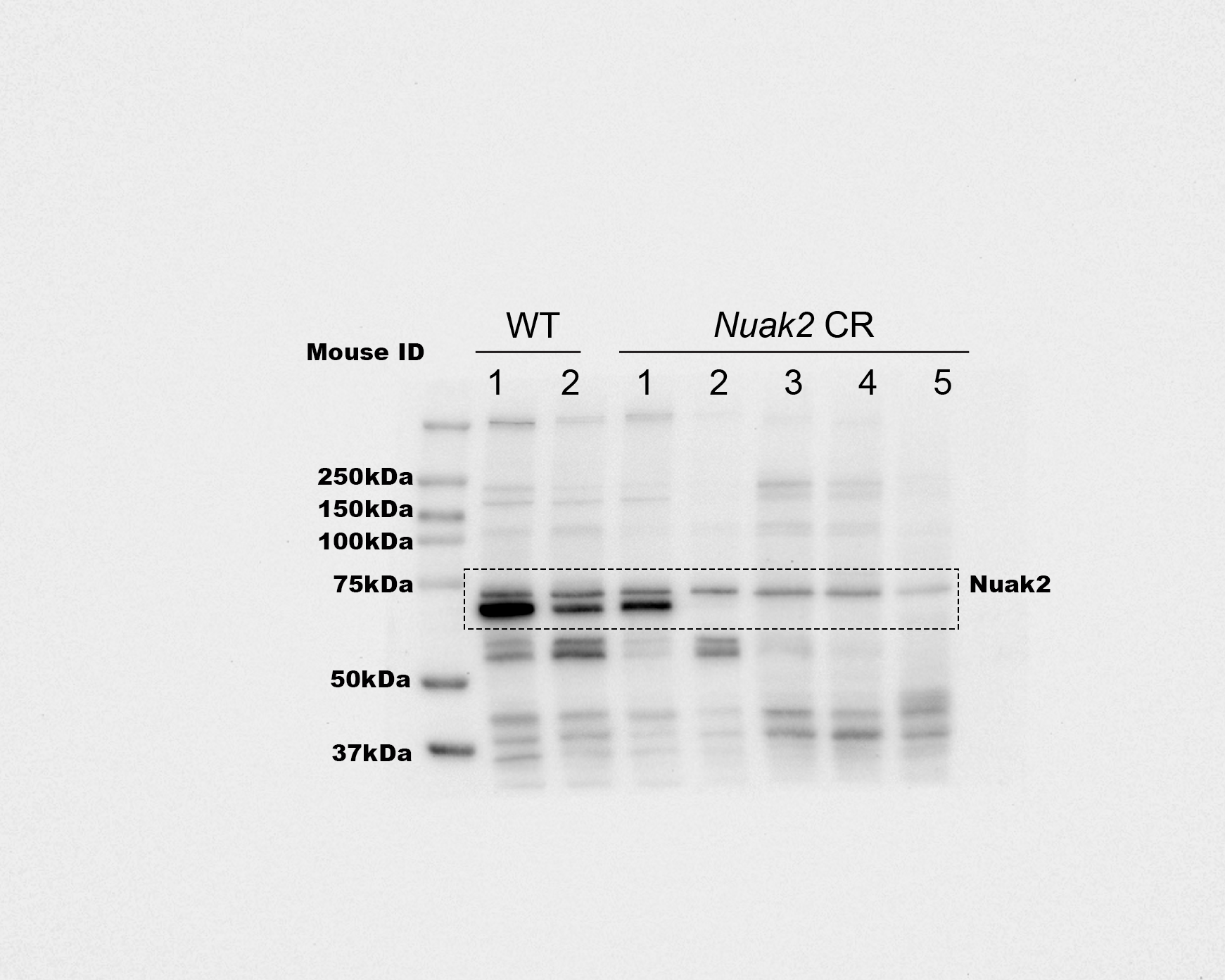

Supplement: Supplementary file 10 — Source data Fig. 5 [file 44321_2025_287_MOESM10_ESM.zip › Figure 5 /5B/Western IUE Nuak2.tif]

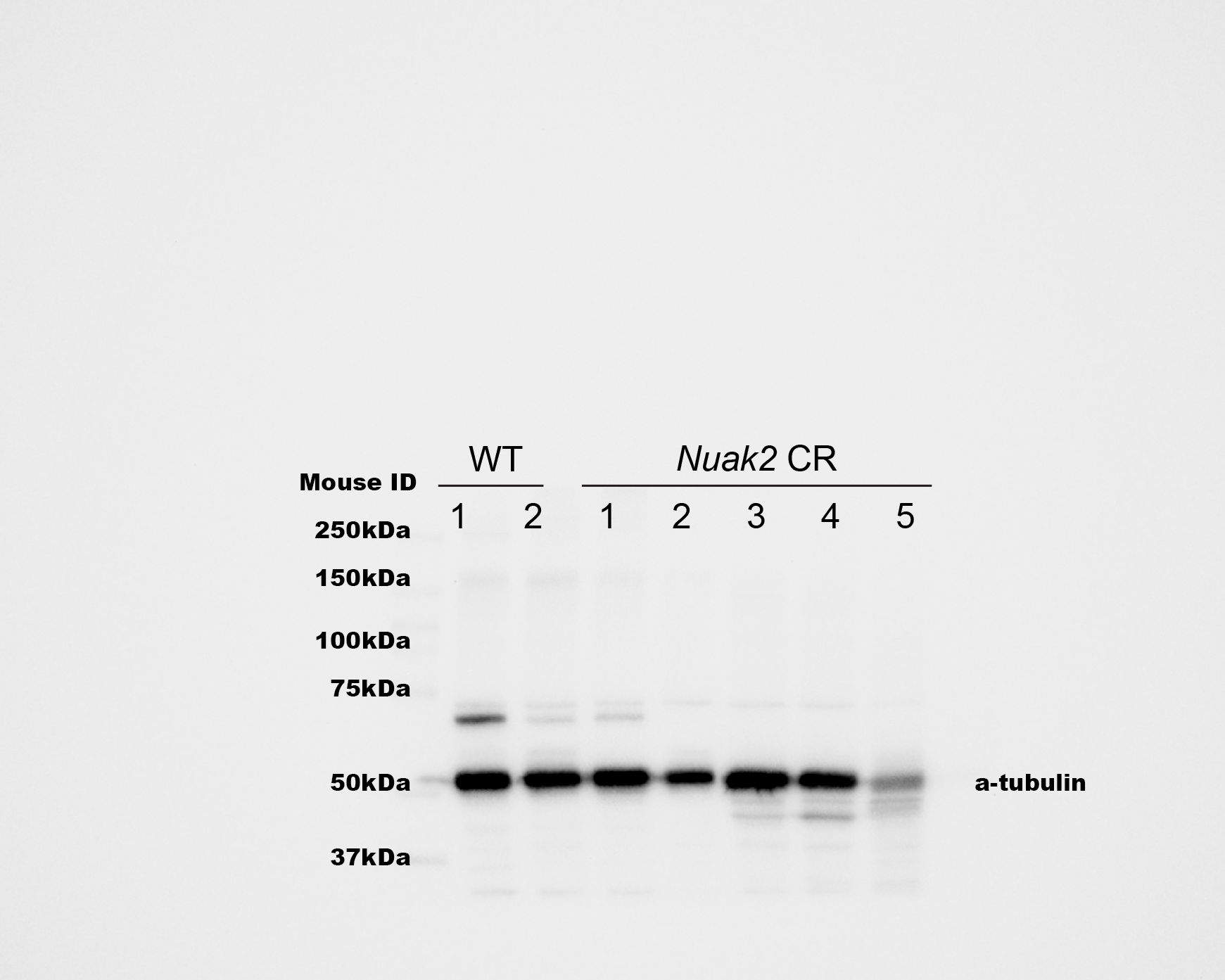

Supplement: Supplementary file 10 — Source data Fig. 5 [file 44321_2025_287_MOESM10_ESM.zip › Figure 5 /5B/Western IUE a-tubulin.tif]

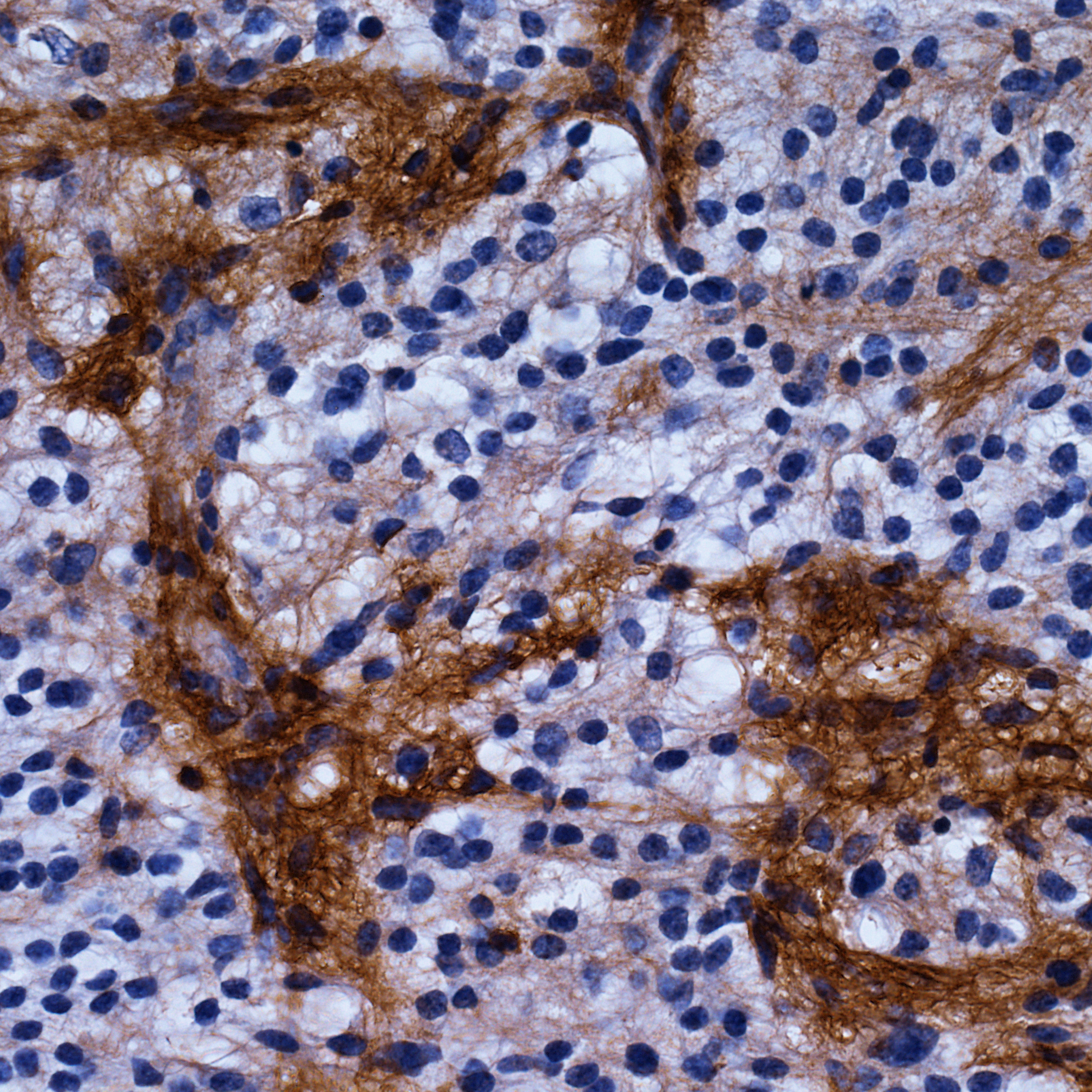

Supplement: Supplementary file 10 — Source data Fig. 5 [file 44321_2025_287_MOESM10_ESM.zip › Figure 5 /5E (histology images)/Image CD44 Control.tif]

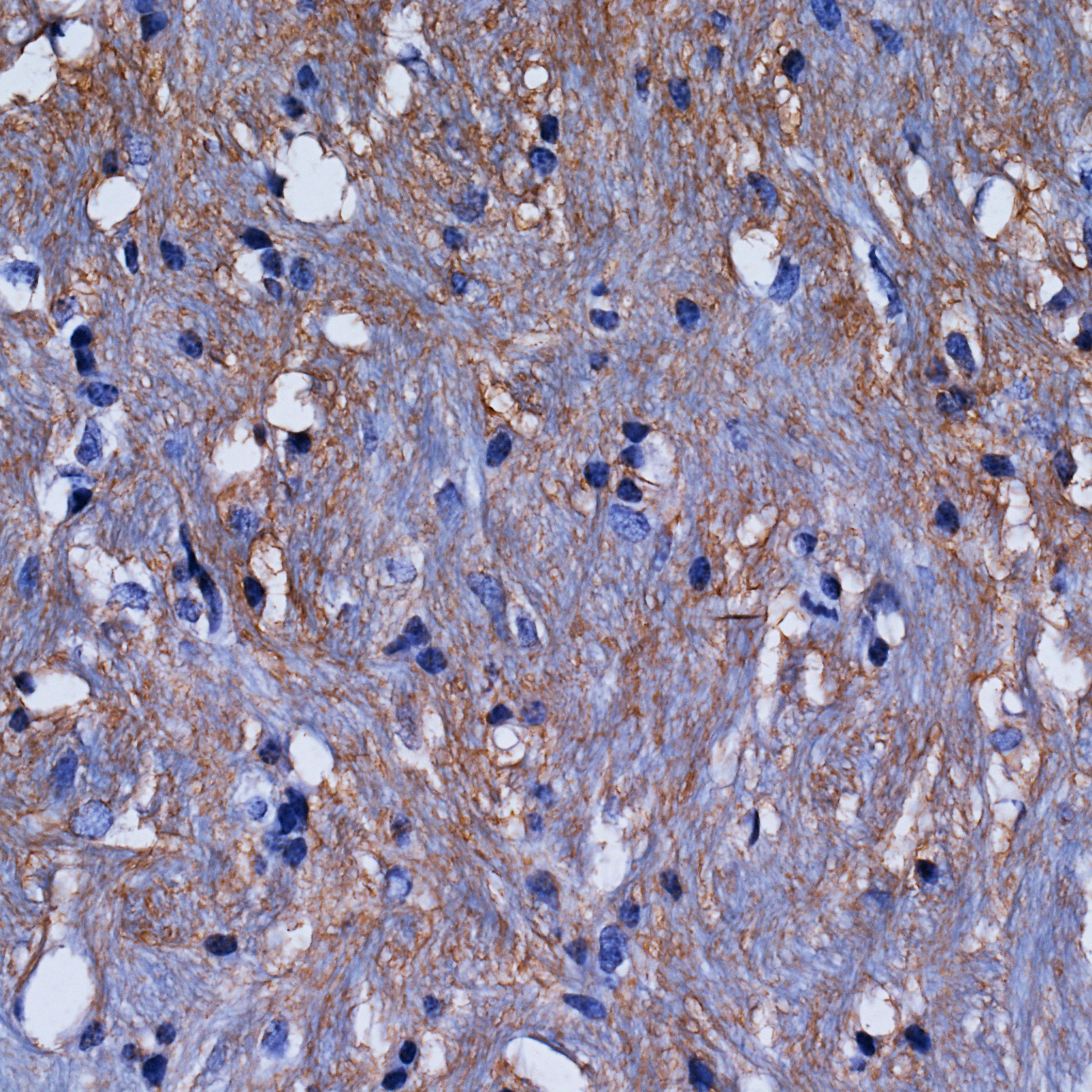

Supplement: Supplementary file 10 — Source data Fig. 5 [file 44321_2025_287_MOESM10_ESM.zip › Figure 5 /5E (histology images)/Image CD44 CR.tif]

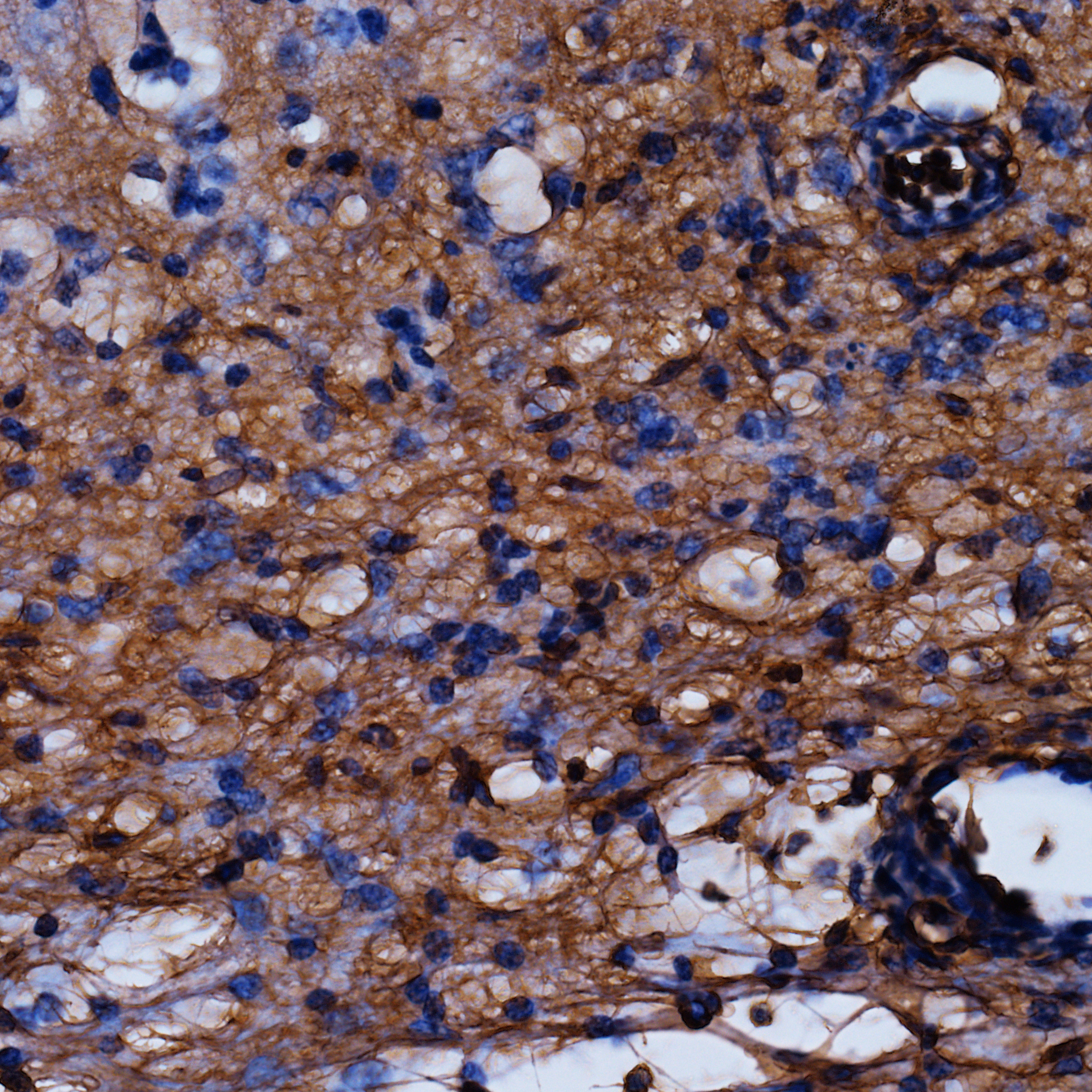

Supplement: Supplementary file 10 — Source data Fig. 5 [file 44321_2025_287_MOESM10_ESM.zip › Figure 5 /5E (histology images)/Image CD44 OE.tif]

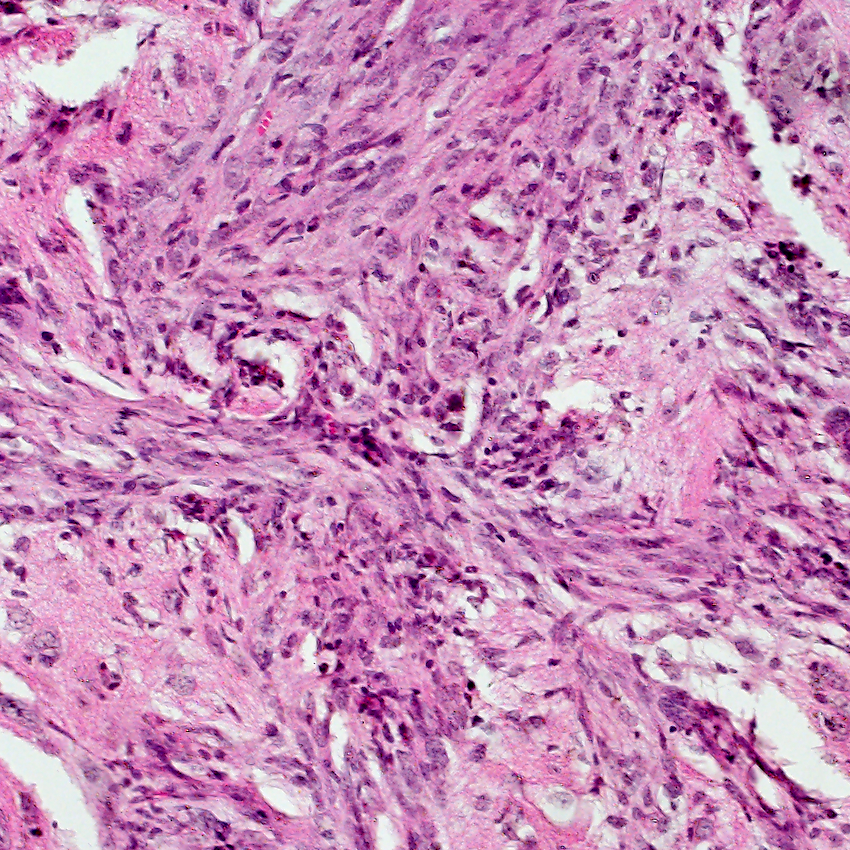

Supplement: Supplementary file 10 — Source data Fig. 5 [file 44321_2025_287_MOESM10_ESM.zip › Figure 5 /5E (histology images)/Image H and E Control.tif]

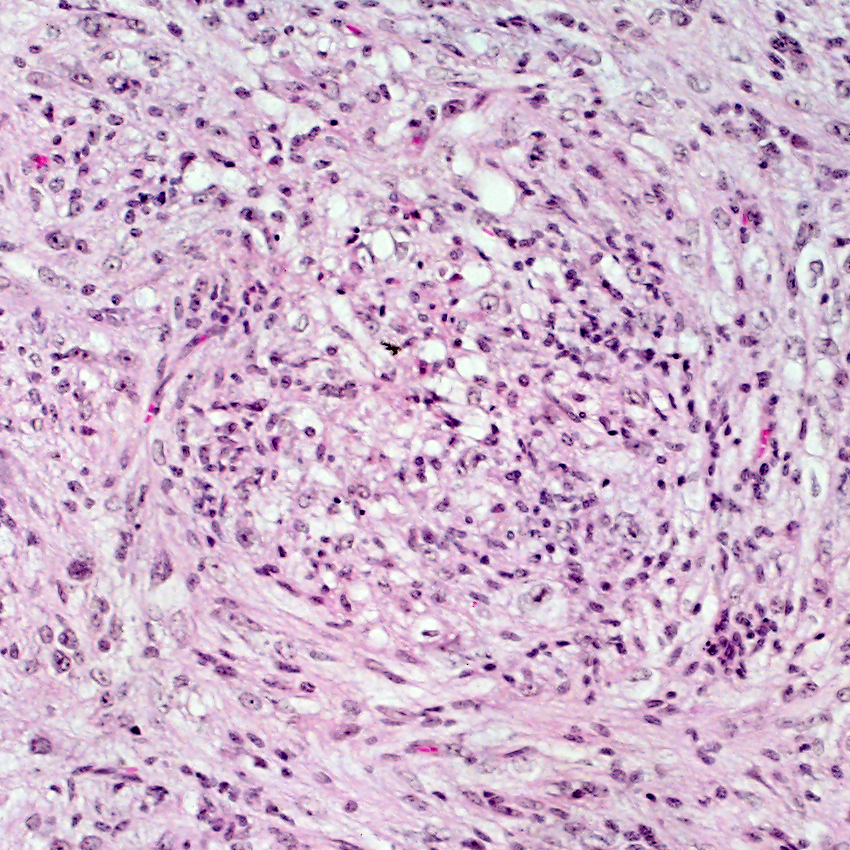

Supplement: Supplementary file 10 — Source data Fig. 5 [file 44321_2025_287_MOESM10_ESM.zip › Figure 5 /5E (histology images)/Image H and E CR.tif]

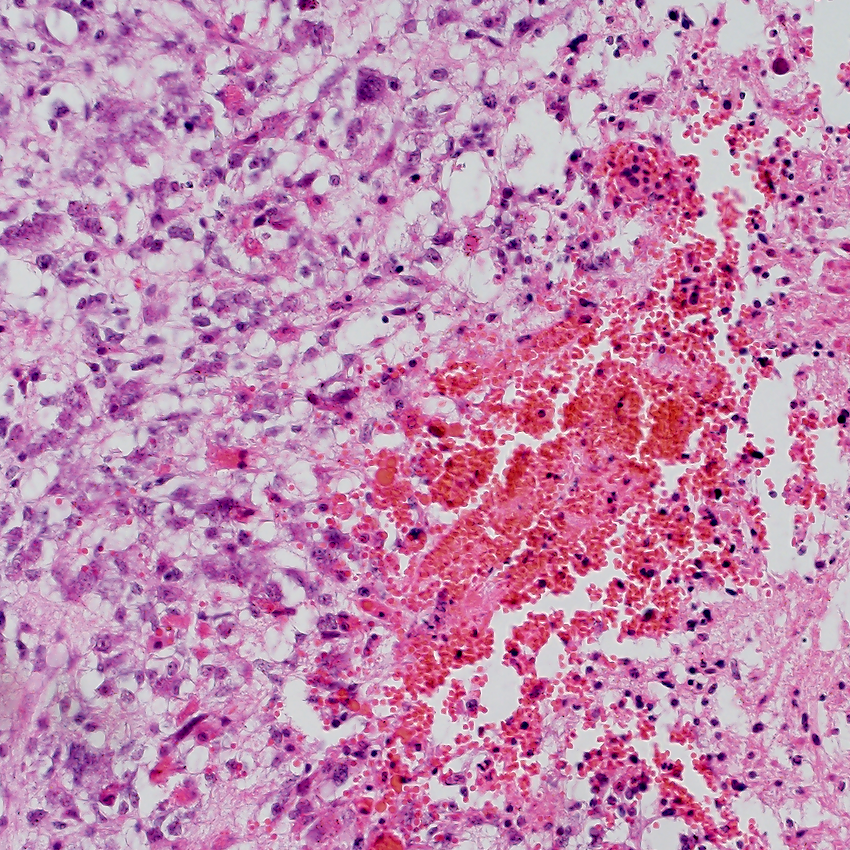

Supplement: Supplementary file 10 — Source data Fig. 5 [file 44321_2025_287_MOESM10_ESM.zip › Figure 5 /5E (histology images)/Image H and E OX.tif]

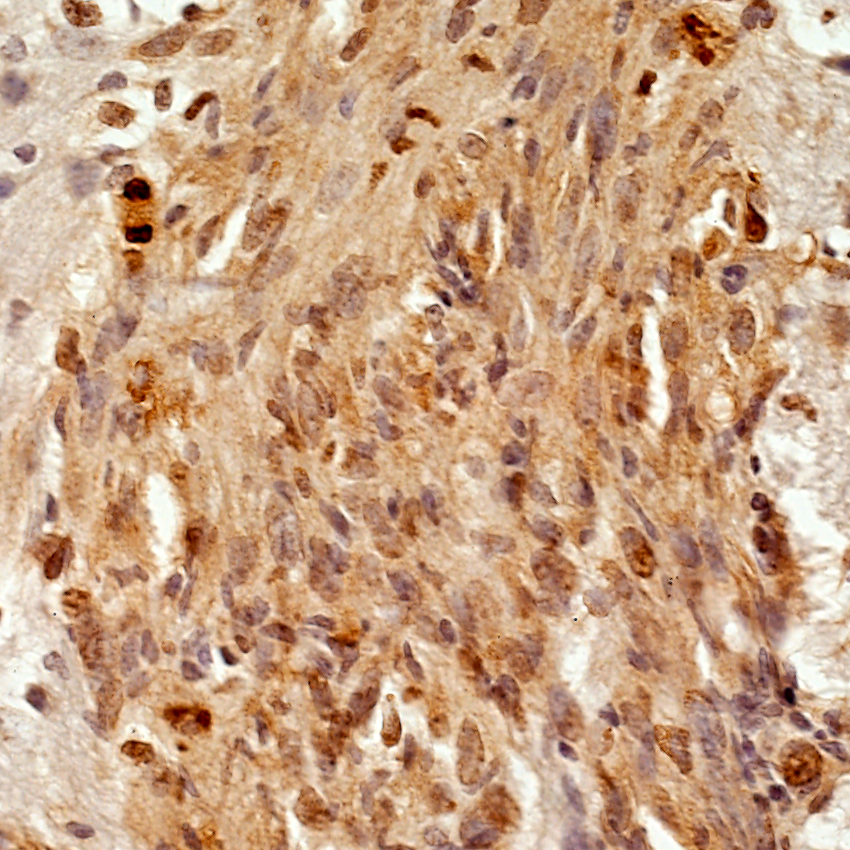

Supplement: Supplementary file 10 — Source data Fig. 5 [file 44321_2025_287_MOESM10_ESM.zip › Figure 5 /5E (histology images)/Image Ki67 Control.tif]

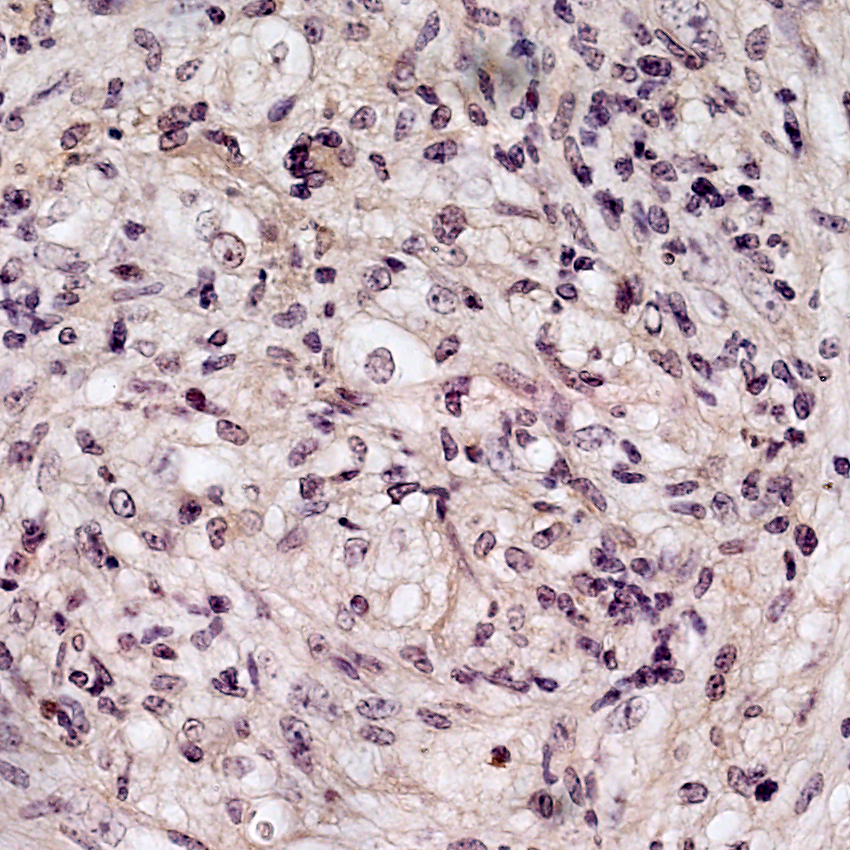

Supplement: Supplementary file 10 — Source data Fig. 5 [file 44321_2025_287_MOESM10_ESM.zip › Figure 5 /5E (histology images)/Image Ki67 CR.tif]

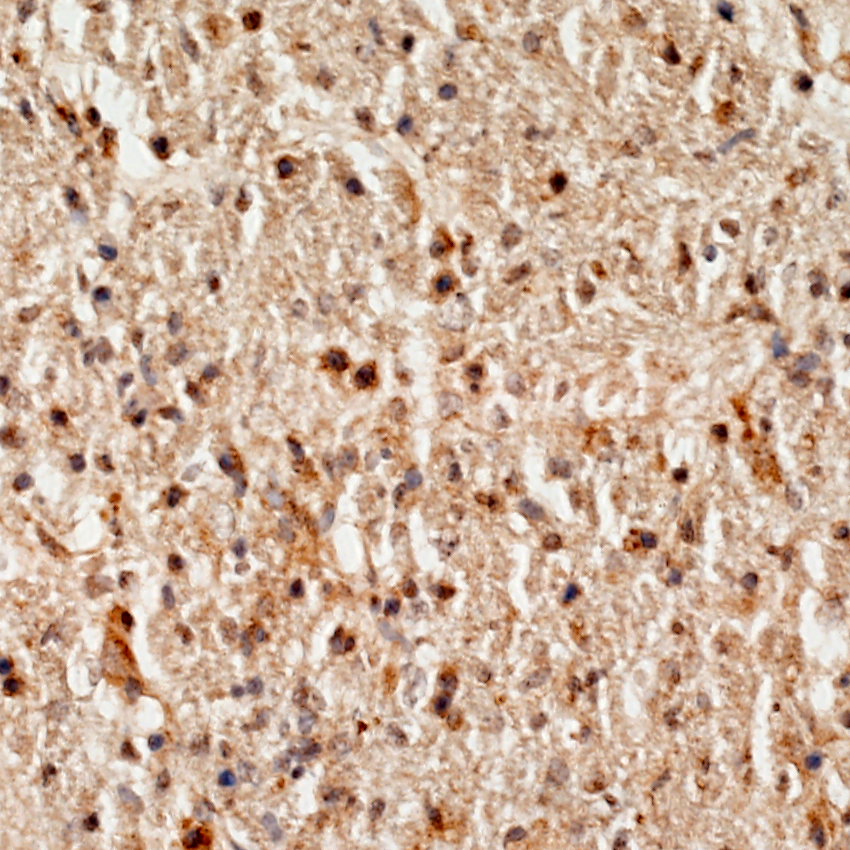

Supplement: Supplementary file 10 — Source data Fig. 5 [file 44321_2025_287_MOESM10_ESM.zip › Figure 5 /5E (histology images)/Image Ki67 OX.tif]

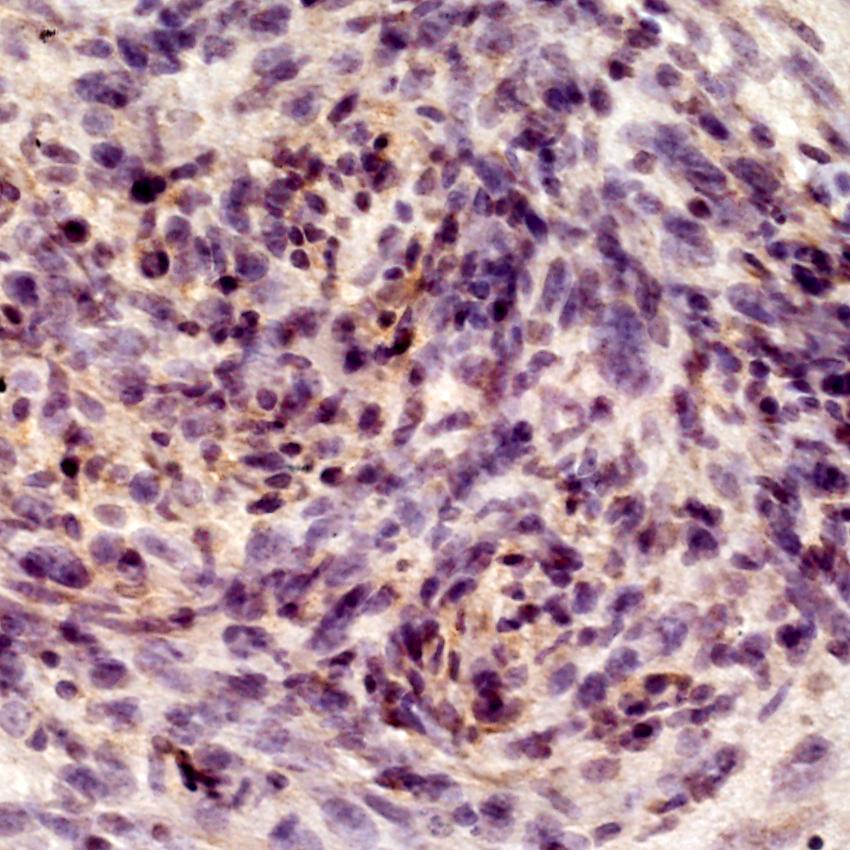

Supplement: Supplementary file 10 — Source data Fig. 5 [file 44321_2025_287_MOESM10_ESM.zip › Figure 5 /5E (histology images)/Image Nuak2 Control.tif]

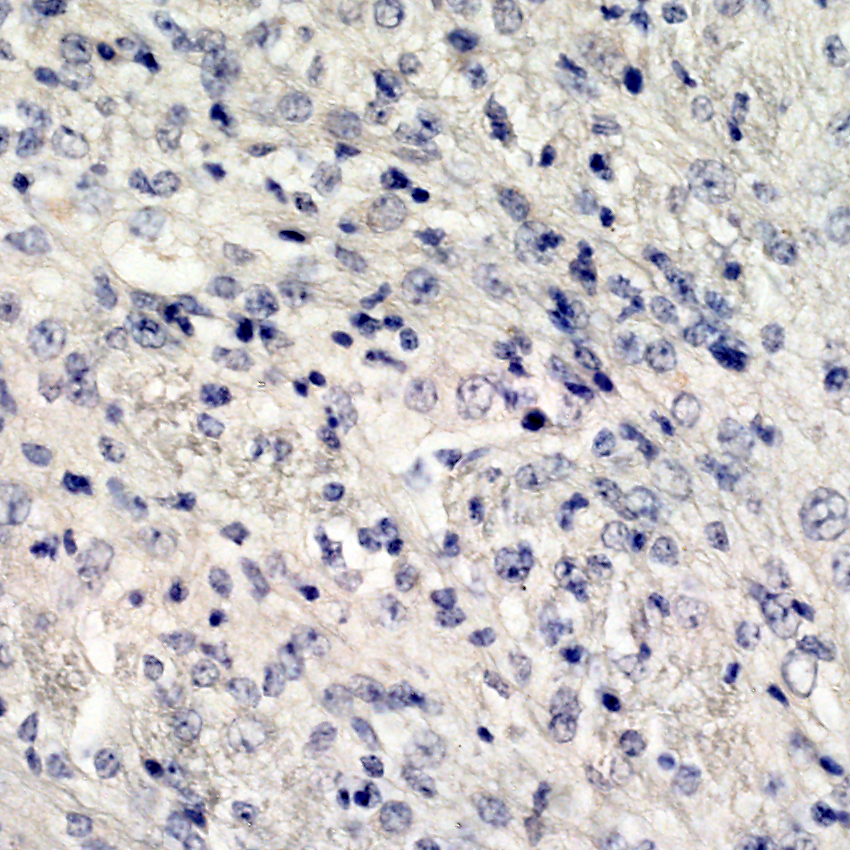

Supplement: Supplementary file 10 — Source data Fig. 5 [file 44321_2025_287_MOESM10_ESM.zip › Figure 5 /5E (histology images)/Image Nuak2 CR.tif]

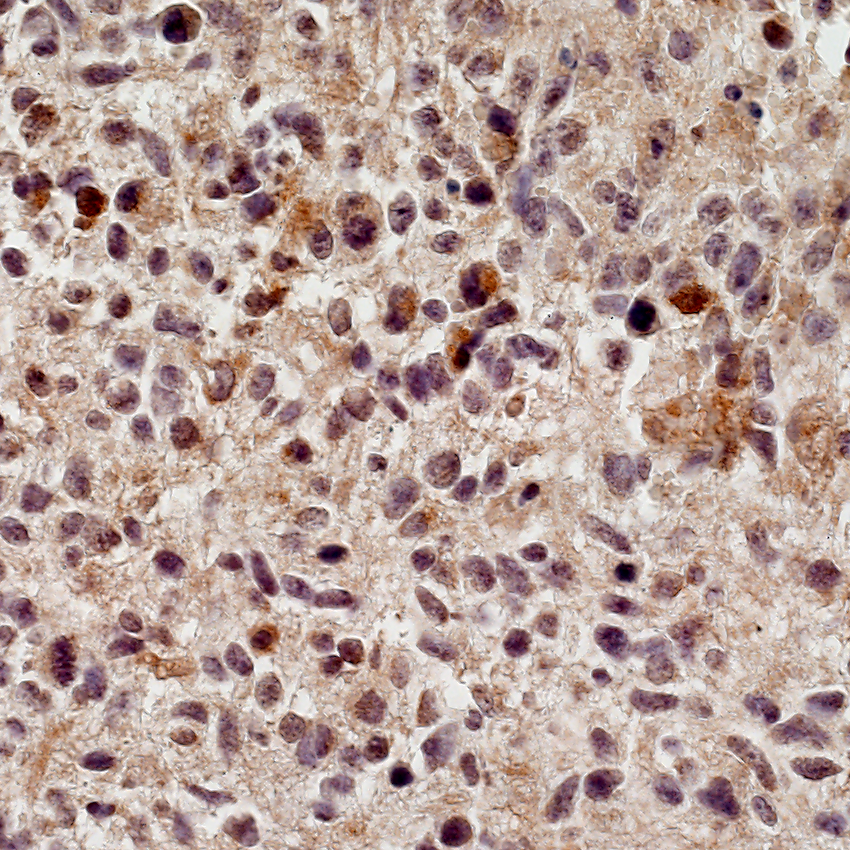

Supplement: Supplementary file 10 — Source data Fig. 5 [file 44321_2025_287_MOESM10_ESM.zip › Figure 5 /5E (histology images)/Image Nuak2 OX.tif]

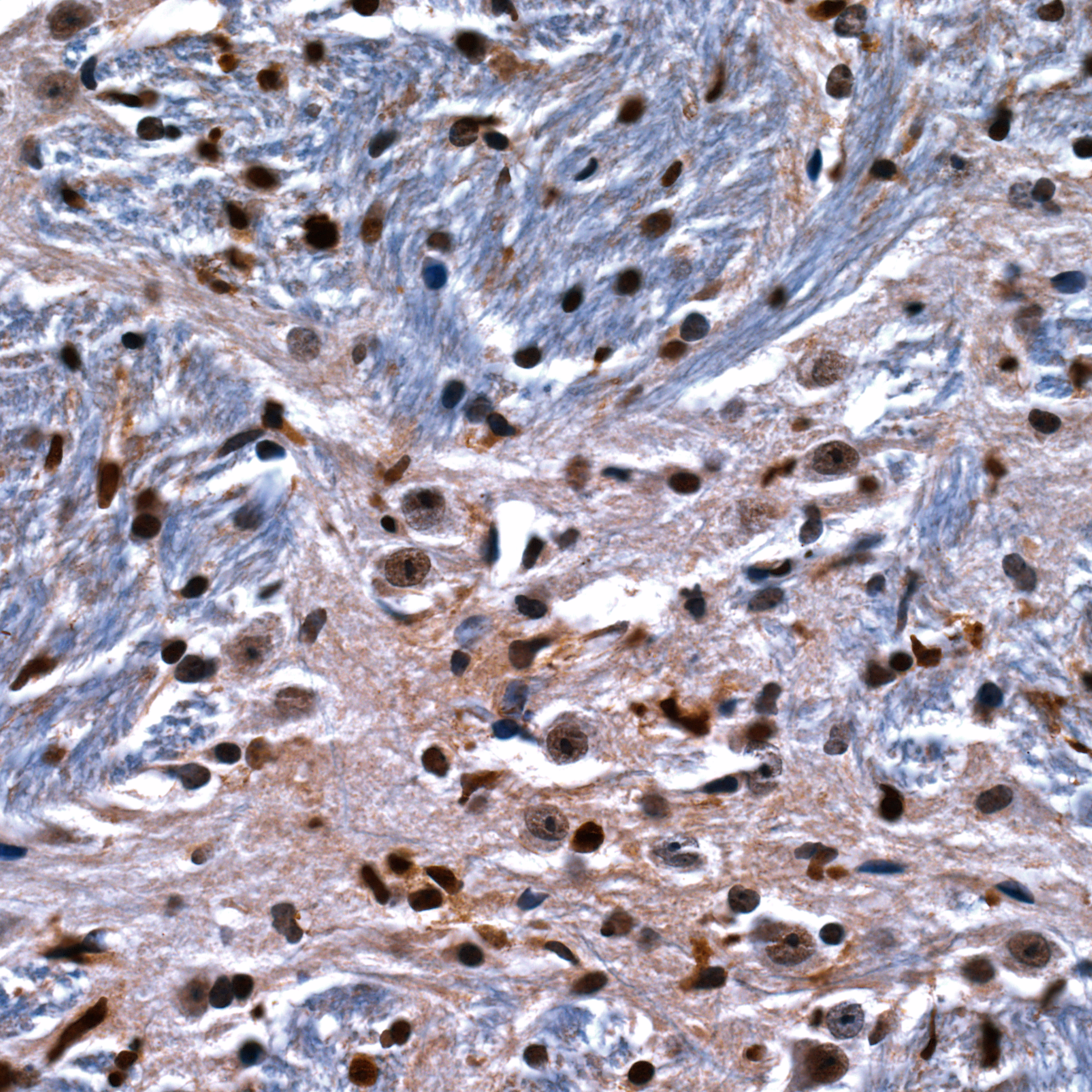

Supplement: Supplementary file 10 — Source data Fig. 5 [file 44321_2025_287_MOESM10_ESM.zip › Figure 5 /5E (histology images)/Image PCNA Control.tif]

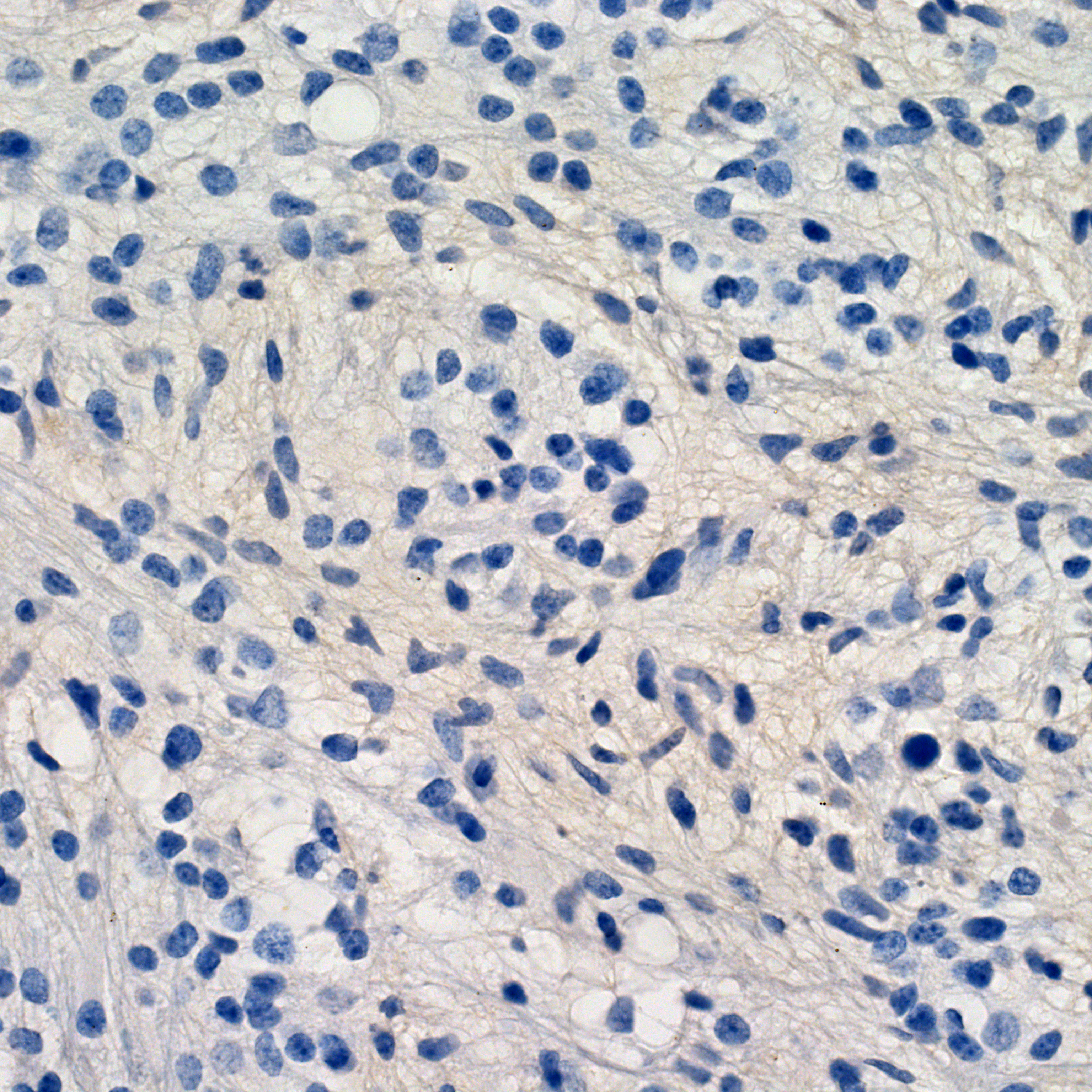

Supplement: Supplementary file 10 — Source data Fig. 5 [file 44321_2025_287_MOESM10_ESM.zip › Figure 5 /5E (histology images)/Image PCNA CR.tif]

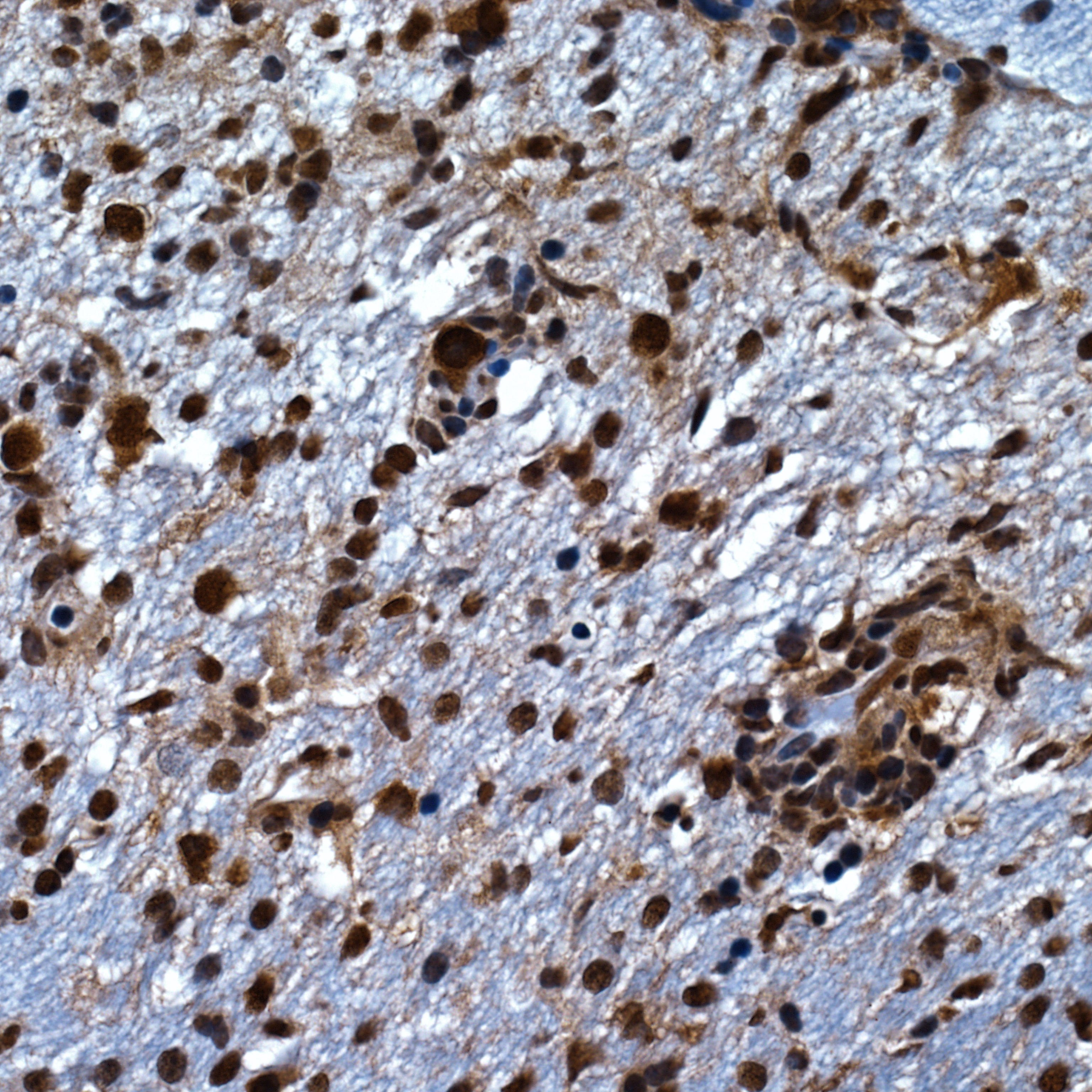

Supplement: Supplementary file 10 — Source data Fig. 5 [file 44321_2025_287_MOESM10_ESM.zip › Figure 5 /5E (histology images)/Image PCNA OX.tif]

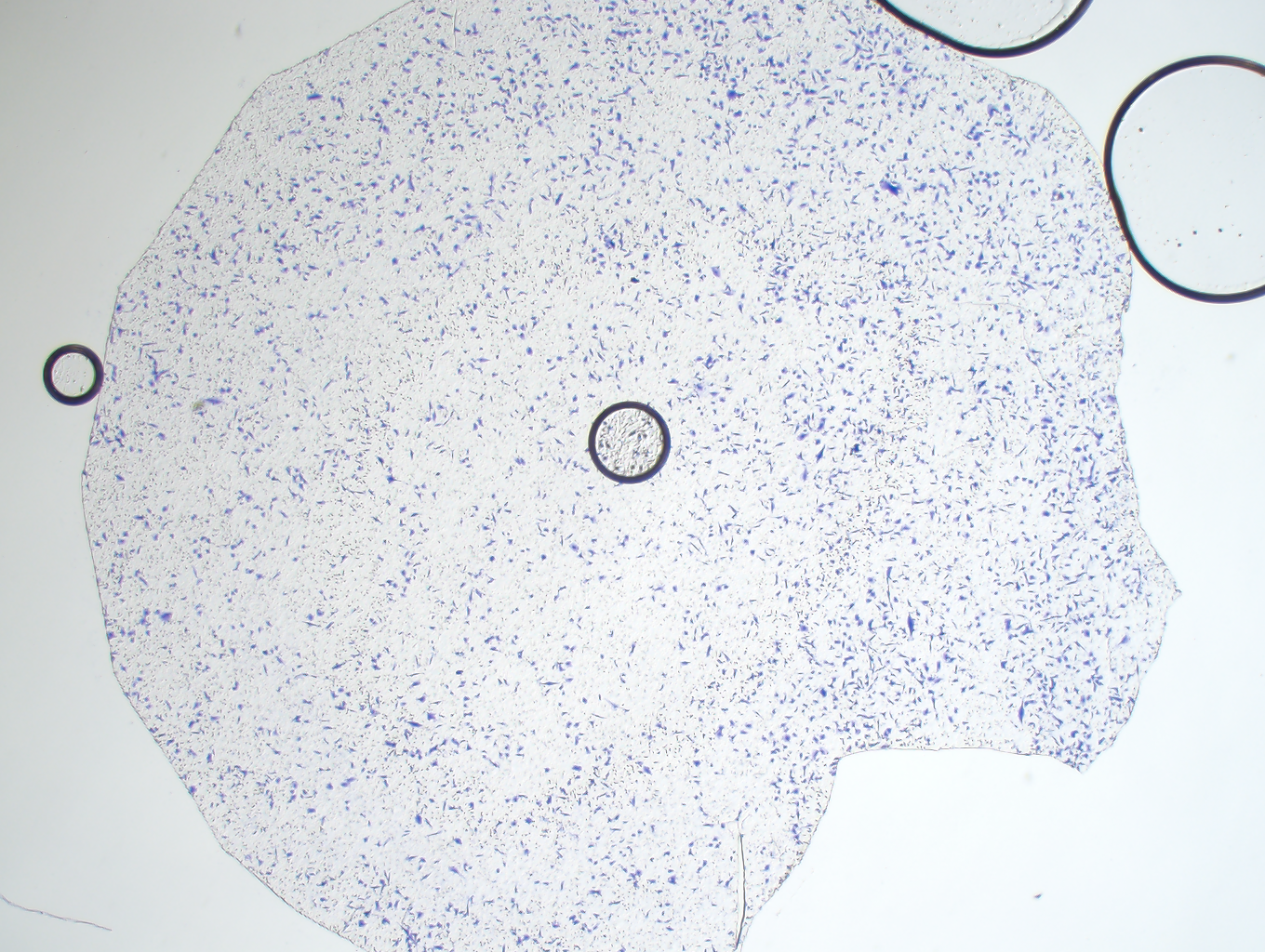

Supplement: Supplementary file 11 — Source data Fig. 6 [file 44321_2025_287_MOESM11_ESM.zip › Figure 6/6G/U251 CR3.tif]

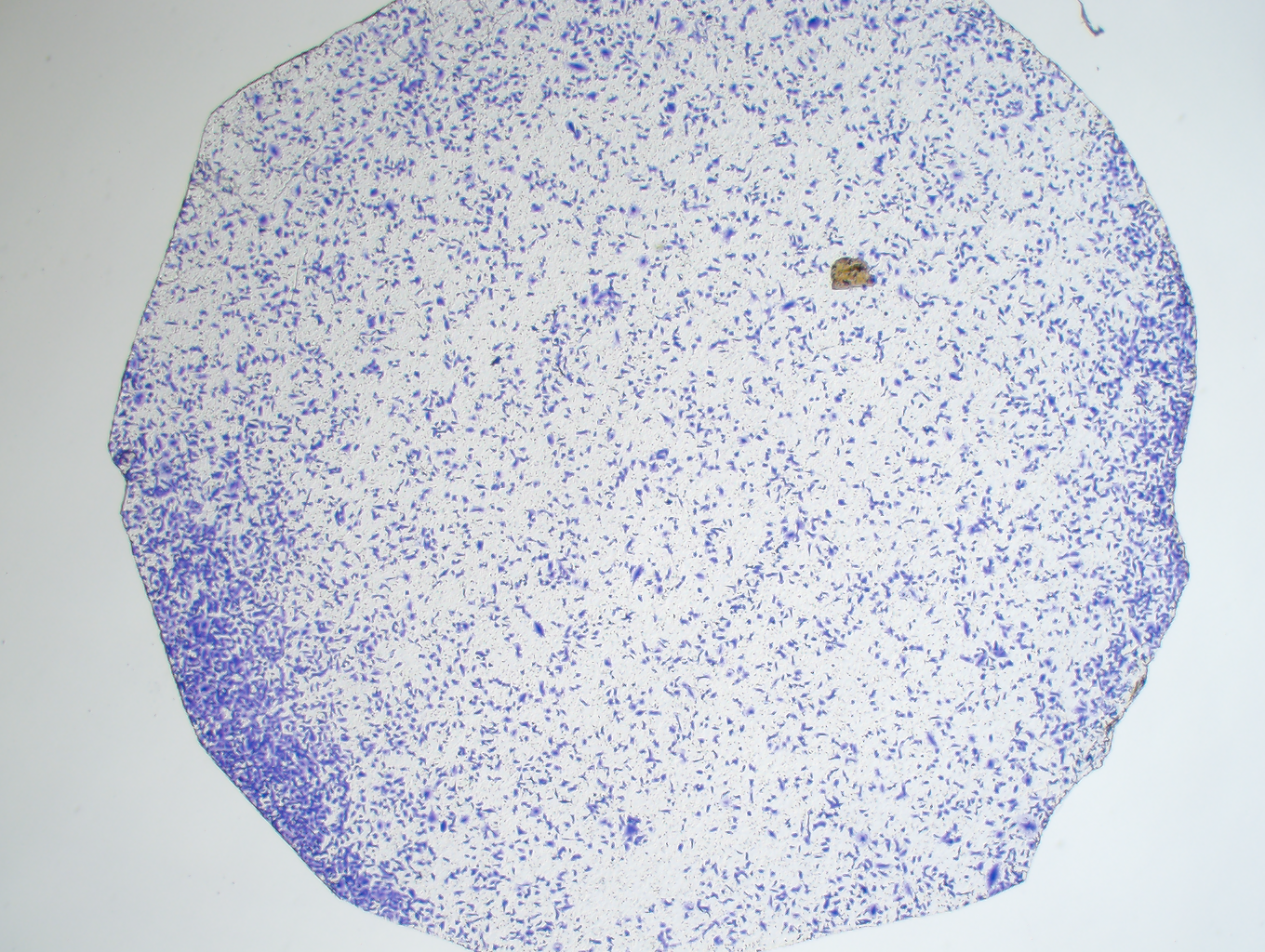

Supplement: Supplementary file 11 — Source data Fig. 6 [file 44321_2025_287_MOESM11_ESM.zip › Figure 6/6G/U251 CR2.tif]

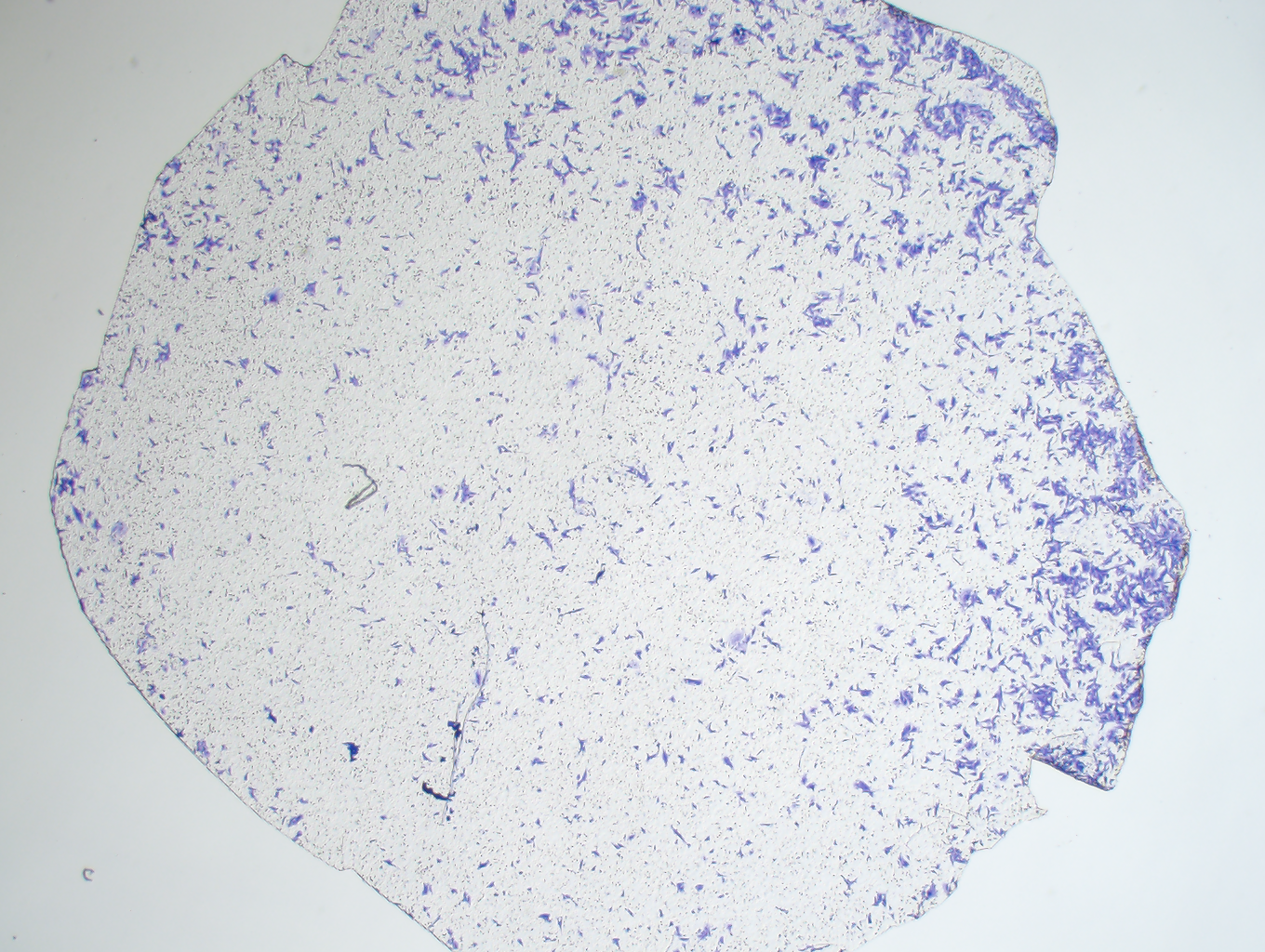

Supplement: Supplementary file 11 — Source data Fig. 6 [file 44321_2025_287_MOESM11_ESM.zip › Figure 6/6G/U251 CR1.tif]

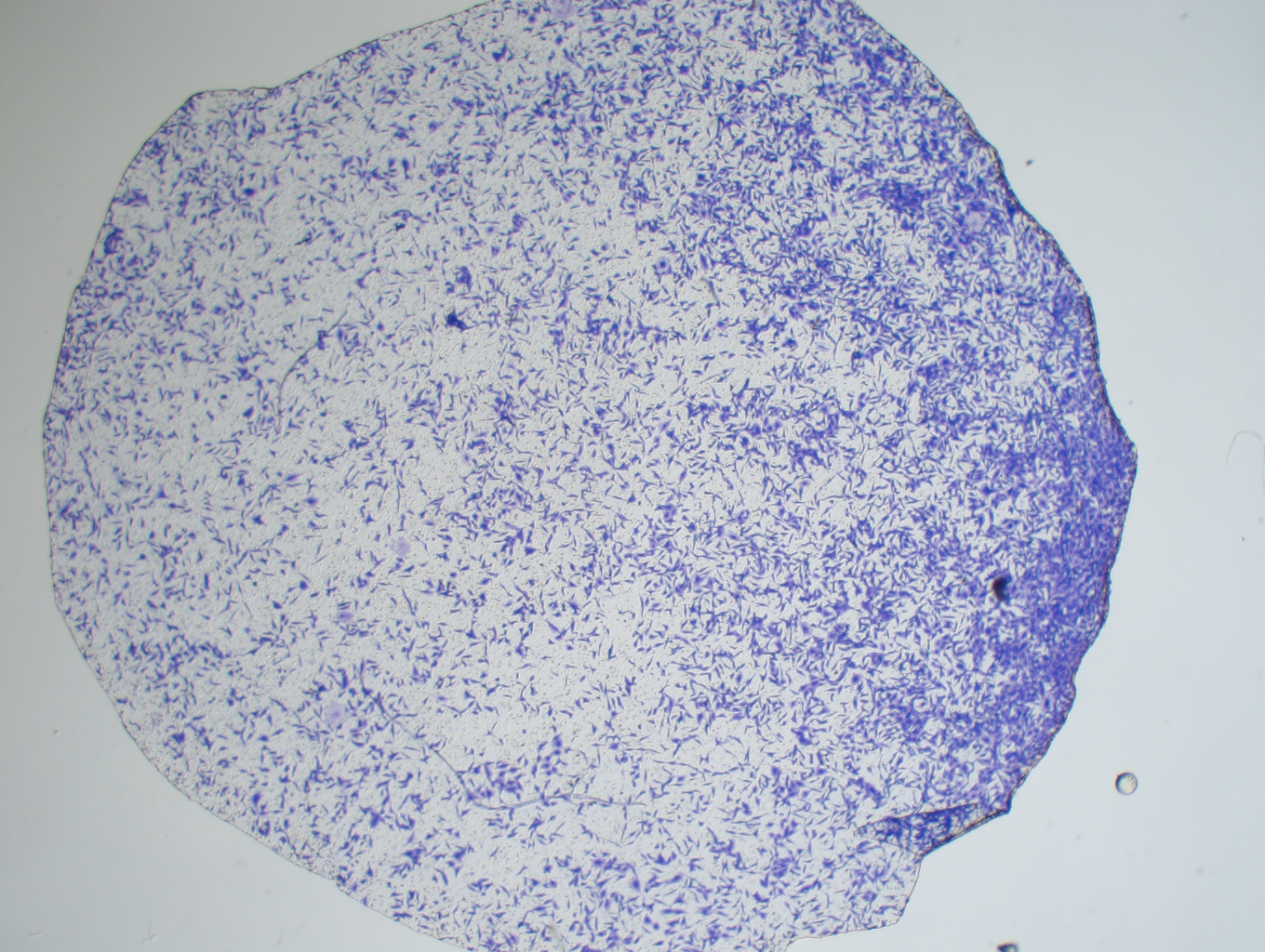

Supplement: Supplementary file 11 — Source data Fig. 6 [file 44321_2025_287_MOESM11_ESM.zip › Figure 6/6G/U251 WT.tif]

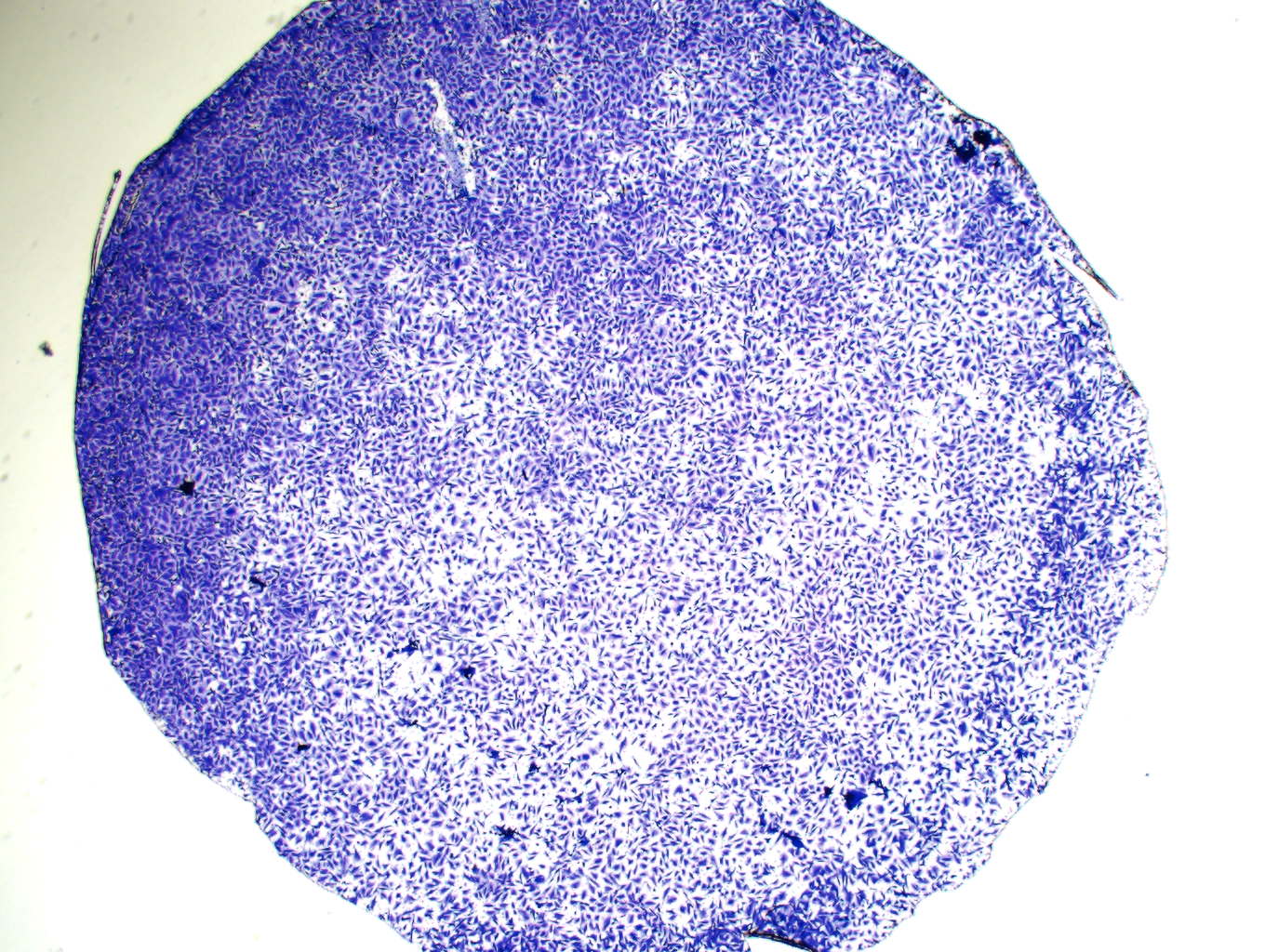

Supplement: Supplementary file 11 — Source data Fig. 6 [file 44321_2025_287_MOESM11_ESM.zip › Figure 6/6H/Transwell LN229 WT.tif]

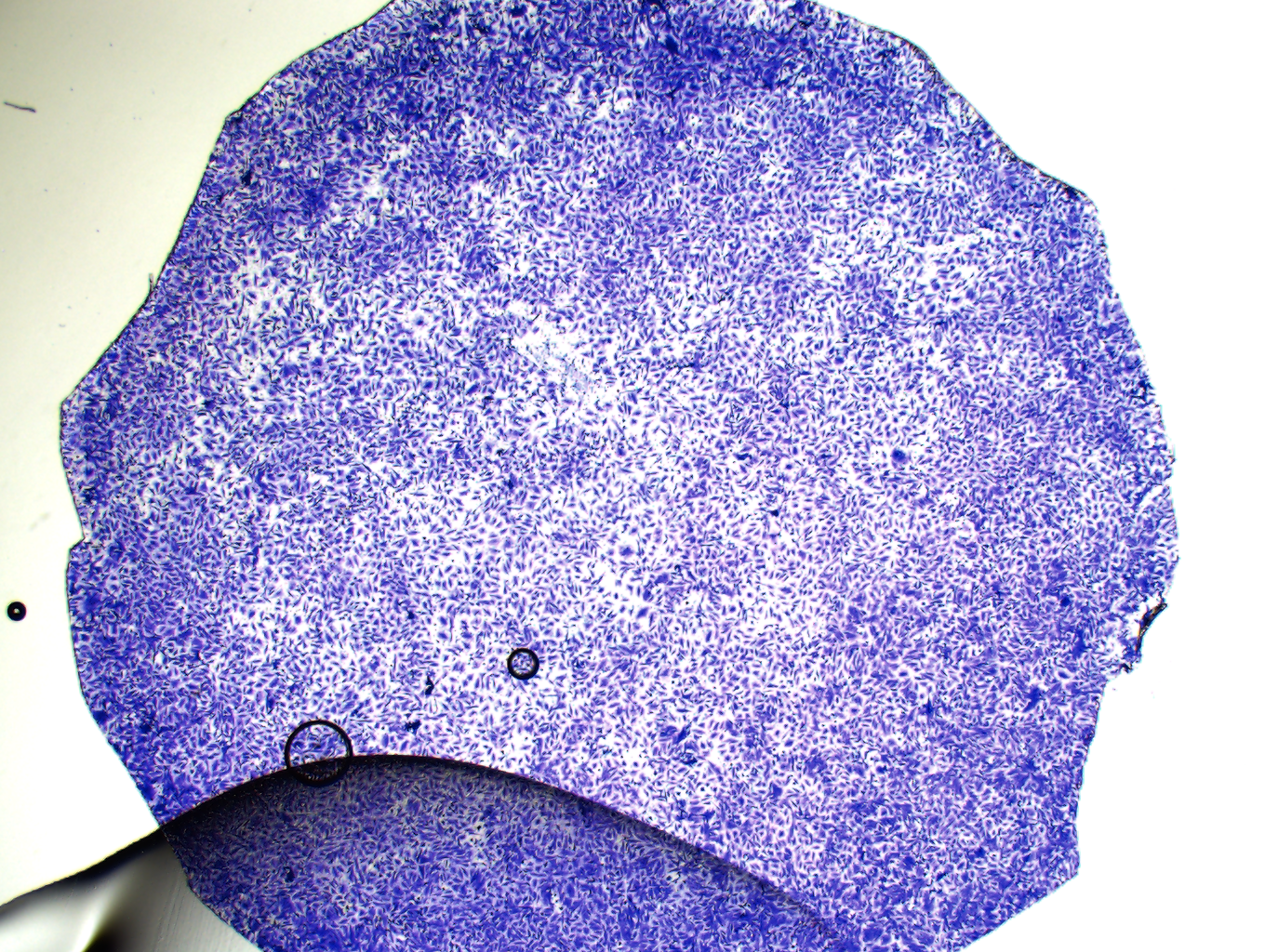

Supplement: Supplementary file 11 — Source data Fig. 6 [file 44321_2025_287_MOESM11_ESM.zip › Figure 6/6H/Transwell LN229 Nuak2 OE.tif]

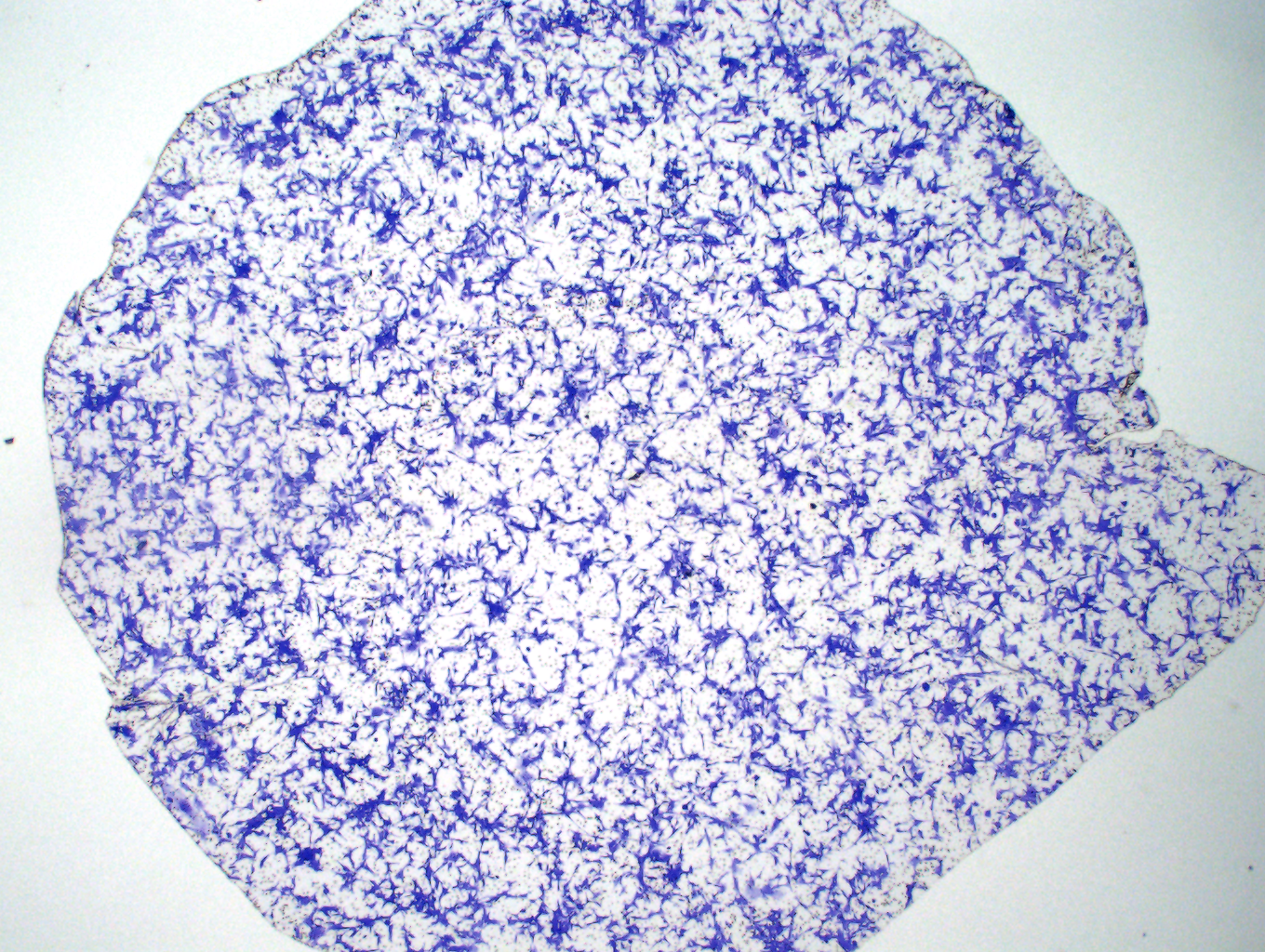

Supplement: Supplementary file 11 — Source data Fig. 6 [file 44321_2025_287_MOESM11_ESM.zip › Figure 6/6H/Transwell U87 WT.tif]

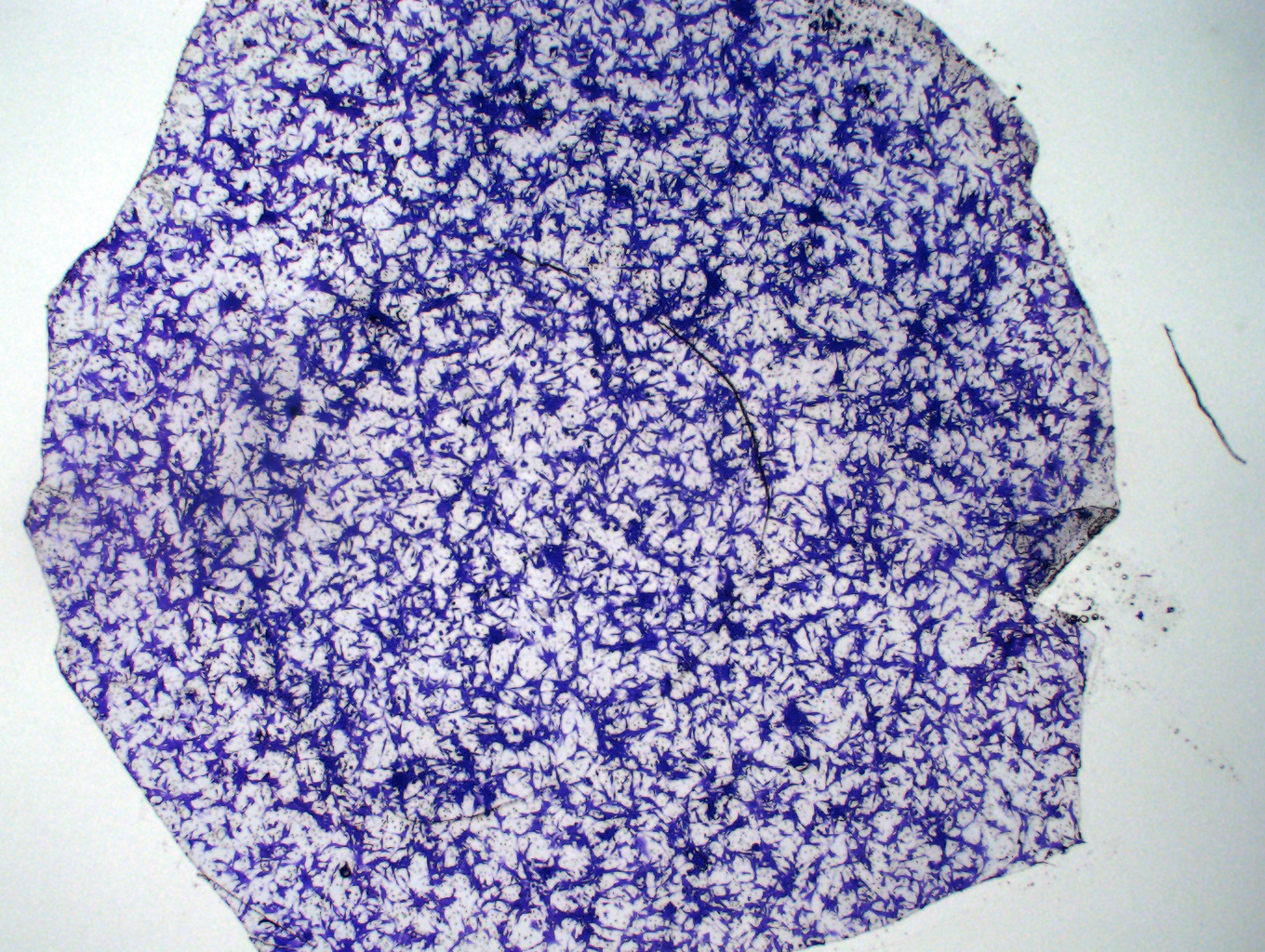

Supplement: Supplementary file 11 — Source data Fig. 6 [file 44321_2025_287_MOESM11_ESM.zip › Figure 6/6H/Transwell U87 Nuak2 OE.tif]
