# Supplementary material for: A fetal oncogene NUAK2 is an emerging therapeutic target in glioblastoma
Source: EMBO Mol Med. 2025 Aug 6;17(9):2409–37. doi: 10.1038/s44321-025-00287-3 (PMC12423323; doi:10.1038/s44321-025-00287-3)
Supplement: Supplementary file 12 — Expanded View Figures [file 44321_2025_287_MOESM12_ESM.pdf]

Expanded View Figures

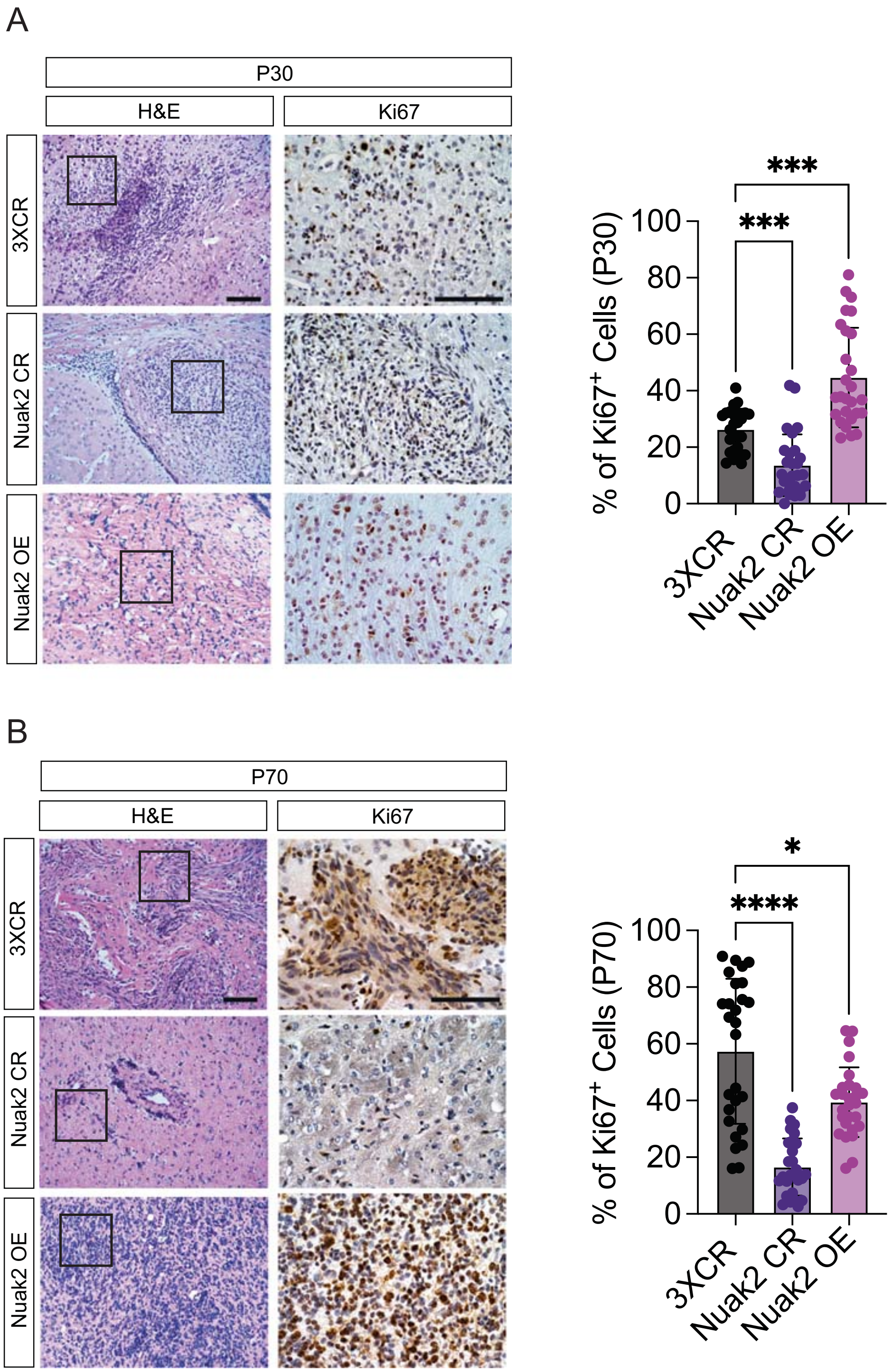

Figure EV1. Histological analysis of P30 and P70 IUE.

(**A**) Representative images of H&E and Ki67 proliferating cells in control, NUAK2-deleted or OE tumors at P30. Scale bar = 100 µm. Quantification analysis of Ki67-positive cells is represented as mean ± SD (***$p = 0.0001$; Statistical significance was determined by two-way RM ANOVA analysis followed by Dunnett's multiple comparisons test). Three images from three independent brains were used for quantification. (**B**) Representative images of H&E and Ki67 proliferating cells in control, NUAK2 deleted or OE tumors at P70. Scale bar = 100 µm. Quantification analysis of Ki67-positive cells is represented as mean ± SD (*$p = 0.016$, ****$p = 3.5E\text{-}10$; Statistical significance was determined by two-way RM ANOVA analysis followed by Dunnett's multiple comparisons test). Three images from three independent brains were used for quantification.

© The Author(s)

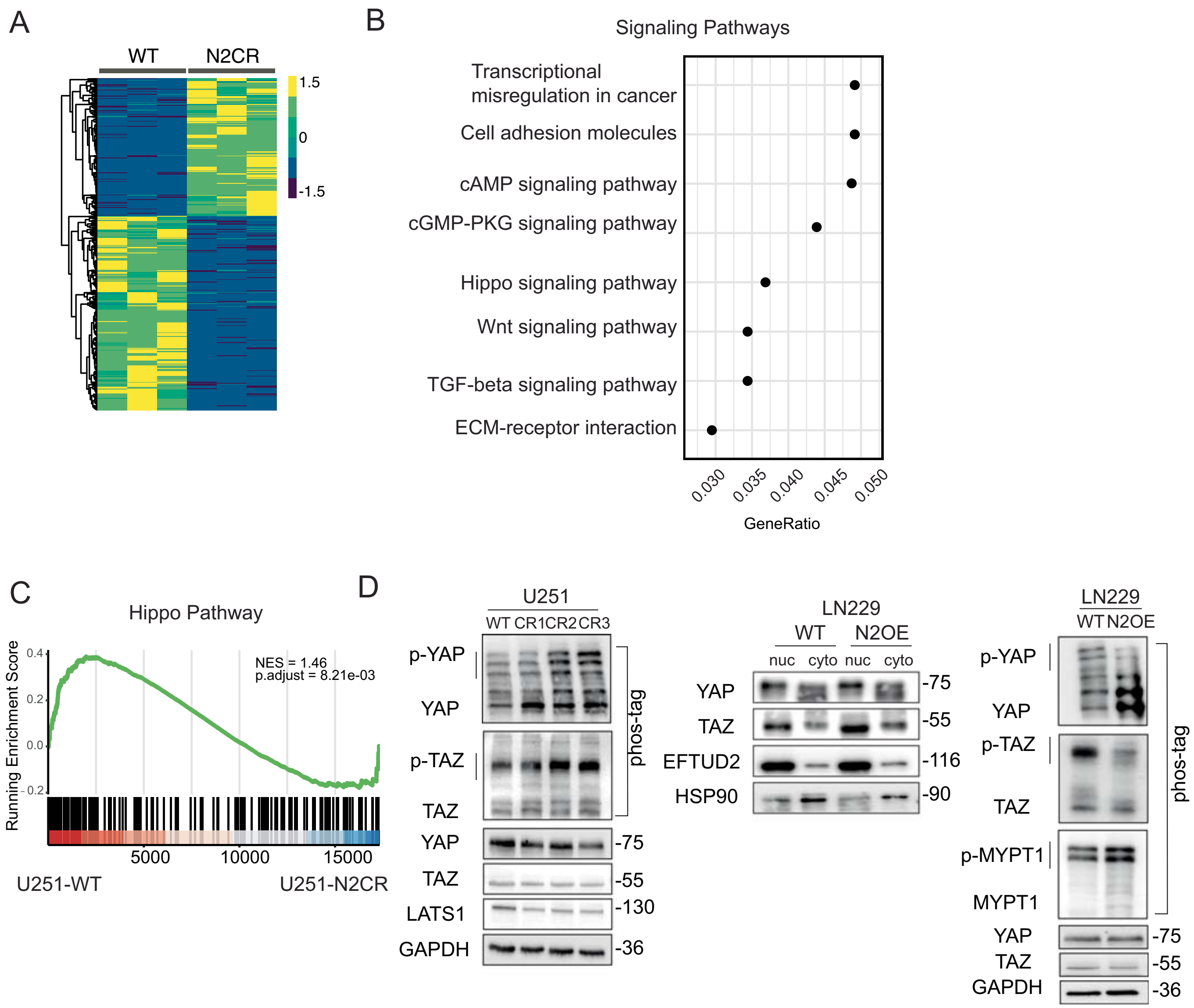

Figure EV2. NUAK2 association with the Hippo signaling pathway.

(**A**) Heatmap of differentially expressed genes in U251-control and U251 NUAK2 CRISPR-deleted cells. (**B**) KEGG pathway analysis of U251 NUAK2-CR DEGs. (**C**) GSEA enrichment plots of U251 NUAK2-CR DEGs showing enrichment in genes associated with the Hippo signaling pathway. Enrichment significance was assessed using the false discovery rate (FDR) method, and adjusted p-values (padj) were calculated using the Benjamini–Hochberg procedure. (**D**) Representative western blots analyzing downstream components of the Hippo pathway. Left panels: phos-tag western blots separating phosphorylated and non-phosphorylated YAP and TAZ in NUAK2-CR clones. Total YAP, TAZ, and LATS1 are also represented using a standard Western blot. GAPDH was used as a loading control. Molecular weights are shown to the right. Middle panels: standard Western blots of nuclear versus cytoplasmic fractions in LN229 NUAK2 overexpression lysates. Nuclear enrichment of YAP and TAZ is shown in the upper blots. Lower blots for EFTUD2 (nuclear) and HSP90 (cytoplasmic) confirm fractions. Right panels: phos-tag western blots separating phosphorylated and non-phosphorylated YAP and TAZ in LN229 NUAK2 overexpression lysates. Phos-tag blot showing enrichment of p-MYPT1 confirms that overexpressed NUAK2 is active in LN229 cells. Total YAP and TAZ are also represented using a standard Western blot. GAPDH was used as a loading control. Molecular weights are indicated to the right of the image.

© The Author(s)

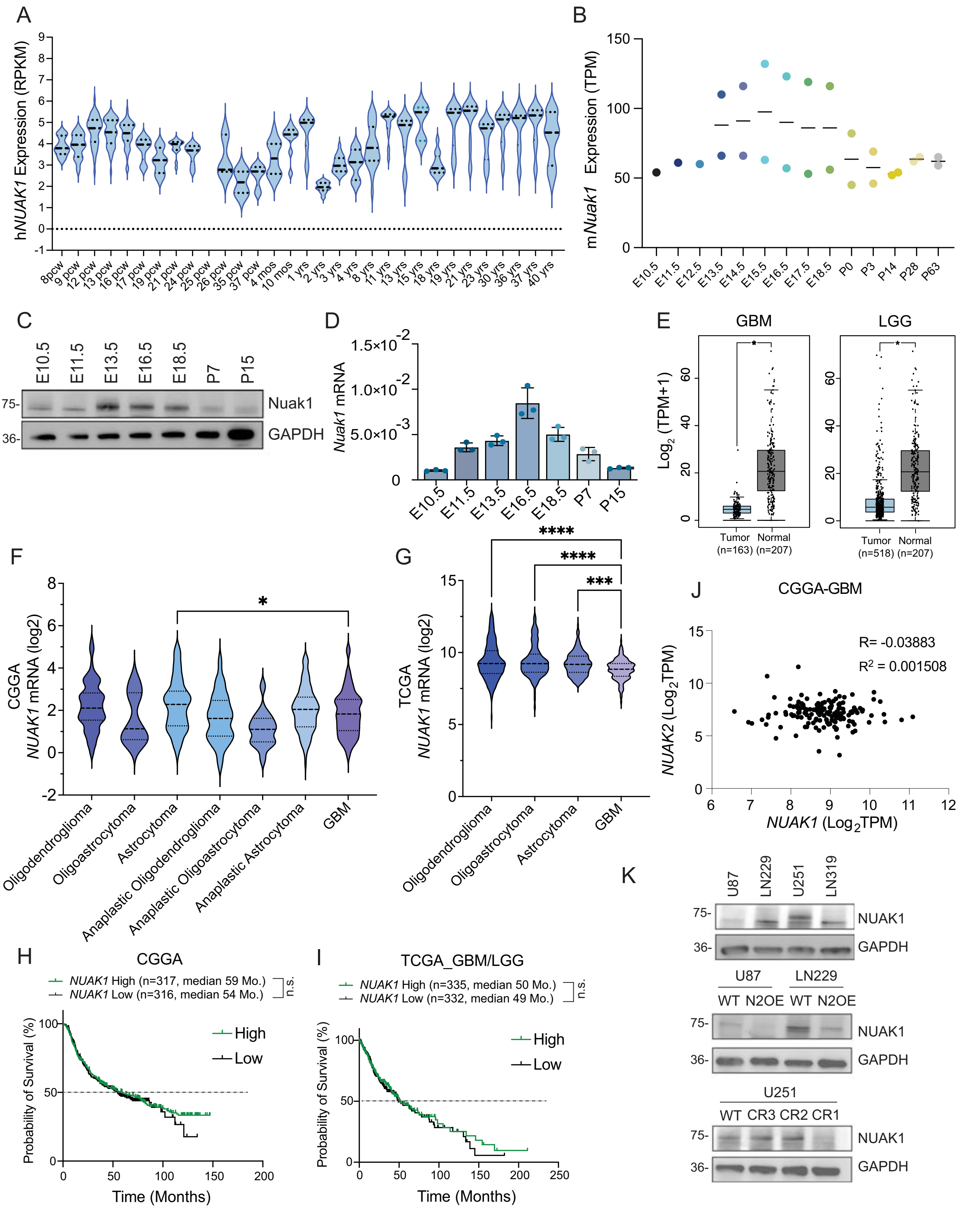
A
hNUAK1 Expression (RPKM)
B
mNuak1 Expression (TPM)
C
E10.5 E11.5 E13.5 E16.5 E18.5 P7 P15
Nuak1
GAPDH
D
Nuak1 mRNA
E
GBM
LGG
Log2 (TPM+1)
Tumor (n=163) Normal (n=207)
Tumor (n=518) Normal (n=207)
F
CGGA NUAK1 mRNA (log2)
Oligodendroglioma
Oligoastrocytoma
Astrocytoma
Anaplastic Oligodendroglioma
Anaplastic Oligoastrocytoma
Anaplastic Astrocytoma
GBM
G
TCGA NUAK1 mRNA (log2)
J
CGGA-GBM
R= -0.03883
R2 = 0.001508
NUAK2 (Log2TPM)
NUAK1 (Log2TPM)
K
U87 LN229 U251 LN319
NUAK1
GAPDH
WT N2OE WT N2OE
WT CR3 CR2 CR1
H
CGGA
NUAK1 High (n=317, median 59 Mo.)
NUAK1 Low (n=316, median 54 Mo.)
n.s.
Probability of Survival (%)
Time (Months)
High
Low
I
TCGA_GBM/LGG
NUAK1 High (n=335, median 50 Mo.)
NUAK1 Low (n=332, median 49 Mo.)

Figure EV3. NUAK1 is not associated with GBM progression and patient survival.

(**A**) RPKM-normalized NUAK1 mRNA expression of specific human brain regions from eight post-conception weeks (pcw) to 40 years of age. Data were obtained from the BrainSpan Atlas. $n = 5$–17. (**B**) TPM-normalized NUAK1 mRNA expression of mouse forebrain or hindbrain, ranging from embryonic day 10.5 to postnatal day 63. Data was obtained from EMBL's European Bioinformatics Institute (EMBL-EBI; https://www.ebi.ac.uk/). (**C**) Representative western blot of NUAK1 protein expression in wild-type embryonic brain tissue across seven stages of development. GAPDH was used as the loading control. Nuak1 is 74 kDa and Gapdh is 36 kDa. (**D**) Representative RT-PCR of NUAK1 mRNA expression in wild-type embryonic brain tissues across developmental stages. (**E**) Normalized NUAK1 mRNA expression of TCGA GBM ($n = 163$) or LGG ($n = 518$) and GTEx non-tumor ($n = 207$) samples ($*p = 0.01$; Statistical significance is determined by one-way ANOVA). Box plots display the median (center line), interquartile range (box: 25th to 75th percentiles), and whiskers representing the minimum and maximum values. Data were obtained from GEPIA (http://gepia.cancer-pku.cn/). (**F**) NUAK1 mRNA expression across glioma subtypes in the CGGA dataset. Data were represented as mean ± SD ($*p = 0.0193$; Statistical significance is determined by one-way ANOVA followed by Tukey's multiple comparisons test). $n = 8$–225 depending on the grade of the tumor. Data were obtained from the GlioVis Database. (**G**) NUAK1 mRNA expression across glioma subtypes in the TCGA dataset. Data were represented as mean ± SD ($***p < 0.001$, $****p < 0.0001$; Statistical significance is determined by one-way ANOVA followed by Tukey's multiple comparisons test). Exact p values are reported in Appendix Table S3. $n = 130$–194, depending on the grade of the tumor. Data were obtained from the GlioVis Database. (**H**) Kaplan–Meier survival analysis from CGGA of high (21 days; $n = 317$) and low (145 days; $n = 316$) NUAK1 expressers shows no correlation with survival outcomes ($p = 0.682$; Statistical significance was determined by log-rank (Mantel–Cox) test). (**I**) Kaplan–Meier survival analysis from TCGA of high (15 days; $n = 335$) and low (134 days; $n = 332$) NUAK2 expressers shows no correlation with survival outcomes ($p = 0.6262$; Statistical significance was determined by log-rank (Mantel–Cox) test). (**J**) Spearman's rank coefficient plots demonstrating no correlation between NUAK1 and NUAK2 expression in human gliomas. Analyzed data were obtained from the CGGA GBM dataset. ($p = 0.6337$). (**K**) Top panels: Representative western blots of NUAK1 expression in U87, LN229, U251, and LN219. Middle panels: Western blots of NUAK1 expression in U87-N2OE and LN229-N2OE cells. Bottom panels: NUAK1 expression in U251-CRISPR-deleted clones. GAPDH was used as the loading control. Molecular weights are shown to the left of the blots.

 © The Author(s)

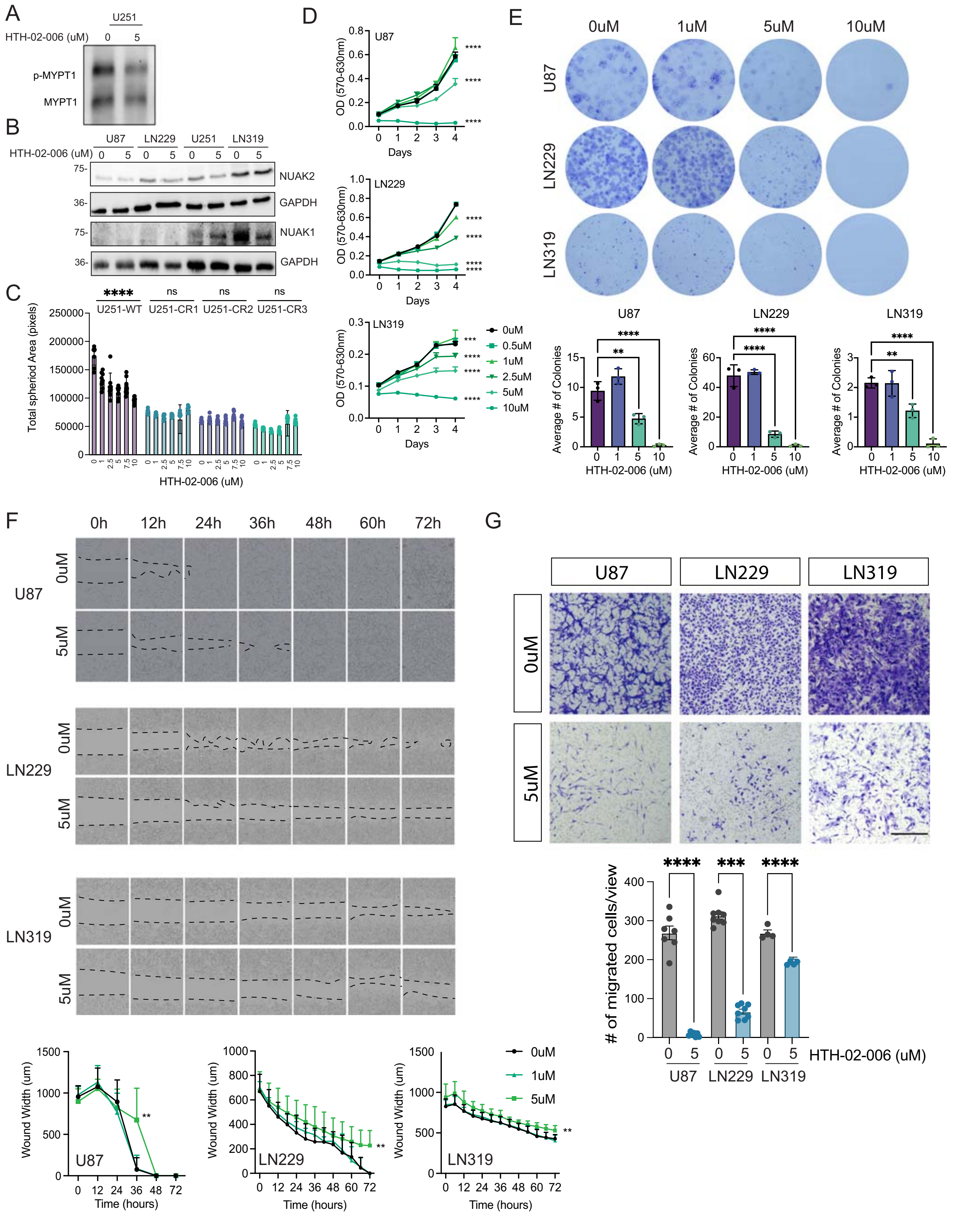
A
U251
HTH-02-006 (uM) 0 5
p-MYPT1
MYPT1
B
U87 LN229 U251 LN319
HTH-02-006 (uM) 0 5 0 5 0 5 0 5
NUAK2
GAPDH
NUAK1
GAPDH
C
**** ns ns ns
U251-WT U251-CR1 U251-CR2 U251-CR3
Total spheriod Area (pixels)
HTH-02-006 (uM)
D
U87
LN229
LN319
OD (570-630nm)
Days
0uM
0.5uM
1uM
2.5uM
5uM
10uM
E
0uM 1uM 5uM 10uM
U87
LN229
LN319
Average # of Colonies
HTH-02-006 (uM)
F
0h 12h 24h 36h 48h 60h 72h
0uM
5uM
U87
LN229
LN319
Wound Width (um)
Time (hours)
0uM
1uM
5uM
G
U87 LN229 LN319
0uM
5uM
# of migrated cells/view
HTH-02-006 (uM)

Figure EV4. NUAK2 inhibitor, HTH-02-006, attenuates GBM cell progression.

(**A**) Representative phos-tag western blot of MYPT1 on lysates from U251 HTH-02-006 or vehicle-treated cells. HTH-02-006 creased phosphorylation of MYPT1. (**B**) Representative western blot of NAUK1 and NUAK2 expression in U87, LN229, U251, and LN319 cells treated with either 5 uM HTH-02-006 or DMSO vehicle control. GAPDH was used as a loading control. Molecular weights are shown to the left of the blot. (**C**) Quantification of total spheroid area of HTH-02-006-treated U251 spheroids grown for 6 days. Data were represented as mean ± SD ($n = 8$; ****$p < 0.0001$; Statistical significance was determined by one-way ANOVA analysis followed by trend testing. Exact p values are reported in Appendix Table S3. (**D**) MTT assay for proliferation in HTH-02-006-treated U87 ($n = 6$), LN229 ($n = 6$), and LN219 ($n = 6$) cells. Data were represented as mean ± SD (***$p = 0.0008$, ****$p < 0.0001$; Statistical significance was determined by two-way RM ANOVA followed by Dunnett's multiple comparison test. Exact p values are reported in Appendix Table S3. (**E**) Representative images of colony formation assay of U87 ($n = 3$), LN229 ($n = 3$), and LN319 ($n = 3$) cells with HTH-02-006 treatment. Quantification of colony formation assay (**$p < 0.01$, ****$p < 0.0001$; Statistical significance was determined by one-way ANOVA followed by Dunnett's multiple comparison test). Exact p values are reported in Appendix Table S3. Data were represented as mean ± SD. (**F**) Representative images and quantification of HTH-02-06-treated U87, LN229, and LN319 cell migration into the wound area. Data were represented as mean ± SD (**$p < 0.01$; Statistical significance was determined by two-way RM ANOVA followed by Dunnett's multiple comparison test). Exact p values are reported in Appendix Table S3. The white dotted lines demarcate the wound boundary. Scale bar = 100 µm. (**G**) Representative images and quantification of transwell migration assay after HTH-02-006 treatment of U87 ($n = 7$), LN229 ($n = 8$), LN319 ($n = 4$) cells. Data were represented as mean ± SD (***$p < 0.001$, ****$p < 0.0001$; Statistical significance is determined by unpaired t-test (two-tailed)). Exact p values are reported in Appendix Table S3. Scale bar = 500 µm.

 © The Author(s)

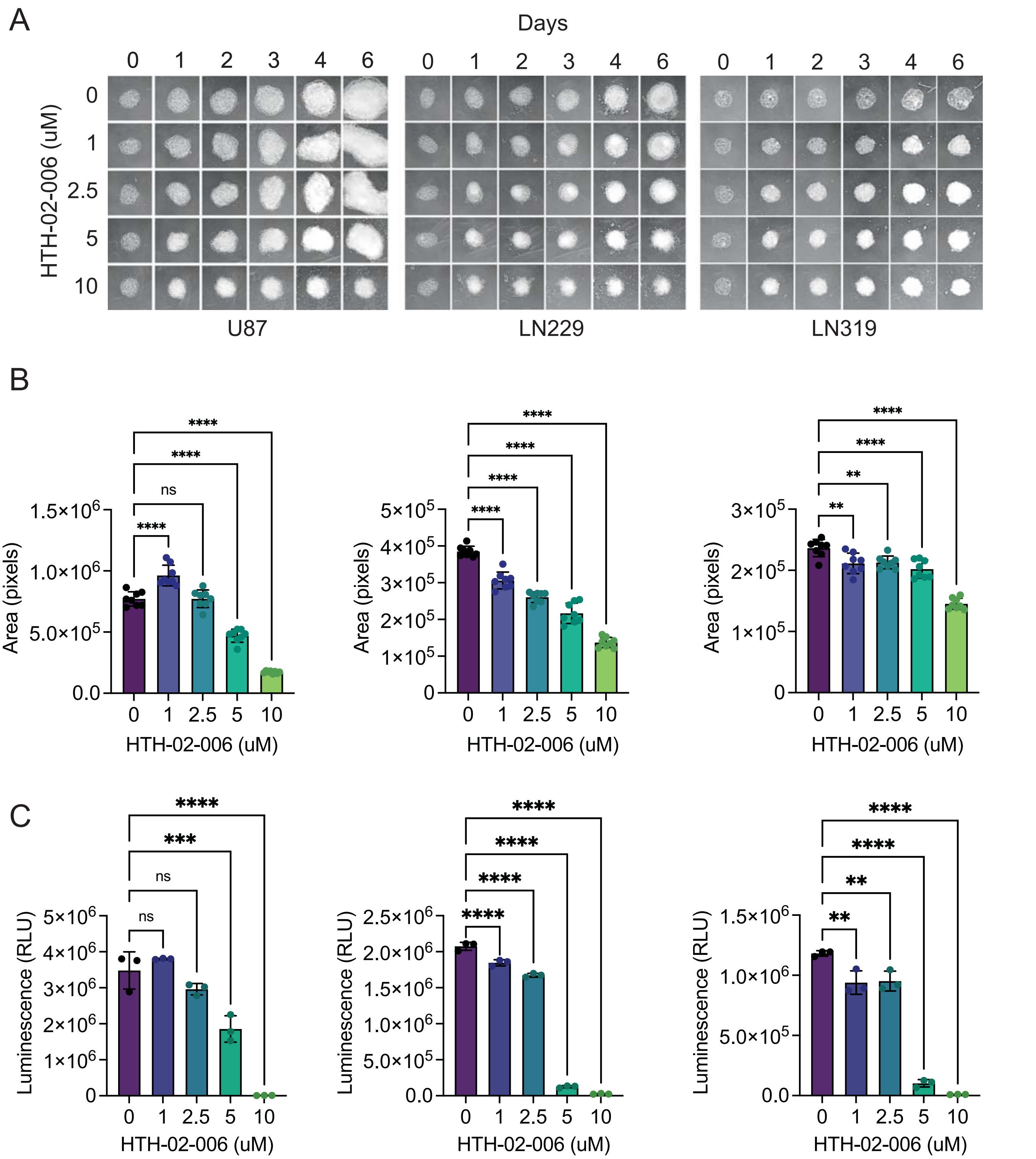

Figure EV5. Efficacy of HTH-02-006 in 3D GBM spheroids.

(**A**) Representative brightfield images of spheroid assay in HTH-02-06-treated U87, LN229, and LN319 cells over the course of 6 days. (**B**) Quantification of total spheroid area of HTH-02-06-treated U87, LN229, and LN319 cells spheroids. Data were represented as mean ± SD ($n = 8$, ** $p < 0.01$, **** $p < 0.0001$; Statistical significance was determined by one-way ANOVA followed by Dunnett's multiple comparison test. Exact p values are reported in Appendix Table S3. (**C**) Luminescence intensity of viable cells in HTH-02-06-treated U87, LN229, and LN319 spheroids at day 6. Data were represented as mean ± SD ($n = 3$, ** $p < 0.01$, *** $p < 0.001$, **** $p < 0.0001$; Statistical significance was determined by one-way ANOVA followed by Dunnett's multiple comparison test. Exact p values are reported in Appendix Table S3.

© The Author(s)
